# Catalytic enantioselective Henry reaction of α-keto esters, 2-acylpyridines and 2-acylpyridine *N*-oxides†

**DOI:** 10.1039/c8ra00552d

**Published:** 2018-03-05

**Authors:** Feilong He, Guanghui Chen, Junxia Yang, Guojuan Liang, Ping Deng, Yan Xiong, Hui Zhou

**Affiliations:** School of Pharmaceutical Science, Chongqing Medical University Chongqing 400016 PR China hzhou@cqmu.edu.cn; School of Chemistry and Chemical Engineering, Chongqing University Chongqing 401331 PR China

## Abstract

A pre-prepared Ni–PyBisulidine complex has been developed for the catalytic asymmetric Henry reaction of α-keto esters, 2-acylpyridines and 2-acylpyridine *N*-oxides. The corresponding β-nitro-α-hydroxy esters were obtained in good to excellent yields (up to 99%) with a high enantiomeric excess (ee) (up to 94%) with a catalyst loading of 1–2 mol%. The desired products of 2-acylpyridines and 2-acylpyridine *N*-oxides, which were simple methyl ketones, were obtained in medium to excellent yields (up to 94%) with medium to good ee (up to 86%) by using 2 mol% of catalyst.

## Abstract 2

Asymmetric Henry reaction of ketones catalyzed by a pre-prepared Ni complex with low catalyst loading.

## Graphical abstract

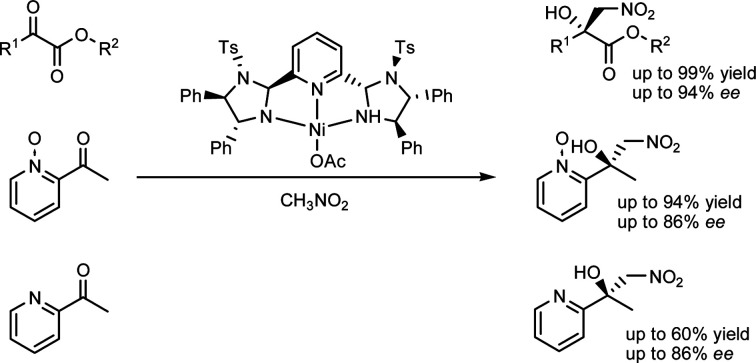

## Introduction

The Henry reaction is one of the important methods for C–C bond formation.^1^ The resulting products, β-nitroalcohols, are key intermediates and building blocks for the synthesis of bioactive natural products and pharmaceutical agents.^1^ Thus, increasing efforts have been directed towards developing a catalytic asymmetric Henry reaction.^2^ Compared with the well developed asymmetric Henry reaction of aldehydes, the asymmetric Henry reaction of ketones with the formation of a quaternary stereogenic center is more challenging because it often suffers from low reactivity and poor stereoselectivity.^3^ Although Tosaki *et al.* reported the kinetic resolution of racemic derivatives,^4^ the catalytic asymmetric Henry reaction of simple ketones is rarely reported. At present, the study mainly focused on reactive substrates such as trifluoromethyl ketones,^5^ α-keto esters,^6^ α-keto amides,^7^ α-keto-phosphonates,^8^ and glyoxal hydrates.^9^ Holmquist *et al.* expanded the scope of this reaction to 2-acylpyridine *N*-oxide, simple ketones, for the first time.^10^ Although great progress has been achieved, several factors, including the relatively high catalyst loading (5–20 mol%) or catalyst preparation, limit the use of existing catalytic methods. At the same time, developing new catalysts for the enantioselective Henry reaction of ketones is still necessary. Recently a sulfonylated pyridine bisimidazolidine: nickel–pyridine bisulidine (Ni–PyBisulidine) complex was introduced for the asymmetric hydrophosphonylation of aldehydes.^11–13^ In this paper, the use of pre-prepared Ni–PyBisulidine complexes for the asymmetric Henry reaction of α-keto esters, 2-acylpyridines and 2-acylpyridine *N*-oxides with low catalyst loading (down to 1 mol%) is reported.

## Results and discussion

**Fig. 1:**
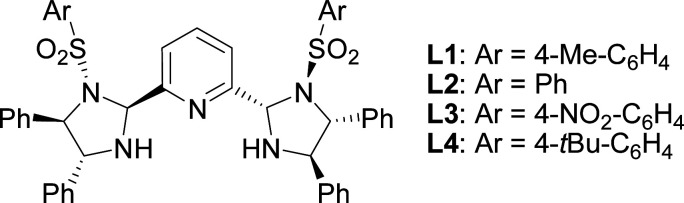
Chiral PyBisulidine used as ligands for the asymmetric Henry reaction.

The initial studies of the catalytic asymmetric Henry reaction focused on the addition of nitromethane (CH_3_NO_2_) to methyl phenyloxoacetate in the presence of the complex of chiral PyBisulidine L1 as ligand (Fig. 1). The complexes of nickel(ii) acetate–L1 [Ni(OAc)_2_–L1], cobalt(ii) acetate–L1 [Co(OAc)_2_–L1], zinc(ii) acetate–L1 [Zn(OAc)_2_–L1] promoted the reaction in a 70–86% enantiomeric excess (ee) with low yields at room temperature (rt; Table 1, entries 1, 3 and 5). When nickel(ii) acetylacetonate [Ni(acac)_2_] and copper(ii) acetate [Cu(OAc)_2_] were used as the central metal, a low chiral induction was observed (Table 1, entries 2 and 4). When the complexes of iron(ii) acetate–L1 [Fe(OAc)_2_–L1] and palladium(ii) acetate–L1 [Pd(OAc)_2_–L1] were used as the catalysts, the corresponding products were not detected (Table 1, entries 6 and 7). Fortunately, the Ni(OAc)_2_–L1 complex could catalyze this reaction smoothly with 83% ee with a 85% yield when the reaction temperature rose to 35 °C (Table 1, entry 8). However, further increasing the reaction temperature did not improve the reactivity (Table 1, entry 9). After screening the benzenesulfonyl moiety of the ligands (Table 1, entries 8, 10–12), Ni(OAc)_2_–L1 was selected for further optimization which considered the reactivity and economy.

**Table 1:** Screening of central metals, PyBisulidine ligands and temperature in the asymmetric Henry reaction of methyl phenyloxoacetate^a^

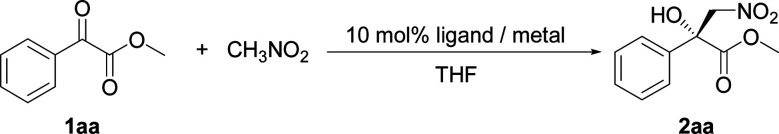					
Entry	Metal	Ligand	*T* (°C)	Yield^b^ (%)	ee^c^ (%)
1	Ni(OAc)_2_	L1	rt	16	86
2	Ni(acac)_2_	L1	rt	76	8
3	Co(OAc)_2_	L1	rt	37	75
4	Cu(OAc)_2_	L1	rt	18	8
5	Zn(OAc)_2_	L1	rt	19	70
6^d^	Fe(OAc)_2_	L1	rt	ND^e^	—
7^d^	Pd(OAc)_2_	L1	rt	ND^e^	—
8	Ni(OAc)_2_	L1	35	85	83
9	Ni(OAc)_2_	L1	50	84	80
10	Ni(OAc)_2_	L2	35	84	69
11	Ni(OAc)_2_	L3	35	82	88
12	Ni(OAc)_2_	L4	35	62	72

a Reactions were carried out using methyl phenyloxoacetate (0.2 mmol) with CH_3_NO_2_ (0.2 mL) in THF (0.8 mL) in the presence of metal–PyBisulidines prepared *in situ* for 20 h.

b Isolated yield.

c Determined using HPLC analysis on a chiral stationary phase.

d The reaction time was 65 h.

e ND: not detected.

The influence of the ester group in the substrate was tested next (Table 2, entries 1–3). The best result in terms of the conversion and enantioselectivity was obtained with the isopropyl ester (Table 2, entry 3). The pre-prepared complex^14^ gave better results than the complex prepared *in situ* (Table 2, compare entries 3 and 4).

**Table 2:** Screening of the ester group R and catalyst preparation method^a^

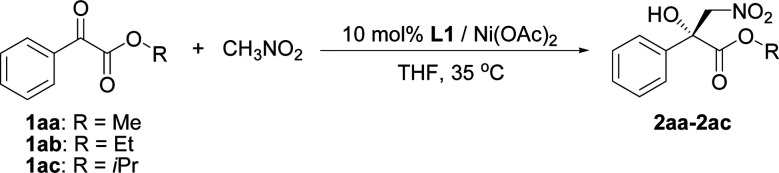				
Entry	R	Catalyst preparation method^b^	Yield^c^ (%)	ee^d^ (%)
1	Me	*In situ*	85 (2aa)	83
2	Et	*In situ*	62 (2ab)	86
3	i-Pr	*In situ*	83 (2ac)	88
4	i-Pr	Pre-prepared	85 (2ac)	91

a Reactions were carried out using an α-keto ester (0.2 mmol) in a mixture of THF (0.8 mL) and CH_3_NO_2_ (0.2 mL) for 20 h.

b For details, see ref. 11.

c Isolated yield.

d Determined using HPLC analysis on a chiral stationary phase.

Encouraged by the initial results in the asymmetric Henry reaction, various solvents were screened in the presence of 10 mol% pre-prepared Ni–L1 (Table 3). Whereas small or trace amounts of products were detected in chloroform (CHCl_3_) or 1,2-dimethoxyethane (DME; Table 3, entries 2 and 5). Other solvents tested, such as methanol (CH_3_OH, MeOH), toluene and diethylene glycol dimethyl ether, gave moderate yields (Table 3, entries 3, 4 and 6). Tetrahydrofuran (THF) exhibited the best performance (Table 3, entry 1).

**Table 3:** Screening of the solvents used in the asymmetric Henry reaction of isopropyl phenyloxoacetate^a^

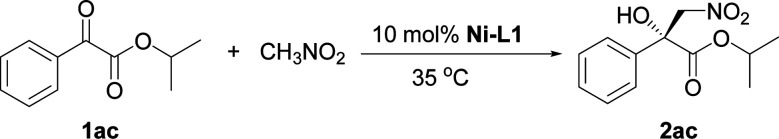			
Entry	Solvent	Yield^b^ (%)	ee^c^ (%)
1	THF	85	91
2	CHCl_3_	37	89
3	CH_3_OH	56	43
4	Toluene	68	90
5	DME	Trace	—
6	Diglyme^d^	62	91

a Reactions were carried out on a 0.2 mmol scale of isopropyl phenyloxoacetate in the mixture of THF (0.8 mL) and CH_3_NO_2_ (0.2 mL) for 20 h. The catalyst was pre-prepared.

b Isolated yield.

c Determined by HPLC analysis on a chiral stationary phase.

d Diglyme = diethylene glycol dimethyl ether.

When the catalyst loading was reduced, the reactivity decreased sharply with slightly increasing ee (Table 4, entries 1–4). To increase the reactivity, some additives were screened in this reaction (Table 4, entries 5–8). The yields of Henry products were improved when using a 4 Å molecular sieve (MS; Table 4, entries 7 and 11) and addition of *tert*-amines (Table 4, entries 5, 6, 9 and 10). However, some acidic additives, such as benzoic acid (PhCOOH), were harmful for the reaction (Table 4, entry 8).

**Table 4:** Screening the effects of reducing the amount of catalyst loading, additive and base in the asymmetric Henry reaction of isopropyl phenyloxoacetate^a^

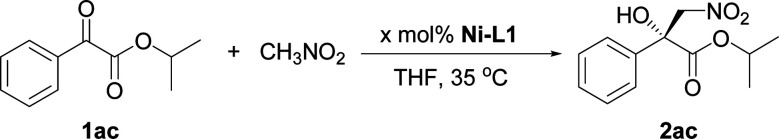					
Entry	Catalyst loading	Base (mol%)	Additive	Yield^b^ (%)	ee^c^ (%)
1	10 mol%	None	None	85	91
2	5 mol%	None	None	80	92
3	2 mol%	None	None	49	93
4	1 mol%	None	None	28	94
5	10 mol%	iPr_2_NEt^d^ (10)	None	92	86
6	10 mol%	*N*-Methyl-morpholine (10)	None	90	89
7	10 mol%	None	4 Å MS^e^ (38 mg)	95	86
8	10 mol%	None	PhCOOH (10 mol%)	46	90
9	2 mol%	iPr_2_NEt (10)	None	69	88
10	2 mol%	*N*-Methyl-morpholine (10)	None	66	92
11	2 mol%	None	4 Å MS (38 mg)	56	93
12	2 mol%	*N*-Methyl-morpholine (10)	4 Å MS (20 mg)	80	93
13	2 mol%	*N*-Methyl-morpholine (10)	4 Å MS (30 mg)	92	93
14	2 mol%	*N*-Methyl-morpholine (10)	4 Å MS (50 mg)	90	93

a Reactions were carried out using scale of isopropyl phenyloxoacetate (0.2 mmol) in a mixture of THF (0.8 mL) and CH_3_NO_2_ (0.2 mL) for 20 h. The catalyst was pre-prepared.

b Isolated yield.

c Determined using HPLC analysis on a chiral stationary phase.

d *N*,*N*-Diisopropylethylamine.

e Molecular sieve.

When the catalyst loading was reduced to 2 mol%, *N*-methylmorpholine showed a better performance than iPr_2_NEt in terms of enantioselectivity (Table 4, entries 9 and 10). The amount of 4 Å MS was screened together with 10 mol% *N*-methylmorpholine (Table 4, entries 12–14). Under the optimized conditions (at 35 °C, in the presence of 2 mol% Ni–L1, 10 mol% *N*-methylmorpholine, and 150 mg mmol^−1^ 4 Å MS in THF), 2ac was obtained with a 92% yield with 93% ee (Table 4, entry 13).

The current catalytic system was applied to various α-keto esters (Table 5). In all cases, the reactions were clean and proceeded and gave good to excellent yields with high enantioselectivities. Aromatic keto esters bearing the electron-donating groups gave smaller yields but the high enantioselectivities were maintained (72–83% yield, Table 5, 2bc–2dc, and 2gc). Aromatic keto esters bearing the electron-withdrawing group gave excellent yields (Table 5, 2ec, and 2ic–2mc) and the catalyst loading could be reduced to 1 mol% with high to excellent yields and high enantioselectivities (Table 5, 2ic–2mc). The keto esters derived from the bulkier ketone, such as β-acetonaphthone, also gave an excellent yield and high enantioselectivity (Table 5, 2fc). The heteroaromatic and alkyl keto esters gave smaller ee values (Table 5, 2hc and 2nb). The configuration of 2ac was identified as *R* using single crystal diffraction analysis,^15^ and the configuration of the other products were inferred to be analogous with that of 2ac. It should be noted that the pre-prepared complex Ni–L1 can be stored in air at 4 °C for at least three months without any loss of activity.^16^

**Table 5:** Substrate scope of catalytic asymmetric Henry reaction of α-keto esters^a^

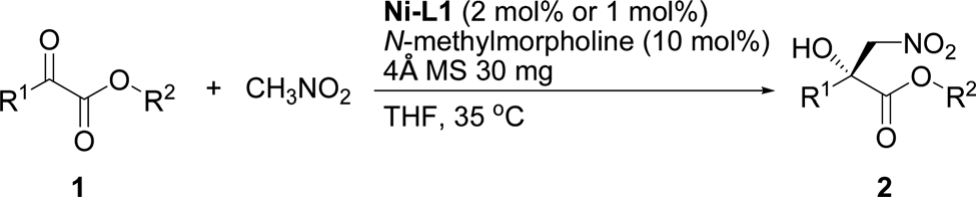
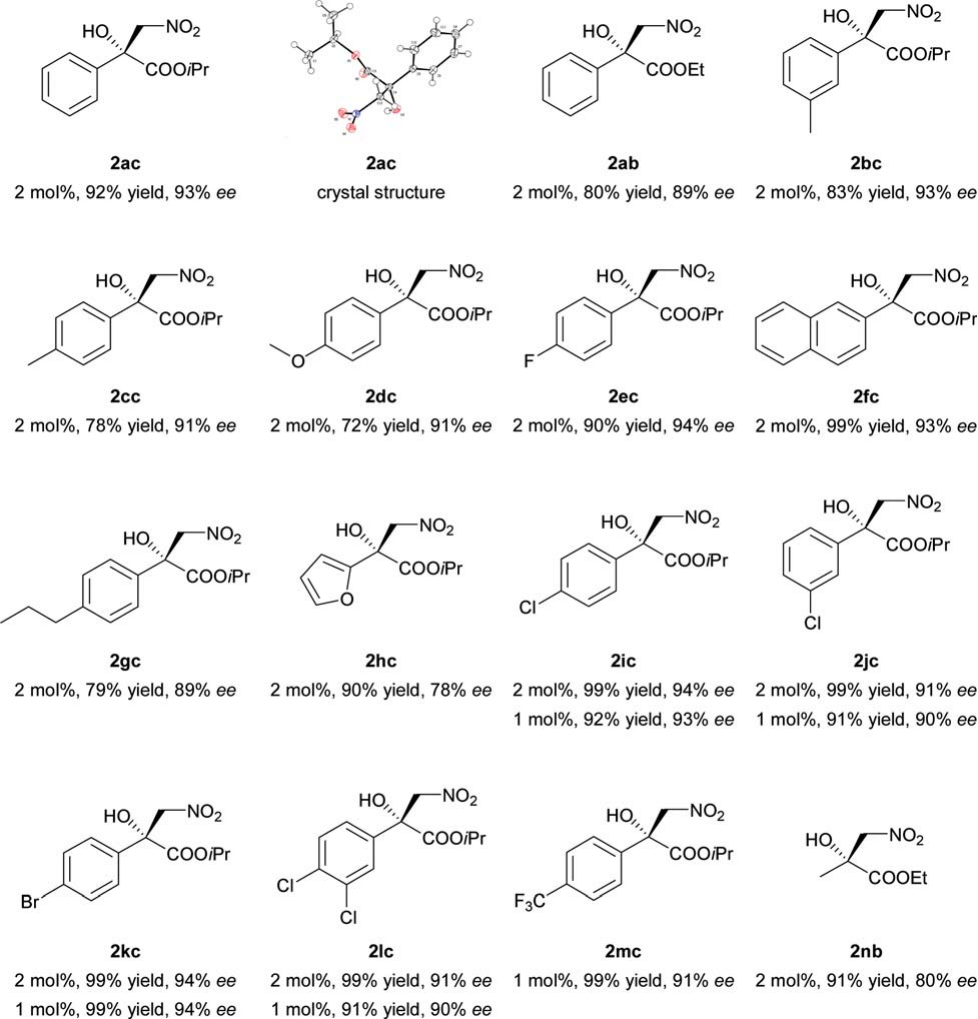

a Unless otherwise noted, all reactions were carried out with α-keto ester (0.2 mmol) with *N*-methylmorpholine (10 mol%) and 4 Å MS (30 mg) in a mixture of THF (0.8 mL) and CH_3_NO_2_ (0.2 mL) for 20 h. The catalyst was pre-prepared. The reaction time for 2dc, 2gc, and 2hc was 36 h.

The Ni–L1 was also used in the asymmetric Henry reaction of 2-acylpyridine *N*-oxides (Table 6).^17^ High yields and good ee were obtained with methyl ketones (Table 6, 4a–4d, 4f). Whereas low ee were obtained with ethyl ketones (Table 6, 4g). The corresponding product of 3-methyl-2-acylpyridine *N*-oxide was not detected (Table 6, 4e).

**Table 6:** Catalytic asymmetric Henry reaction of 2-acylpyridine *N*-oxides^a^

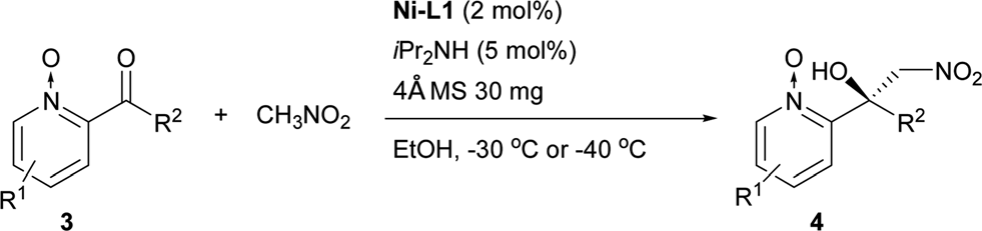
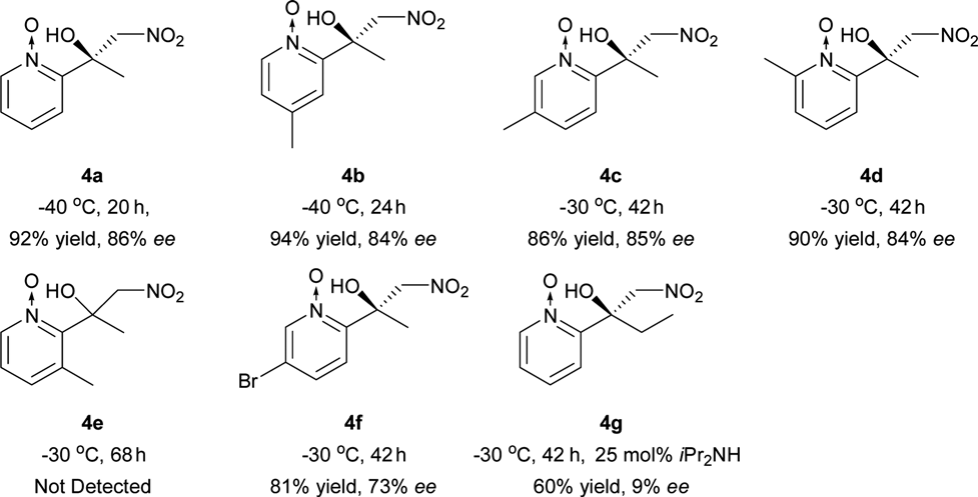

a Reactions were carried out with 2-acylpyridine *N*-oxides (0.2 mmol) with diisopropylamine (iPr_2_NH; 5 mol%) and 4 Å MS (30 mg) in a mixture of EtOH (0.8 mL) and CH_3_NO_2_ (0.2 mL) for 20–42 h. The catalyst was pre-prepared. EtOH: ethanol.

Inspired by the research of Tosaki *et al.*^4^ and Holmquist *et al.*^10^, the Henry reaction of 2-acylpyridines was also investigated (Table 7).^17^ In most cases, 50–60% yields and 70–86% ee were obtained with methyl ketones. Racemic products were obtained for ethyl ketones (Table 7, 6g). The Henry reaction of 3-methyl-2-acylpyridine did not take place at all. The results were similar to those obtained using 2-acylpyridine *N*-oxides, indicating that they had similar transition states.

**Table 7:** Catalytic asymmetric Henry reaction of 2-acylpyridine^a^

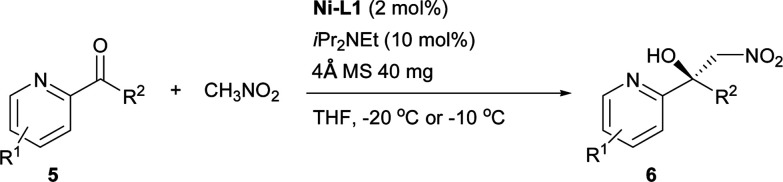
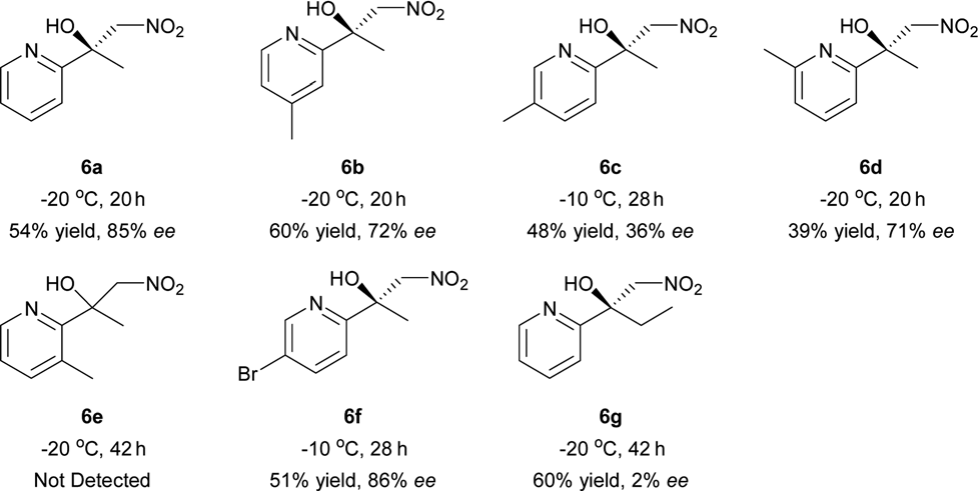

a Reactions were carried out with 2-acylpyridine (0.2 mmol) with iPr_2_NEt (10 mol%) and 4 Å MS (40 mg) in a mixture of THF (0.8 mL) and CH_3_NO_2_ (0.2 mL) for 20–42 h. The catalyst was pre-prepared.

**Fig. 2:**
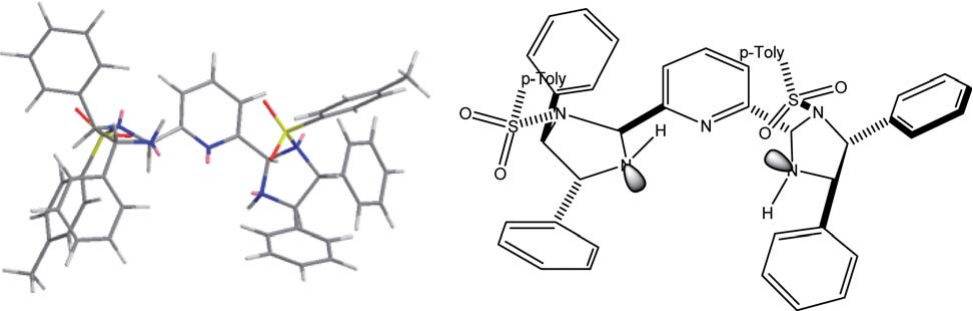
The geometry of L1 optimized using Chem3D (8.0) at the MM2 level.

**Fig. 3:**
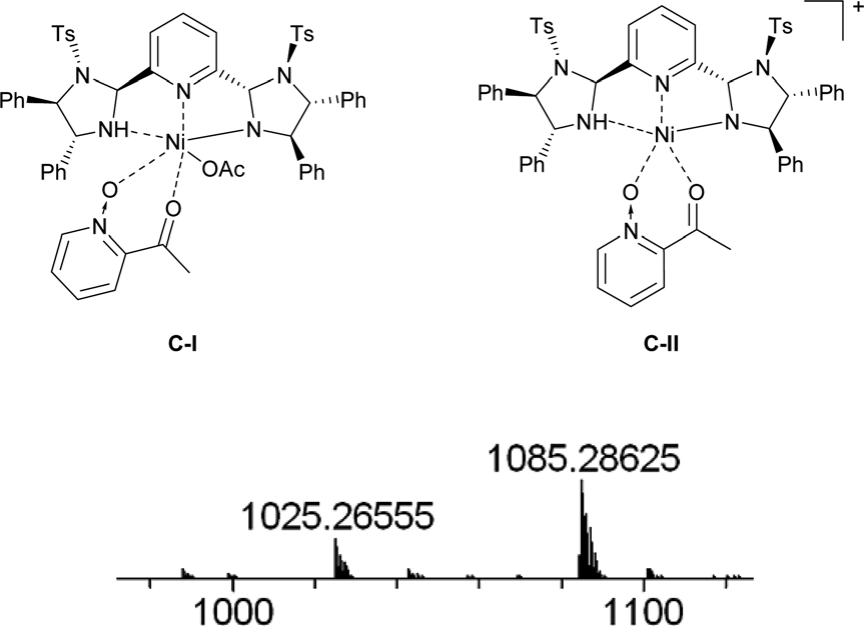
ESI-HRMS for C-I: *m*/*z* calc'd for C_58_H_55_N_6_NiO_8_S_2_^+^ [Ni(OAc)_2_ + M_L1_ + M_3a_ − HOAc + H]^+^: 1085.28708, found: 1085.28625; ESI-HRMS for C-II: *m*/*z* calc'd for C_56_H_51_N_6_NiO_6_S_2_^+^ [Ni(OAc)_2_ + M_L1_ + M_3a_ − HOAC − OAc]^+^: 1025.26595, found: 1025.26555.

**Fig. 4:**
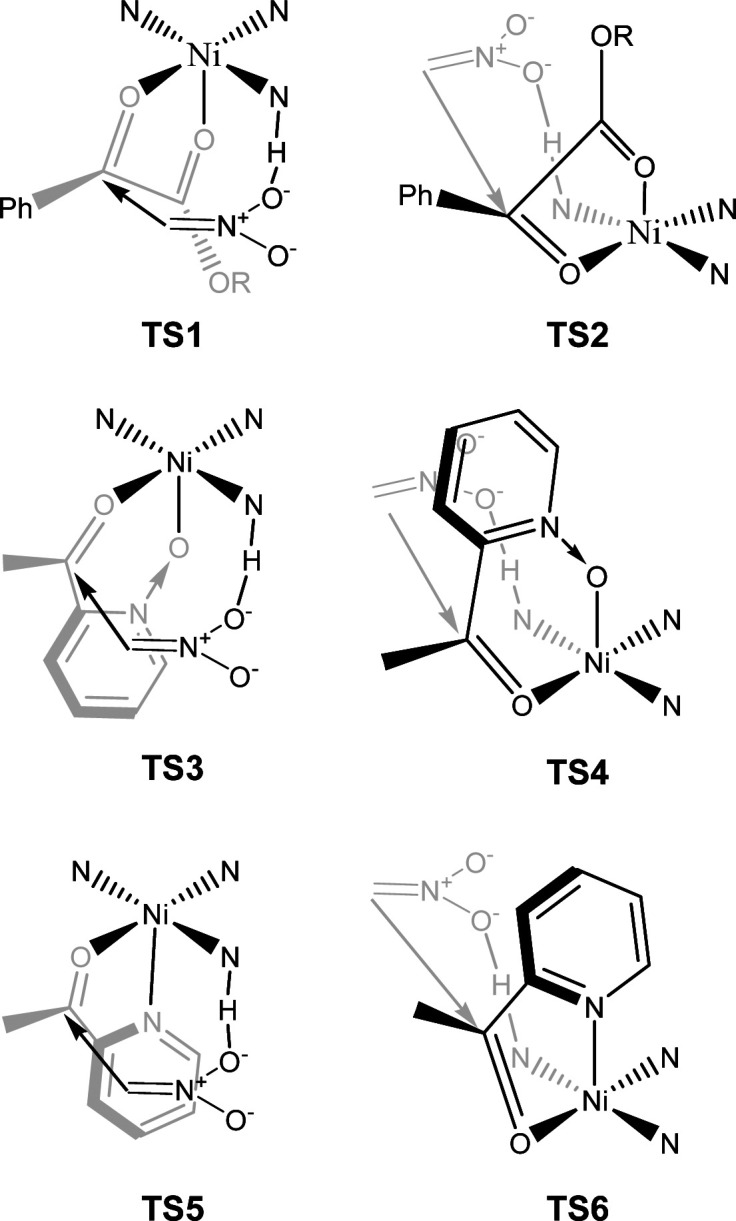
The proposed working model.

The proposed structure of Ni–L1 on the basis of the related structure of Fe–PyBisulidine complex,^12*a*^ the geometry of L1 optimized using Chem3D at the MM2 level (Fig. 2) and the electrospray ionization-high resolution mass spectrometry (ESI-HRMS) analysis of the complex has previously been reported.^11^ To gain some insight into the active species, ESI-HRMS studies of the mixture of Ni–L1 and 3a were carried out. The spectrum displayed ions at *m*/*z* 1085.28625 and 1025.26555, which corresponded to C-I and C-II (Fig. 3). It was speculated that the complex C-I or C-II would be the active species. TS1–TS6 are proposed to rationalize the asymmetric induction. As illustrated in Fig. 4, the keto functionality is coordinated to Ni(ii) in the more Lewis acidic equatorial position for maximal activation,^18^ whereas the nitronate generated by the amine is positioned by the hydrogen bonding.^13*g*,13*h*^

## Conclusions

A catalytic asymmetric Henry reaction of α-keto esters, 2-acylpyridines and 2-acylpyridine *N*-oxides, was developed using a Ni–PyBisulidine complex with a low catalyst loading (1–2 mol%). This is the first example of the direct asymmetric synthesis of tertiary nitro alcohols derived from 2-acylpyridines, which were simple methyl ketones. The catalytic system is tolerant of air and moisture. Further investigations into other versions of asymmetric catalysis are in progress.

## Experimental

### General methods

Commercial reagents were used as purchased. High resolution mass spectra were recorded using a Bruker SolariX Fourier-transform ion cyclotron mass spectrometry (FT-ICR-MS) system. Nuclear magnetic resonance (NMR) spectra were recorded in the deuterated solvents [deuterated chloroform (CDCl_3_) or deuterated methanol (CD_3_OD)] as stated, using residual non-deuterated solvent as internal standard. The enantiomeric excess (ee) was determined using high-performance liquid chromatography (HPLC) analysis using the corresponding commercial chiral column as stated in the experimental procedures at 23 °C with an ultraviolet detector at 220 nm or 215 nm or 254 nm. Optical rotations were measured on a commercial polarimeter and are reported as follows: [*α*]^*T*^_D_ (*c* = g per 100 mL solvent).

### General procedure for catalytic asymmetric reaction

#### α-Keto esters

The mixture of CH_3_NO_2_ (0.2 mL), Ni–L1 (2 mol% or 1 mol%), 4 Å MS (30 mg) and *N*-methylmorpholine (10 mol%) was stirred in THF (0.8 mL) under an air atmosphere at 35 °C for 10 min followed by the addition of the α-keto ester (0.2 mmol). The stirring was continued for the reaction time given in Table 5 at 35 °C. The residue was purified using silica gel flash column chromatography (petroleum ether/ethyl acetate (EtOAc), 60 : 1–15 : 1) to give the products. The absolute configuration of 2ac was determined using X-ray crystallographic analysis. The absolute configuration of 2ab was assigned by comparison with the sign of optical rotation value found in the literature.^6*b*^ The absolute configuration of 2bc–2mc and 2aa was determined by analogy. The absolute configuration of 2nb was assigned by comparison with the sign of optical rotation values found in the literature.^6*a*,6*b*,6*e*,6*f*,6*i*^

#### 2-Acylpyridine *N*-oxides

The mixture of CH_3_NO_2_ (0.2 mL), Ni–L1 (2 mol%), 4 Å MS (30 mg), 2-acylpyridine *N*-oxides (0.2 mmol) and iPr_2_NH (5 mol%) was stirred in EtOH (0.8 mL) at the temperature specified in Table 6 (−30 °C or −40 °C) under an air atmosphere for the reaction time identified in Table 6. The residue was purified using silica gel flash column chromatography (petroleum ether/EtOAc, 10 : 1) to give the products. The absolute configuration of 4a–4d and 4f was assigned by comparison with the sign of optical rotation value found in the literature.^10^

#### 2-Acylpyridines

The mixture of CH_3_NO_2_ (0.2 mL), Ni–L1 (2 mol%), 4 Å MS (40 mg), 2-acylpyridine (0.2 mmol) and iPr_2_NEt (10 mol%) was stirred in THF (0.8 mL) at the temperature specified in Table 7 (−10 °C or −20 °C) under an air atmosphere for the reaction time indicated in Table 7. The residue was purified using silica gel flash column chromatography (petroleum ether/EtOAc, 10 : 1) to give the products. The absolute configuration of 6a was assigned by comparison with the sign of optical rotation value of its reduced product (7) found in the literature.^10^ The absolute configuration of 6b–6d and 6f was determined by analogy.

#### Methyl 2-hydroxy-3-nitro-2-phenyl propanoate (2aa)

Colorless oil, 38.3 mg, 85% yield, 83% ee; ^1^H-NMR (600 MHz, CDCl_3_) *δ* 7.59 (d, 2H, *J* = 7.1), 7.43–7.36 (m, 3H), 5.26 (d, 1H, *J* = 14.2), 4.68 (d, 1H, *J* = 14.2), 4.25 (s, 1H), 3.91 (s, 3H); [*α*]^25^_D_ = −4.8 [*c* 0.56, dichloromethane (CH_2_Cl_2_)] [lit.^6*i*^ [*α*]^25^_D_ = −15.6 (*c* 0.54, CH_2_Cl_2_) in 70% ee]; HPLC (CHIRALCEL OD-H column, hexane/2-propanol = 85/15, flow rate = 1.0 mL min^−1^, detection at 220 nm), retention time = 11.9 min (major) and = 9.1 min (minor).

#### Ethyl 2-hydroxy-3-nitro-2-phenyl propanoate (2ab)

Colorless oil, 38.2 mg, 80% yield, 89% ee; ^1^H-NMR (600 MHz, CDCl_3_) *δ* 7.61 (d, 2H, *J* = 7.2), 7.42–7.36 (m, 3H), 5.26 (d, 1H, *J* = 14.2), 4.68 (d, 1H, *J* = 14.2), 4.42–4.31 (m, 2H), 4.26 (s, 1H), 1.34 (t, 3H, *J* = 7.1); [*α*]^25^_D_ = −10.2 (*c* 0.54, CH_2_Cl_2_) [lit.^6*b*^ [*α*]^23^_D_ = −16.2 (*c* 1.13, CH_2_Cl_2_) in 86% ee]; HPLC (CHIRALCEL OD-H column, hexane/2-propanol = 90/10, flow rate = 1.0 mL min^−1^, detection at 220 nm), *t*_r_ = 12.9 min (major) and = 10.1 min (minor).

#### Isopropyl 2-hydroxy-3-nitro-2-phenyl propanoate (2ac)^6*i*^

White solid, 46.5 mg, 92% yield, 93% ee; ^1^H-NMR (500 MHz, CDCl_3_) *δ* 7.61 (d, 2H, *J* = 7.4), 7.42–7.35 (m, 3H), 5.24 (d, 1H, *J* = 14.2), 5.22–5.15 (m, 1H), 4.67 (d, 1H, *J* = 14.2), 4.23 (s, 1H), 1.35 (d, 3H, *J* = 6.3), 1.29 (d, 3H, *J* = 6.3); ^13^C-NMR (125 MHz, CDCl_3_) *δ* 171.2, 136.7, 129.1, 128.9, 125.3, 80.8, 76.0, 72.0, 21.6, 21.5. [*α*]^25^_D_ = −4.2 (*c* 0.70, CH_2_Cl_2_) [lit.^6*i*^ [*α*]^25^_D_ = −2.3 (*c* 1.07, CH_2_Cl_2_) in 62% ee]; HPLC (CHIRALCEL OD-H column, hexane/2-propanol = 90/10, flow rate = 1.0 mL min^−1^, detection at 220 nm), *t*_r_ = 9.9 min (major) and = 8.0 min (minor).

#### Isopropyl 2-hydroxy-2-(3-methyphenyl)-3-nitro propanoate (2bc)

Light yellow oil, 44.3 mg, 83% yield, 93% ee; ^1^H-NMR (500 MHz, CDCl_3_) *δ* 7.4 (s, 1H), 7.29 (d, 1H, *J* = 7.9), 7.20 (t, 1H, *J* = 7.7), 7.09 (d, 1H, *J* = 7.5), 5.15 (d, 1H, *J* = 14.2), 5.14–5.08 (m, 1H), 4.57 (d, 1H, *J* = 14.2), 4.16 (s, 1H), 2.29 (s, 3H), 1.27 (d, 3H, *J* = 6.3), 1.22 (d, 3H, *J* = 6.3); [*α*]^25^_D_ = −4.8 (*c* 0.88, CH_2_Cl_2_). ^13^C-NMR (125 MHz, CDCl_3_) *δ* 170.2, 137.7, 135.5, 128.8, 127.7, 124.9, 121.2, 79.8, 74.9, 70.8, 20.52, 20.46, 20.39. HRMS (ESI): *m*/*z* calc'd for C_13_H_17_NNaO_5_^+^ [M + Na]^+^: 290.0999, found: 290.0997. HPLC (CHIRALCEL OD-H column, hexane/2-propanol = 90/10, flow rate = 1.0 mL min^−1^, detection at 220 nm), *t*_r_ = 8.2 min (major) and = 6.5 min (minor).

#### Isopropyl 2-hydroxy-2-(4-methyphenyl)-3-nitro propanoate (2cc)

Light yellow oil, 41.4 mg, 78% yield, 91% ee; ^1^H-NMR (500 MHz, CDCl_3_) *δ* 7.48 (d, 2H, *J* = 8.2), 7.20 (d, 2H, *J* = 8.1), 5.24–5.14 (m, 2H), 4.64 (d, 1H, *J* = 14.2), 4.22 (s, 1H), 2.35 (s, 3H), 1.34 (d, 3H, *J* = 6.2), 1.29 (d, 3H, *J* = 6.3). ^13^C-NMR (125 MHz, CDCl_3_) *δ* 170.3, 138.0, 132.7, 128.5, 124.1, 79.8, 74.8, 70.8, 20.5, 20.4, 20.0. HRMS (ESI): *m*/*z* calc'd for C_13_H_17_NNaO_5_^+^ [M + Na]^+^: 290.0999, found: 290.1000. [*α*]^25^_D_ = −6.4 (*c* 0.82, CH_2_Cl_2_); HPLC (CHIRALCEL OD-H column, hexane/2-propanol = 90/10, flow rate = 1.0 mL min^−1^, detection at 220 nm), *t*_r_ = 9.2 min (major) and = 6.9 min (minor).

#### Isopropyl 2-hydroxy-2-(4-methoxyphenyl)-3-nitro propanoate (2dc)

White solid, 40.8 mg, 72% yield, 91% ee; ^1^H-NMR (500 MHz, CDCl_3_) *δ* 7.51 (d, 2H, *J* = 8.8), 6.91 (d, 2H, *J* = 8.8), 5.22–5.15 (m, 2H), 4.63 (d, 1H, *J* = 14.2), 4.20 (s, 1H), 3.81 (s, 3H), 1.34 (d, 3H, *J* = 6.3), 1.29 (d, 3H, *J* = 6.3). ^13^C-NMR (125 MHz, CDCl_3_) *δ* 170.3, 159.0, 127.5, 125.6, 113.1, 79.8, 74.6, 70.8, 54.3, 20.5, 20.4. HRMS (ESI): *m*/*z* calc'd for C_13_H_17_NNaO_6_^+^ [M + Na]^+^: 306.0948, found: 306.0945. [*α*]^25^_D_ = −11.4 (*c* 0.65, CH_2_Cl_2_); HPLC (CHIRALCEL OD-H column, hexane/2-propanol = 90/10, flow rate = 1.0 mL min^−1^, detection at 220 nm), *t*_r_ = 12.6 min (major) and *t*_r_ = 11.6 min (minor).

#### Isopropyl 2-(4-flurophenyl)-2-hydroxy-3-nitro propanoate (2ec)

Colorless oil, 48.5 mg, 90% yield, 94% ee; ^1^H-NMR (500 MHz, CDCl_3_) *δ* 7.62–7.58 (m, 2H), 7.08 (t, 2H, *J* = 8.6), 5.22–5.16 (m, 2H), 4.63 (d, 1H, *J* = 14.1), 4.27 (s, 1H), 1.34 (d, 3H, *J* = 6.3), 1.29 (d, 3H, *J* = 6.3). ^13^C-NMR (125 MHz, CDCl_3_) *δ* 169.9, 163.0 and 161.1 (^2^*J*_CF_ = 225.0, 1C), 131.3, 126.3, 126.2, 114.9, 114.7, 79.7, 74.5, 71.1, 20.5, 20.4. HRMS (ESI): *m*/*z* calc'd for C_12_H_14_FNNaO_5_^+^ [M + Na]^+^: 294.0748, found: 294.0748. [*α*]^25^_D_ = −4.2 (*c* 0.6, CH_2_Cl_2_); HPLC (CHIRALPAK IA column, hexane/2-propanol = 90/10, flow rate = 1.0 mL min^−1^, detection at 220 nm), *t*_r_ = 9.5 min (major) and *t*_r_ = 8.8 min (minor).

#### Isopropyl 2-hydroxy-2-(2-naphthyl)-3-nitro propanoate (2fc)

White solid, 60.6 mg, 99% yield, 93% ee; ^1^H-NMR (500 MHz, CDCl_3_) *δ* 8.14 (s, 1H), 7.89–7.84 (m, 3H), 7.67 (d, 1H, *J* = 8.7), 7.55–7.52 (m, 2H), 5.37 (d, 1H, *J* = 14.2), 5.26–5.17 (m, 1H), 4.75 (d, 1H, *J* = 14.2), 4.36 (brs, 1H), 1.37 (d, 3H, *J* = 6.3), 1.31 (d, 3H, *J* = 6.3). ^13^C-NMR (125 MHz, CDCl_3_) *δ* 169.1, 131.8, 131.3, 131.0, 126.7, 126.5, 125.6, 125.0, 124.8, 123.1, 120.4, 78.8, 74.1, 70.1, 19.6, 19.5. HRMS (ESI): *m*/*z* calc'd for C_16_H_17_NNaO_5_^+^ [M + Na]^+^: 326.0999, found: 326.1000. [*α*]^25^_D_ = −34.9 (*c* 0.53, CH_2_Cl_2_); HPLC (CHIRALPAK AS-H column, hexane/2-propanol = 90/10, flow rate = 1.0 mL min^−1^, detection at 220 nm), *t*_r_ = 9.5 min (major) and *t*_r_ = 8.8 min (minor).

#### Isopropyl 2-hydroxy-3-nitro-2-(4-propylphenyl) propanoate (2gc)

Light yellow oil, 46.7 mg, 79% yield, 89% ee; ^1^H-NMR (500 MHz, CDCl_3_) *δ* 7.49 (d, 2H, *J* = 8.2), 7.20 (d, 2H, *J* = 8.1), 5.22 (d, 1H, *J* = 14.2), 5.20–5.14 (m, 1H), 4.65 (d, 1H, *J* = 14.2), 4.20 (brs, 1H), 2.58 (t, 2H, *J* = 7.6), 1.68–1.59 (m, 2H), 1.34 (d, 3H, *J* = 6.3), 1.29 (d, 3H, *J* = 6.3), 0.93 (t, 3H, *J* = 7.3). ^13^C-NMR (125 MHz, CDCl_3_) *δ* 170.3, 142.7, 132.9, 127.9, 124.1, 79.8, 74.8, 70.8, 36.5, 23.3, 20.5, 20.4, 12.8. HRMS (ESI): *m*/*z* calc'd for C_15_ H_21_NNaO_5_^+^ [M + Na]^+^: 318.1312, found: 318.1311. [*α*]^25^_D_ = −7.8 (*c* 0.71, CH_2_Cl_2_); HPLC (CHIRALPAK IA column, hexane/2-propanol = 90/10, flow rate = 1.0 mL min^−1^, detection at 220 nm), *t*_r_ = 10.0 min (major) and *t*_r_ = 8.1 min (minor).

#### Isopropyl 2-(2-furyl)-2-hydroxy-3-nitropropanoate (2hc)

Light yellow oil, 45 mg, 90% yield, 78% ee; ^1^H-NMR (500 MHz, CDCl_3_) *δ* 7.41 (d, 1H, *J* = 0.6), 6.41–6.38 (m, 2H), 5.26–5.17 (m, 2H), 4.91 (d, 1H, *J* = 14.2), 4.21 (brs, 1H), 1.33 (d, 3H, *J* = 6.3), 1.27 (d, 3H, *J* = 6.3). ^13^C-NMR (125 MHz, CDCl_3_) *δ* 168.4, 148.3, 142.5, 109.8, 107.4, 77.2, 71.9, 71.2, 20.5, 20.3. HRMS (ESI): *m*/*z* calc'd for C_10_H_13_NNaO_6_^+^ [M + Na]^+^: 266.0635, found: 266.0634. [*α*]^25^_D_ = +3.1 (*c* 0.9, CH_2_Cl_2_); HPLC (CHIRALCEL OD-H column, hexane/2-propanol = 90/10, flow rate = 1.0 mL min^−1^, detection at 220 nm), *t*_r_ = 8.0 min (major) and *t*_r_ = 7.4 min (minor).

#### Isopropyl 2-(4-chlorophenyl)-2-hydroxy-3-nitro propanoate (2ic)

Light yellow oil, 57.4 mg, 99% yield, 94% ee; ^1^H-NMR (500 MHz, CDCl_3_) *δ* 7.55 (d, 2H, *J* = 8.6), 7.36 (d, 2H, *J* = 8.6), 5.22–5.13 (m, 2H), 4.63 (d, 1H, *J* = 14.1), 4.30 (brs, 1H), 1.34 (d, 3H, *J* = 6.3), 1.28 (d, 3H, *J* = 6.3). ^13^C-NMR (125 MHz, CDCl_3_) *δ* 169.8, 134.2, 134.1, 128.0, 125.8, 79.5, 74.6, 71.2, 20.5, 20.4. HRMS (ESI): *m*/*z* calc'd for C_12_H_14_ClNNaO_5_^+^ [M + Na]^+^: 310.0453, found: 310.0449. [*α*]^25^_D_ = −8.7 (*c* 1.4, CH_2_Cl_2_); HPLC (CHIRALCEL OD-H column, hexane/2-propanol = 90/10, flow rate = 1.0 mL min^−1^, detection at 220 nm), *t*_r_ = 9.2 min (major) and *t*_r_ = 7.7 min (minor).

#### Isopropyl 2-(3-chlorophenyl)-2-hydroxy-3-nitro propanoate (2jc)

Light yellow oil, 57.4 mg, 99% yield, 91% ee; ^1^H-NMR (500 MHz, CDCl_3_) *δ* 7.65 (s, 1H), 7.50–7.49 (m, 1H), 7.37–7.26 (m, 2H), 5.23–5.18 (m, 2H), 4.64 (d, 1H, *J* = 14.2), 4.27 (s, 1H), 1.35 (d, 3H, *J* = 6.3), 1.31 (d, 3H, *J* = 6.3). ^13^C-NMR (125 MHz, CDCl_3_) *δ* 169.6, 137.5, 134.0, 129.1, 128.3, 124.8, 122.4, 79.5, 74.5, 71.4, 20.5, 20.4. HRMS (ESI): *m*/*z* calc'd for C_12_H_14_ClNNaO_5_^+^ [M + Na]^+^: 310.0453, found: 310.0451. [*α*]^25^_D_ = −8.3 (*c* 1.8, CH_2_Cl_2_); HPLC (CHIRALPAK IA column, hexane/2-propanol = 90/10, flow rate = 1.0 mL min^−1^, detection at 220 nm), *t*_r_ = 8.5 min (major) and *t*_r_ = 8.0 min (minor).

#### Isopropyl 2-(4-bromophenyl)-2-hydroxy-3-nitro propanoate (2kc)

Colorless oil, 57.2 mg, 99% yield, 94% ee; ^1^H-NMR (500 MHz, CDCl_3_) *δ* 7.51 (q, 4H, *J* = 8.9), 5.21–5.13 (m, 2H), 4.63 (d, 1H, *J* = 14.1), 4.28 (brs, 1H), 1.34 (d, 3H, *J* = 6.3), 1.28 (d, 3H, *J* = 6.3). ^13^C-NMR (125 MHz, CDCl_3_) *δ* 169.7, 134.6, 131.0, 126.1, 122.5, 79.5, 74.6, 71.3, 20.5, 20.4. HRMS (ESI): *m*/*z* calc'd for C_12_H_14_BrNNaO_5_^+^ [M + Na]^+^: 353.9948, found: 353.9945. [*α*]^25^_D_ = −8.1 (*c* 2.3, CH_2_Cl_2_); HPLC (CHIRALCEL OD-H column, hexane/2-propanol = 90/10, flow rate = 1.0 mL min^−1^, detection at 220 nm), *t*_r_ = 12.7 min (major) and *t*_r_ = 9.0 min (minor).

#### Isopropyl 2-(3,4-dichlorophenyl)-2-hydroxy-3-nitro propanoate (2lc)

Light yellow oil, 64.2 mg, 99% yield, 91% ee; ^1^H-NMR (500 MHz, CDCl_3_) *δ* 7.76 (s, 1H), 7.49–7.44 (m, 2H), 5.22–5.15 (m, 2H), 4.62 (d, 1H, *J* = 14.2), 4.32 (s, 1H), 1.35 (d, 3H, *J* = 6.3), 1.31 (d, 3H, *J* = 6.3). ^13^C-NMR (125 MHz, CDCl_3_) *δ* 169.3, 135.6, 132.6, 132.3, 129.8, 126.7, 123.7, 79.4, 74.2, 71.6, 20.5, 20.4. HRMS (ESI): *m*/*z* calc'd for C_12_H_13_Cl_2_NNaO_5_^+^ [M + Na]^+^: 344.0063, found: 344.0066. [*α*]^25^_D_ = −12.5 (*c* 0.21, CH_2_Cl_2_); HPLC (CHIRALPAK IA column, hexane/2-propanol = 90/10, flow rate = 1.0 mL min^−1^, detection at 220 nm), *t*_r_ = 8.6 min (major) and *t*_r_ = 7.7 min (minor).

#### Isopropyl 2-hydroxy-3-nitro-2-(4-trifluoromethyl-phenyl)propanoate (2mc)

Light yellow solid, 70.4 mg, 99% yield, 91% ee; ^1^H-NMR (500 MHz, CDCl_3_) *δ* 7.77 (d, 2H, *J* = 8.2), 7.67 (d, 2H, *J* = 8.3), 5.26–5.16 (m, 2H), 4.65 (d, 1H, *J* = 14.2), 4.34 (brs, 1H), 1.36 (d, 3H, *J* = 6.3), 1.30 (d, 3H, *J* = 6.3). ^13^C-NMR (125 MHz, CDCl_3_) *δ* 169.5, 139.5, 130.5, 130.3, 124.9, 124.9–124.7 (q, ^4^*J*_CF_ = 3.8, 1C), 79.5, 74.7, 71.5, 20.5, 20.4. HRMS (ESI): *m*/*z* calc'd for C_13_H_14_F_3_NNaO_5_^+^ [M + Na]^+^: 344.0716, found: 344.0715. [*α*]^25^_D_ = −4.1 (*c* 0.97, CH_2_Cl_2_); HPLC (CHIRALPAK IA column, hexane/2-propanol = 90/10, flow rate = 1.0 mL min^−1^, detection at 220 nm), *t*_r_ = 10.6 min (major) and *t*_r_ = 9.8 min (minor).

#### Ethyl 2-hydroxy-2-methyl-3-nitropropanoate (2nb)

Light yellow oil, 32.2 mg, 91% yield, 80% ee; ^1^H-NMR (500 MHz, CDCl_3_) *δ* 4.83 (d, 1H, *J* = 13.8), 4.55 (d, 1H, *J* = 13.8), 4.36–4.28 (m, 2H), 3.81 (s, 1H), 1.44 (s, 3H), 1.31 (t, 3H, *J* = 6.8). ^13^C-NMR (125 MHz, CDCl_3_) *δ* 171.6, 79.1, 70.6, 61.2, 22.0, 12.1. [*α*]^25^_D_ = +9.2 (*c* 0.6, CH_2_Cl_2_) [lit.^6*b*^ [*α*]^23^_D_ = +10.2 (*c* 1.19, CH_2_Cl_2_) in 92% ee]; HPLC (CHIRALPAK AS-H column, hexane/2-propanol = 95/5, flow rate = 1.0 mL min^−1^, detection at 215 nm), *t*_r_ = 21.3 min (major) and *t*_r_ = 17.7 min (minor).

#### 1-Methyl-2-nitro-1-(1-oxido-2-pyridinyl) ethanol (4a)

Brown oil, 36.4 mg, 92% yield, 86% ee; ^1^H-NMR (500 MHz, CDCl_3_) *δ* 8.23 (d, 1H, *J* = 6.3), 7.78 (s, 1H), 7.46–7.39 (m, 2H), 7.36–7.32 (m, 1H), 5.30 (d, 1H, *J* = 11.1), 4.85 (d, 1H, *J* = 11.1), 1.77 (s, 3H); [*α*]^20^_D_ = +41.3 (*c* 0.45, MeOH) [lit.^10^ [*α*]^20^_D_ = +48 (*c* 0.9, MeOH) in 86% ee]; HPLC (CHIRALPAK AD-H column, hexane/2-propanol = 75/25, flow rate = 1.0 mL min^−1^, detection at 220 nm), *t*_r_ = 10.5 min (major) and *t*_r_ = 25.0 min (minor).

#### 1-Methyl-2-nitro-1-(4-methyl-1-oxido-2-pyridinyl) ethanol (4b)

Brown solid, 39.9 mg, 94% yield, 84% ee; ^1^H-NMR (500 MHz, CDCl_3_) *δ* 8.22 (s, 1H), 8.14 (d, 1H, *J* = 6.6), 7.20–7.15 (m, 2H), 5.43 (d, 1H, *J* = 11.0), 4.74 (d, 1H, *J* = 11.0), 2.40 (s, 3H), 1.79 (s, 3H); [*α*]^20^_D_ = +34.0 (*c* 0.61, MeOH) [lit.^10^ [*α*]^20^_D_ = +41 (*c* 0.9, MeOH) in 84% ee]; HPLC (CHIRALPAK AD-H column, hexane/2-propanol = 75/25, flow rate = 1.0 mL min^−1^, detection at 254 nm), *t*_r_ = 7.2 min (major) and *t*_r_ = 25.8 min (minor).

#### 1-Methyl-2-nitro-1-(5-methyl-1-oxido-2-pyridinyl) ethanol (4c)

Brown oil, 36.6 mg, 86% yield, 85% ee; ^1^H-NMR (500 MHz, CDCl_3_) *δ* 8.11 (s, 1H), 8.00 (s, 1H), 7.29 (d, 1H, *J* = 8.2), 7.26–7.23 (m, 1H), 5.40 (d, 1H, *J* = 11.0), 4.73 (d, 1H, *J* = 11.0), 2.35 (s, 3H), 1.78 (s, 3H); [*α*]^20^_D_ = +56.7 (*c* 0.52, MeOH) [lit.^10^ [*α*]^20^_D_ = +60 (*c* 0.6, MeOH) in 81% ee]; HPLC (CHIRALPAK AD-H column, hexane/2-propanol = 75/25, flow rate = 1.0 mL min^−1^, detection at 254 nm), *t*_r_ = 12.5 min (major) and *t*_r_ = 18.1 min (minor).

#### 1-Methyl-2-nitro-1-(6-methyl-1-oxido-2-pyridinyl) ethanol (4d)

White solid, 38.2 mg, 90% yield, 84% ee; ^1^H-NMR (500 MHz, CDCl_3_) *δ* 8.25 (s, 1H), 7.36–7.27 (m, 3H), 5.39 (d, 1H, *J* = 10.9), 4.74 (d, 1H, *J* = 10.9), 2.54 (s, 3H), 1.79 (s, 3H); [*α*]^20^_D_ = +70.0 (*c* 0.56, MeOH) [lit.^10^ [*α*]^20^_D_ = +109 (*c* 0.9, MeOH) in 55% ee]; HPLC (CHIRALPAK AD-H column, hexane/2-propanol = 80/20, flow rate = 1.0 mL min^−1^, detection at 254 nm), *t*_r_ = 8.2 min (major) and *t*_r_ = 11.8 min (minor).

#### 1-Methyl-2-nitro-1-(5-bromo-1-oxido-2-pyridinyl) ethanol (4f)

White solid, 44.9 mg, 81% yield, 73% ee; ^1^H-NMR (500 MHz, CDCl_3_) *δ* 8.41 (d, 1H, *J* = 1.9), 7.56 (dd, 1H, *J* = 8.7, 1.7), 7.31 (d, 1H, *J* = 10.8), 7.19 (s, 1H), 5.38 (d, 1H, *J* = 14.3), 4.79 (d, 1H, *J* = 14.3), 1.79 (s, 3H); [*α*]^20^_D_ = +59.0 (*c* 0.6, MeOH) [lit.^10^ [*α*]^20^_D_ = +74 (*c* 0.9, MeOH) in 89% ee]; HPLC (CHIRALPAK AD-H column, hexane/2-propanol = 80/20, flow rate = 1.0 mL min^−1^, detection at 254 nm), *t*_r_ = 9.9 min (major) and *t*_r_ = 11.1 min (minor).

#### 1-Nitromethyl-1-(1-oxido-2-pyridinyl)propan-1-ol (4g)

Brown oil, 25.7 mg, 61% yield, 9% ee; ^1^H-NMR (400 MHz, CDCl_3_) *δ* 8.26 (d, 1H, *J* = 6.4), 7.58 (s, 1H), 7.44–7.39 (m, 2H), 7.36–7.31 (m, 1H), 5.25 (d, 1H, *J* = 11.4), 5.02 (d, 1H, *J* = 11.4), 2.31–2.21 (m, 1H), 2.11–2.03 (m, 1H), 1.05 (t, 3H, *J* = 7.3).

#### 1-Nitro-2-(pyridin-2-yl)propan-2-ol (6a)

Brown oil, 19.6 mg, 54% yield, 85% ee; [*α*]^20^_D_ = +35.2 (*c* 0.35, MeOH); ^1^H-NMR (400 MHz, CDCl_3_) *δ* 8.51 (d, 1H, *J* = 4.5), 7.76 (t, 1H, *J* = 7.1), 7.54 (d, 1H, *J* = 8.0), 7.26–7.23 (m, 1H), 4.99 (s, 1H), 4.95 (d, 1H, *J* = 12.3), 4.70 (d, 1H, *J* = 12.3), 1.62 (s, 3H); ^13^C-NMR (100 MHz, CDCl_3_) *δ* 160.9(C), 148.2(CH), 137.4(CH), 122.9(CH), 119.4(CH), 83.7(CH_2_), 73.6(C), 26.6(CH_3_). HRMS (ESI): *m*/*z* calc'd for C_8_H_10_N_2_NaO_3_^+^ [M + Na]^+^: 205.0584, found: 205.0588. HPLC (CHIRALPAK IA column, hexane/2-propanol = 90/10, flow rate = 0.8 mL min^−1^, detection at 254 nm), *t*_r_ = 12.1 min (major) and *t*_r_ = 13.5 min (minor).

#### 1-Methyl-2-nitro-1-(4-methyl-2-pyridinyl)ethanol (6b)

Brown oil, 23.7 mg, 60% yield, 72% ee; [*α*]^20^_D_ = +28.2 (*c* 0.33, MeOH); ^1^H-NMR (400 MHz, CDCl_3_) *δ* 8.35 (d, 1H, *J* = 5.0), 7.34 (s, 1H), 7.05 (d, 1H, *J* = 4.9), 5.07 (s, 1H), 4.91 (d, 1H, *J* = 12.2), 4.69 (d, 1H, *J* = 12.2), 2.38 (s, 3H), 1.60 (s, 3H); ^13^C-NMR (100 MHz, CDCl_3_) *δ* 160.7(C), 148.8(CH), 147.8(C), 123.9(CH), 120.1(CH), 83.8(CH_2_), 73.5(C), 26.5(CH_3_), 21.2(CH_3_). HRMS (ESI): *m*/*z* calc'd for C_9_H_13_N_2_O_3_^+^ [M + H]^+^: 197.0921, found: 197.0925. HPLC (CHIRALPAK AS-H column, hexane/2-propanol = 90/10, flow rate = 1.0 mL min^−1^, detection at 254 nm), *t*_r_ = 14.9 min (major) and *t*_r_ = 12.7 min (minor).

#### 1-Methyl-2-nitro-1-(5-methyl-2-pyridinyl)ethanol (6c)

Brown oil, 18.8 mg, 48% yield, 36% ee; [*α*]^20^_D_ = −10.6 (*c* 0.16, MeOH); ^1^H-NMR (400 MHz, CDCl_3_) *δ* 8.32 (s, 1H), 7.55 (d, 1H, *J* = 8.0), 7.41 (d, 1H, *J* = 8.1), 5.08 (s, 1H), 4.89 (d, 1H, *J* = 12.1), 4.68 (d, 1H, *J* = 12.1), 2.32 (s, 3H), 1.59 (s, 3H); ^13^C-NMR (100 MHz, CDCl_3_) *δ* 157.9(C), 148.4(CH), 137.9(CH), 132.5(C), 118.9(CH), 83.9(CH_2_), 73.4(C), 26.5(CH_3_), 18.0(CH_3_). HRMS (ESI): *m*/*z* calc'd for C_9_H_13_N_2_O_3_^+^ [M + H]^+^: 197.0921, found: 197.0926. HPLC (CHIRALPAK AS-H column, hexane/2-propanol = 90/10, flow rate = 1.0 mL min^−1^, detection at 254 nm), *t*_r_ = 16.7 min (major) and *t*_r_ = 14.8 min (minor).

#### 1-Methyl-2-nitro-1-(6-methyl-2-pyridinyl)ethanol (6d)

Brown oil, 15.3 mg, 39% yield, 71% ee; [*α*]^20^_D_ = −30.4 (*c* 0.24, MeOH); ^1^H-NMR (400 MHz, CDCl_3_) *δ* 7.62 (t, 1H, *J* = 7.7), 7.26 (d, 1H, *J* = 7.9), 7.08 (d, 1H, *J* = 7.6), 5.41 (s, 1H), 4.83 (d, 1H, *J* = 11.8), 4.66 (d, 1H, *J* = 11.8), 2.51 (s, 3H), 1.60 (s, 3H); ^13^C-NMR (100 MHz, CDCl_3_) *δ* 159.6(C), 157.1(C), 137.6(CH), 122.5(CH), 116.2(CH), 84.2(CH_2_), 73.1(C), 26.4(CH_3_), 24.2(CH_3_). HRMS (ESI): *m*/*z* calc'd for C_9_H_12_N_2_NaO_3_^+^ [M + Na]^+^: 219.0740, found: 219.0744. HPLC (CHIRALPAK AD-H column, hexane/2-propanol = 95/05, flow rate = 0.8 mL min^−1^, detection at 254 nm), *t*_r_ = 10.5 min (major) and *t*_r_ = 11.2 min (minor).

#### 1-Methyl-2-nitro-1-(5-bromo-2-pyridinyl)ethanol (6f)

Brown oil, 26.6 mg, 51% yield, 86% ee; [*α*]^20^_D_ = −37.3 (*c* 0.45, MeOH); ^1^H-NMR (400 MHz, CDCl_3_) *δ* 8.55 (d, 1H, *J* = 1.8), 7.87 (dd, 1H, *J* = 8.5, 2.2), 7.51 (d, 1H, *J* = 8.4), 5.01 (d, 1H, *J* = 12.8), 4.70 (d, 1H, *J* = 12.8), 4.56 (s, 1H), 1.57 (s, 3H); ^13^C-NMR (100 MHz, CDCl_3_) *δ* 160.1(C), 149.4(CH), 139.9(CH), 121.0(CH), 119.9(C), 83.0(CH_2_), 73.9(C), 26.8(CH_3_). HRMS (ESI): *m*/*z* calc'd for C_8_H_9_BrN_2_NaO_3_^+^ [M + Na]^+^: 282.9689, found: 282.9697. HPLC (CHIRALPAK AS-H column, hexane/2-propanol = 90/10, flow rate = 1.0 mL min^−1^, detection at 254 nm), *t*_r_ = 14.8 min (major) and *t*_r_ = 13.1 min (minor).

#### 1-Nitromethyl-1-(2-pyridinyl)propan-1-ol (6g)

Brown oil, 25.5 mg, 60% yield, 2% ee; ^1^H-NMR (400 MHz, CDCl_3_) *δ* 8.49 (d, 1H, *J* = 4.6), 7.73 (t, 1H, *J* = 7.7), 7.47 (d, 1H, *J* = 8.0), 7.24–7.20 (m, 1H), 5.14 (s, 1H), 4.94 (d, 1H, *J* = 12.2), 4.71 (d, 1H, *J* = 12.2), 1.94–1.87 (m, 2H), 0.74 (t, 3H, *J* = 7.4). ^13^C-NMR (100 MHz, CDCl_3_) *δ* 159.4(C), 148.0(CH), 137.2(CH), 122.8(CH), 120.0(CH), 83.3(CH_2_), 76.0(C), 32.1(CH_2_), 7.1(CH_3_). HRMS (ESI): *m*/*z* calc'd for C_9_H_13_N_2_O_3_^+^ [M + H]^+^: 197.0921, found: 197.0925.

#### 1-Amino-2-(pyridin-2-yl) propan-2-ol (7)

To a solution of 6a (18.2 mg, 0.10 mmol) in MeOH (10 mL) was added 5% palladium/carbon (20 mg) and the mixture was stirred vigorously at rt under an hydrogen atmosphere for 16 h. The catalyst was removed using filtration through a short pad of Celite, the filtrate was purified using silica gel flash column chromatography (EtOAc/MeOH, 6 : 1) to give 12.3 mg (81%) of compound 7. [*α*]^20^_D_ = +26.7 (*c* 0.21, MeOH) [lit.^10^ [*α*]^20^_D_ = +33 (*c* 0.8, MeOH) in 86% ee]; ^1^H-NMR (400 MHz, CD_3_OD) *δ* 8.50 (d, 1H, *J* = 4.4), 7.81 (td, 1H, *J* = 7.7, 1.4), 7.68 (d, 1H, *J* = 8.0), 7.28–7.24 (m, 1H), 3.07 (d, 1H, *J* = 13.2), 2.89 (d, 1H, *J* = 13.2), 1.48 (s, 3H); ^13^C-NMR (100 MHz, CD_3_OD) *δ* 164.5(C), 147.9(CH), 137.1(CH), 121.9(CH), 120.0(CH), 74.6(C), 51.2(CH_2_), 25.5(CH_3_).

## Conflicts of interest

There are no conflicts to declare.

## Supplementary Material

RA-008-C8RA00552D-s001

RA-008-C8RA00552D-s002
