# Supplementary material for: Catalytic enantioselective Henry reaction of α-keto esters, 2-acylpyridines and 2-acylpyridine N-oxides
Source: RSC Adv. 2018 Mar 5;8(17):9414–22. doi: 10.1039/c8ra00552d (PMC9078658; doi:10.1039/c8ra00552d)
Supplement: RA-008-C8RA00552D-s001 [file RA-008-C8RA00552D-s001.pdf]

Electronic Supplementary Material (ESI) for RSC Advances.
This journal is © The Royal Society of Chemistry 2018

Supporting Information for:

Catalytic Enantioselective Henry Reaction of α-Keto Esters, 2-Acylpyridines and 2-Acylpyridine *N*-Oxides

Feilong He, Guanghui Chen, Junxia Yang, Guojuan Liang, Ping Deng, Yan Xiong, and Hui Zhou*

hzhou@cqmu.edu.cn

School of Pharmaceutical Science, Chongqing Medical University, and School of Chemistry and Chemical Engineering, Chongqing University, Chongqing, China

Contents:

1. ESI-MS analysis of the mixture of Ni-**L1** and **3a**........2
2. Selected HPLC chromatograms for *ee* determination 3
3. Optimizations of the Henry reaction of 2-Acylpyridines and 2-Acylpyridine *N*-Oxides........28
4. Selected NMR spectra........30
5. X-ray crystal structure of **2ac** 80

1. ESI-MS analysis of the mixture of Ni-L1 and 3a

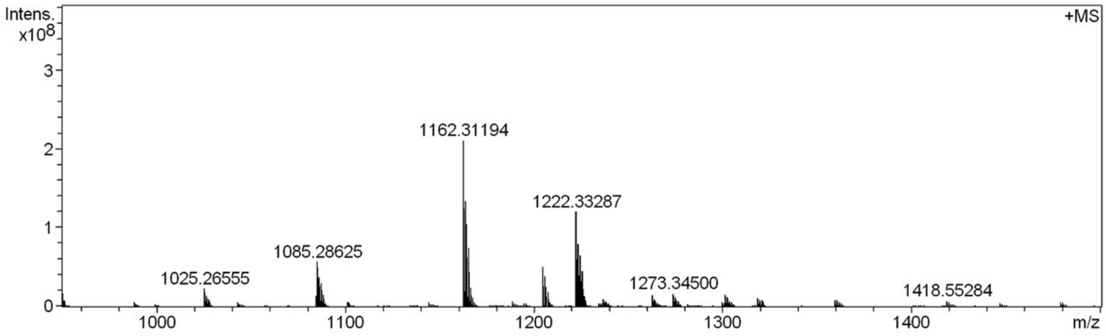

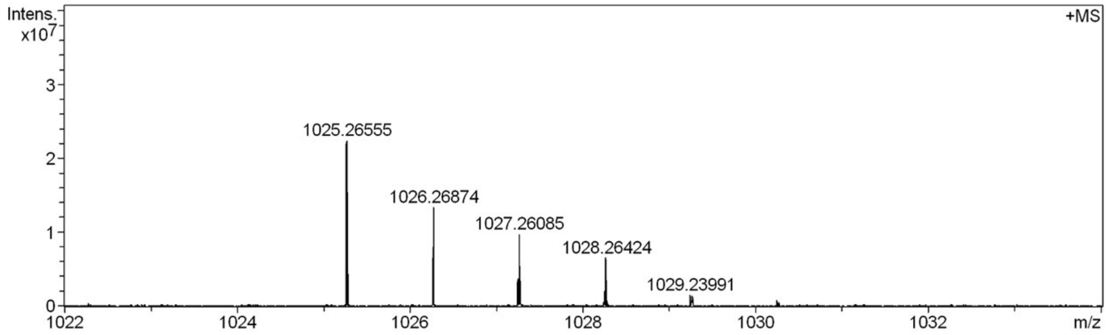

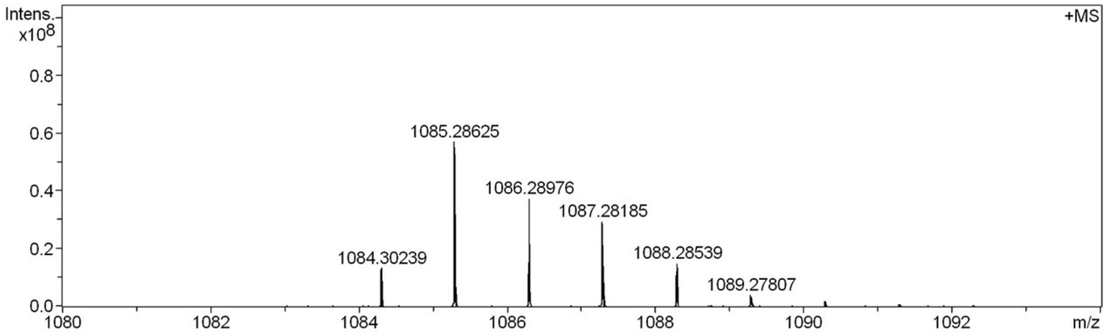

2. Selected HPLC chromatograms for *ee* determination

(1) **2ac**

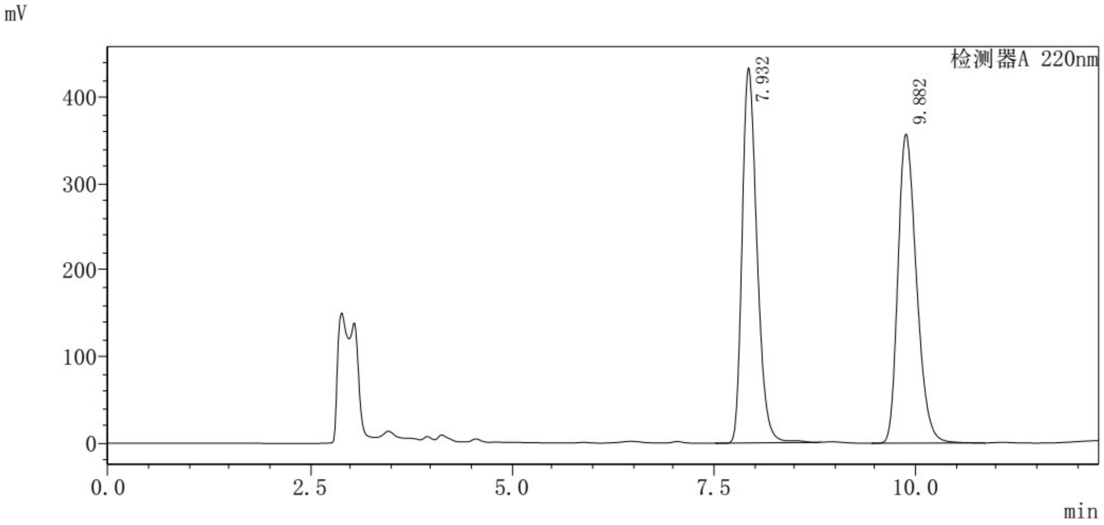

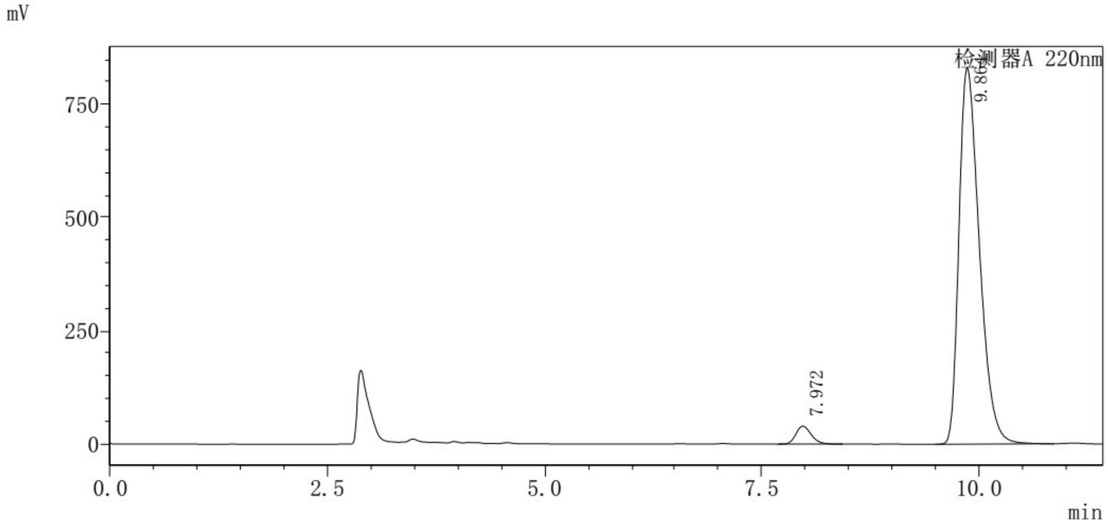

Peak#	Retention Time	Height	Area	Area%
1	7.972	39759	488984	3.480
2	9.864	831358	13562248	96.520
Total		871117	14051233	100.000

(2) **2bc**

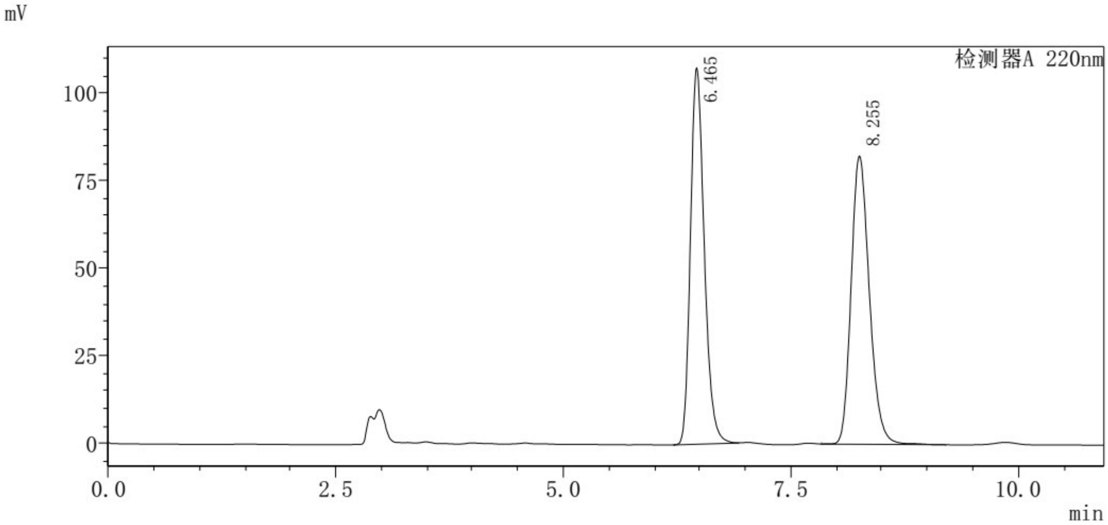

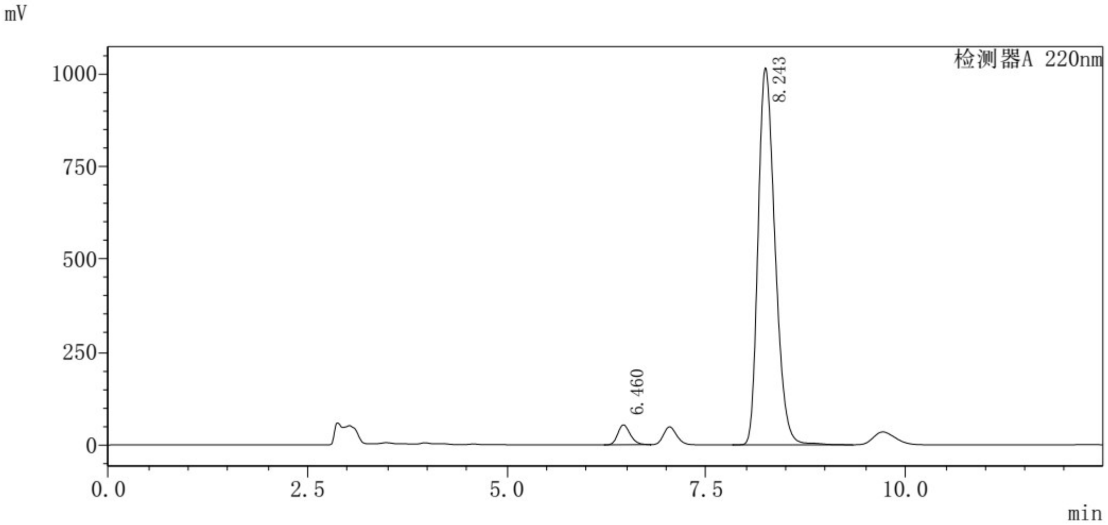

Peak#	Retention Time	Height	Area	Area%
1	6.460	53554	582256	3.742
2	8.243	1014285	14979316	96.258
Total		1067839	15561572	100.000

(3) **2cc**

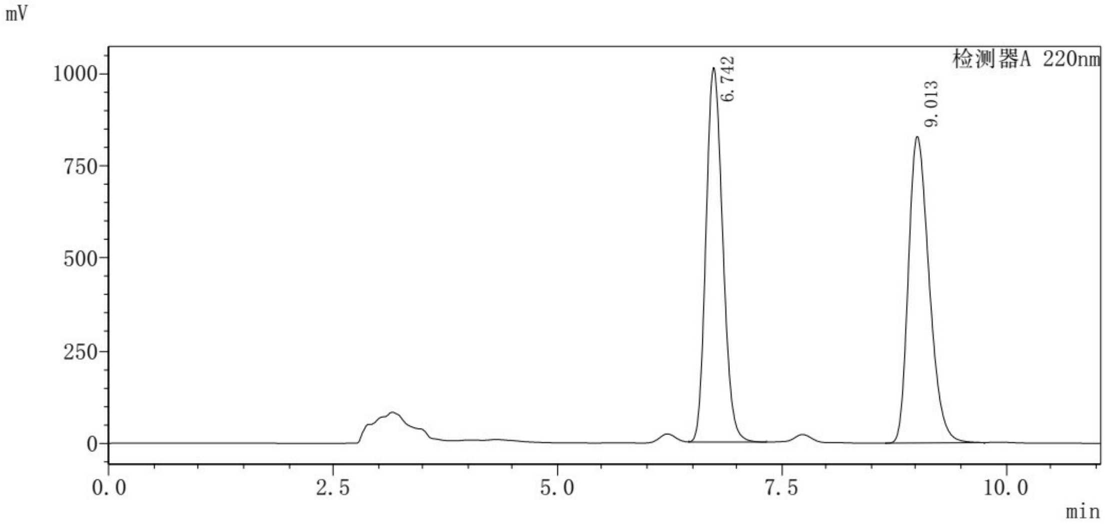

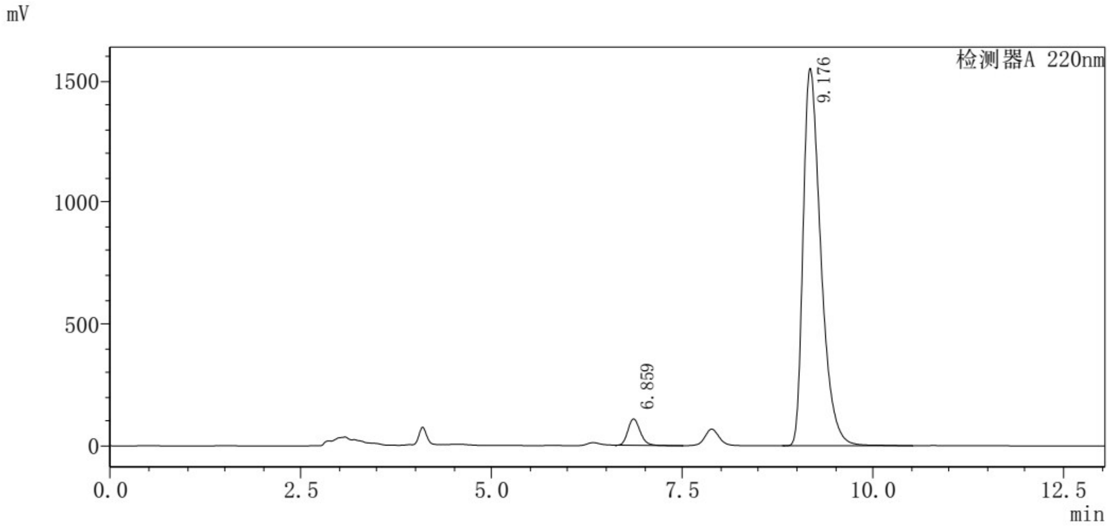

Peak#	Retention Time	Height	Area	Area%
1	6.859	108670	1209803	4.608
2	9.176	1551565	25044603	95.392
Total		1660234	26254405	100.000

(4) **2dc**

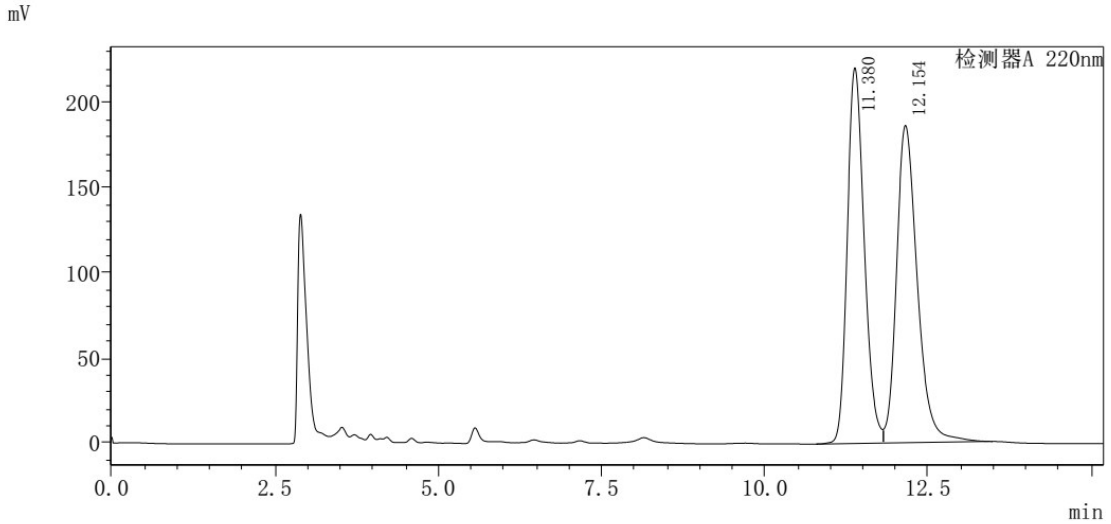

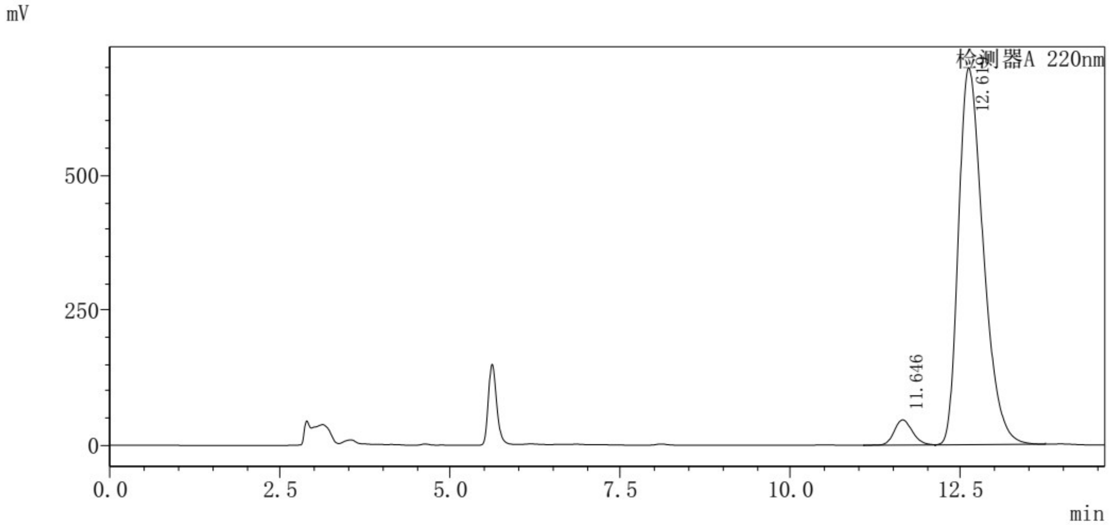

Peak#	Retention Time	Height	Area	Area%
1	11.646	46654	866835	4.662
2	12.619	698252	17727028	95.338
Total		744907	18593864	100.000

(5) **2ec**

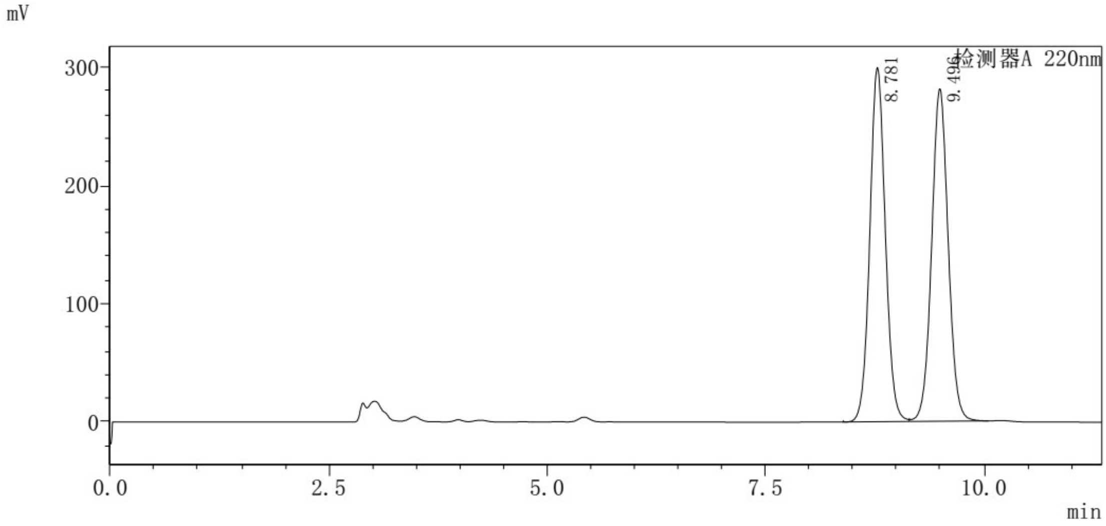

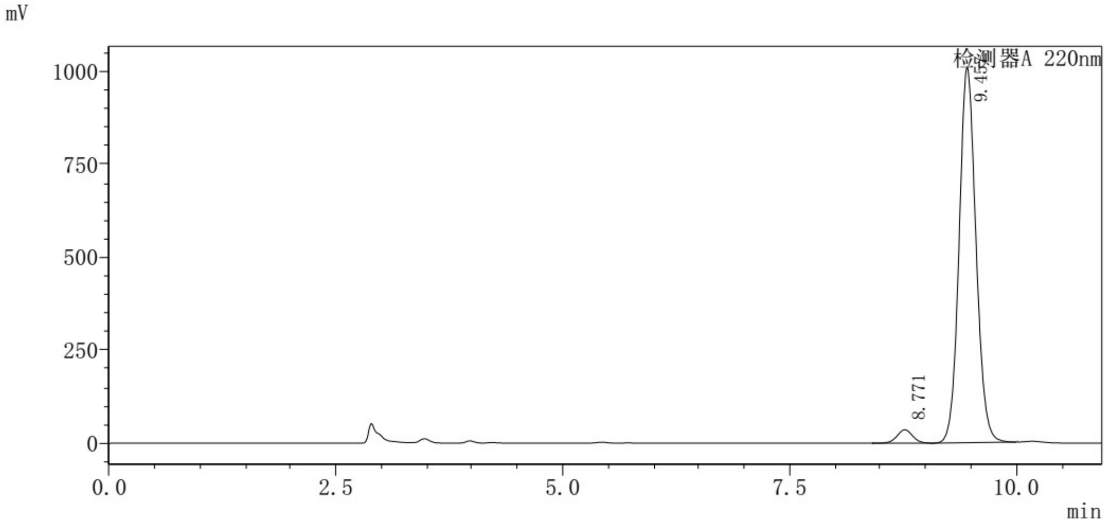

Peak#	Retention Time	Height	Area	Area%
1	8.771	35957	429708	3.178
2	9.457	1009238	13089628	96.822
Total		1045195	13519336	100.000

(6) **2fc**

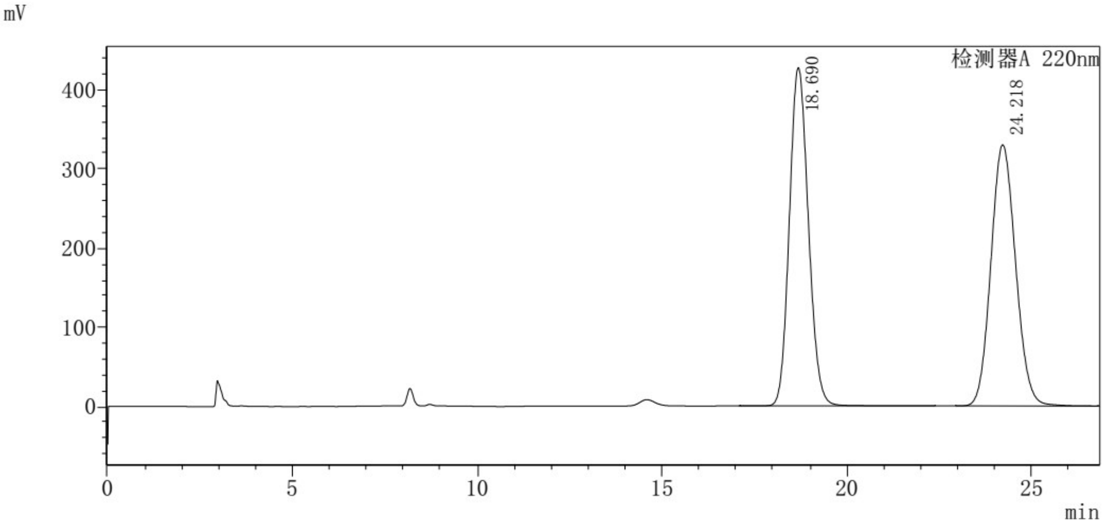

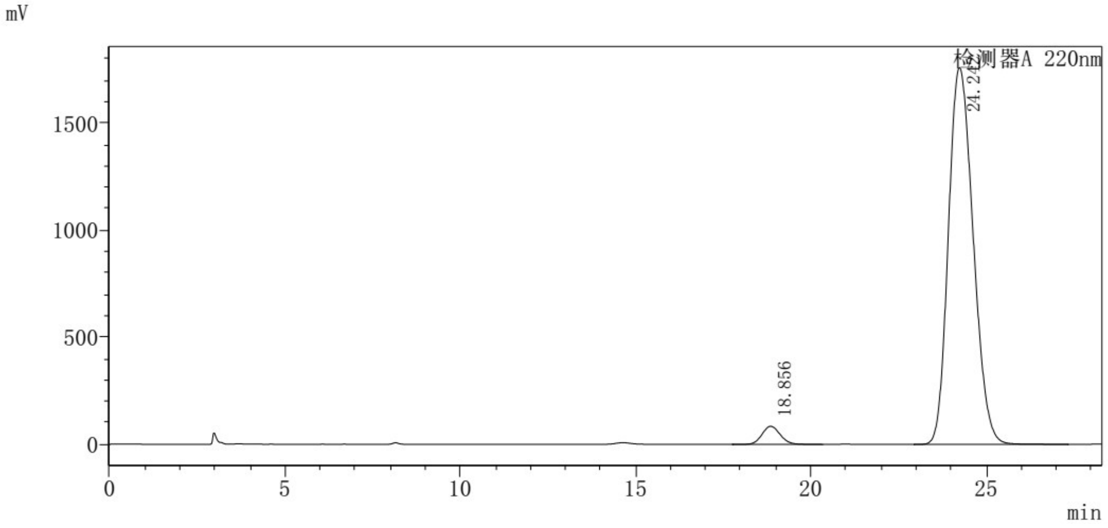

Peak#	Retention Time	Height	Area	Area%
1	18.856	84528	3028399	3.385
2	24.242	1760722	86428756	96.615
Total		1845250	89457155	100.000

(7) **2gc**

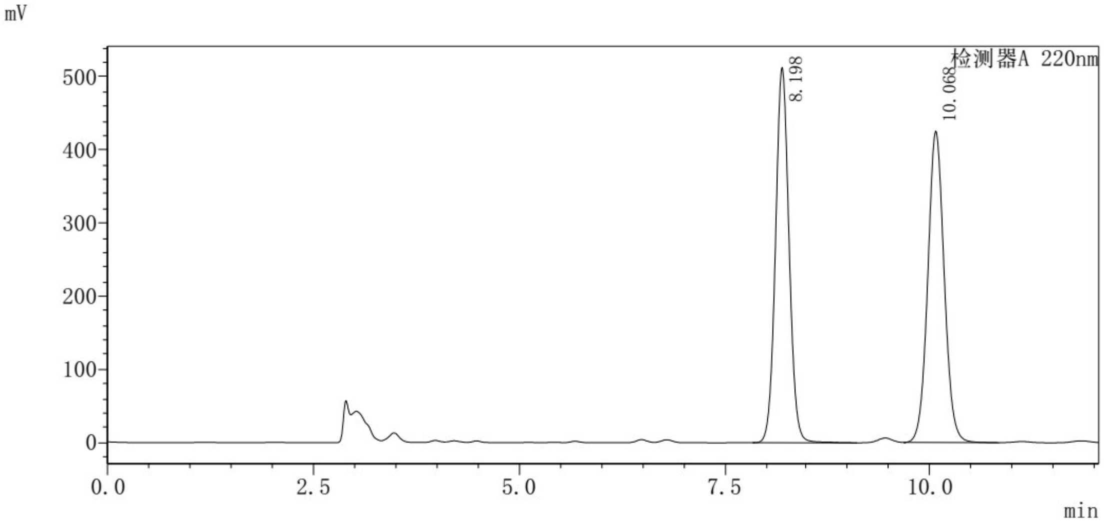

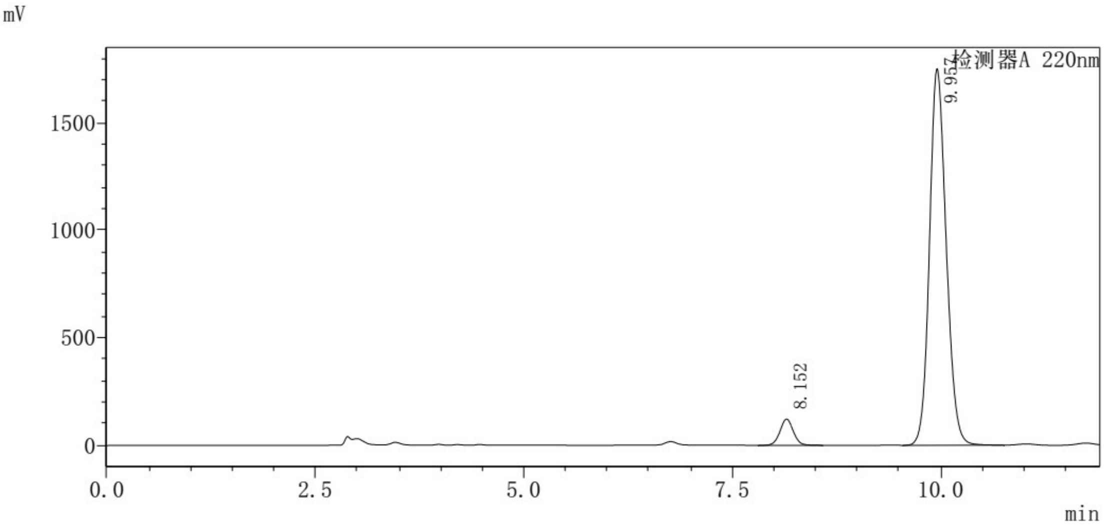

Peak#	Retention Time	Height	Area	Area%
1	8.152	122087	1361953	5.285
2	9.957	1750085	24408087	94.715
Total		1872172	25770039	100.000

(8) **2hc**

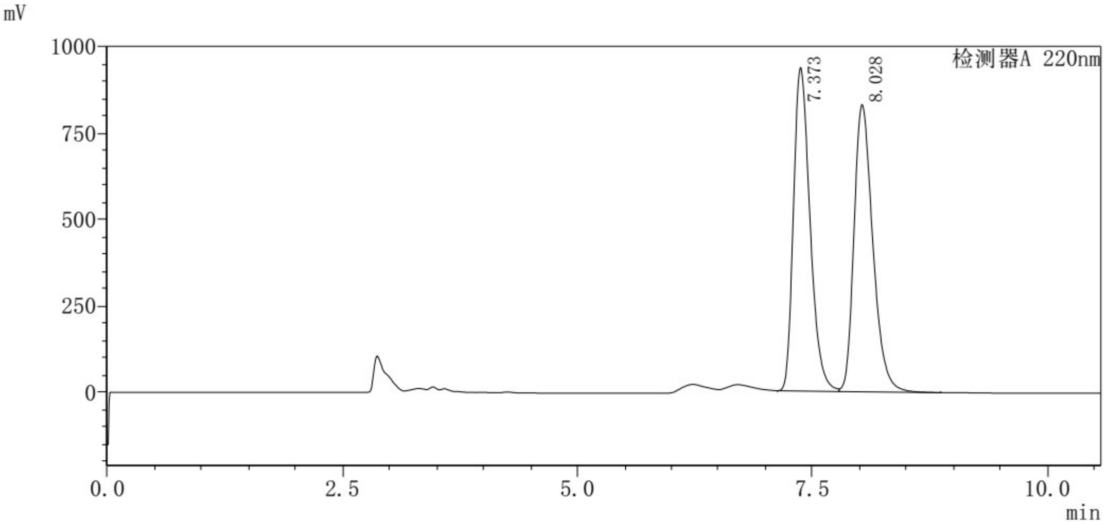

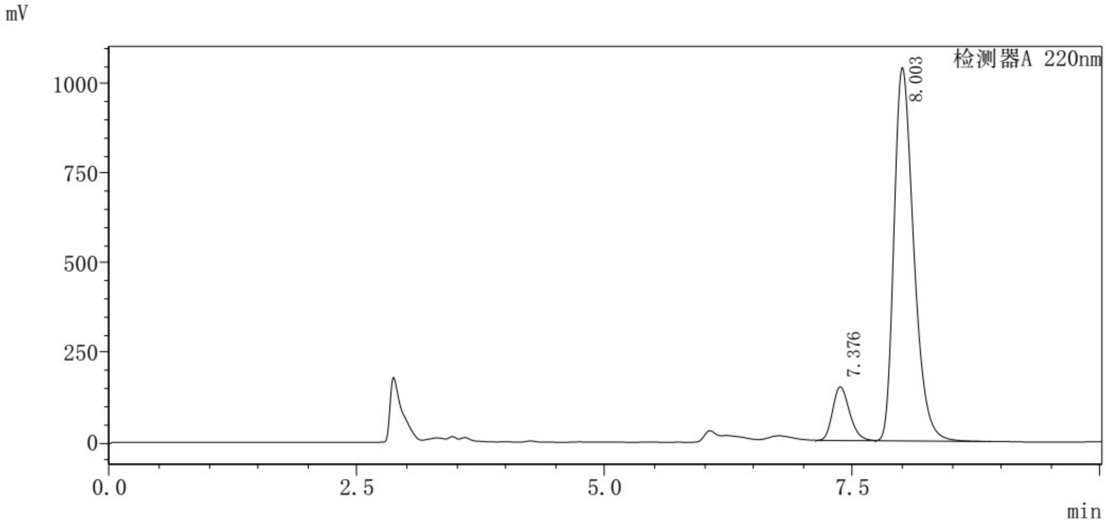

Peak#	Retention Time	Height	Area	Area%
1	7.376	149965	1779194	10.968
2	8.003	1042397	14442758	89.032
Total		1192362	16221952	100.000

(9) **2ic**

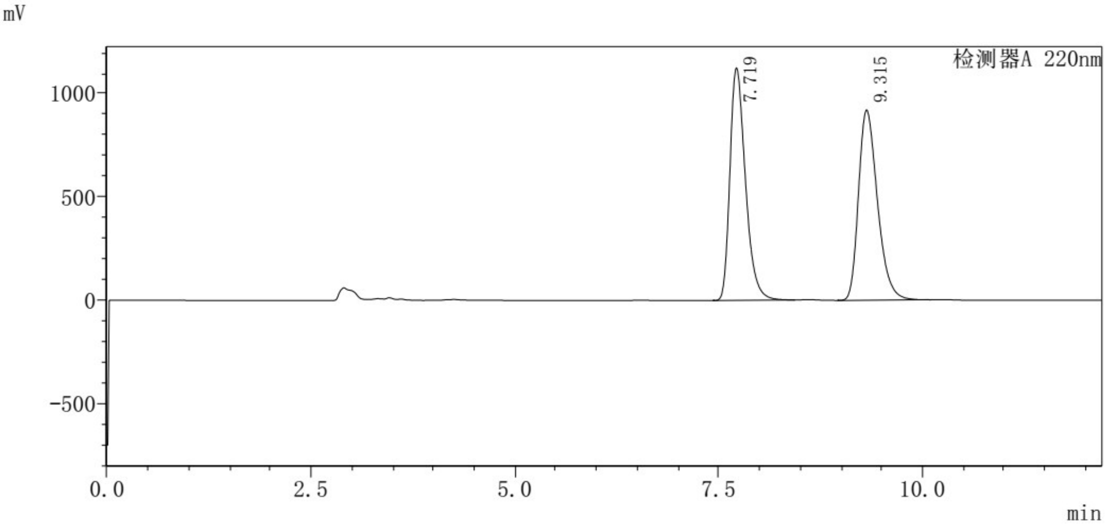

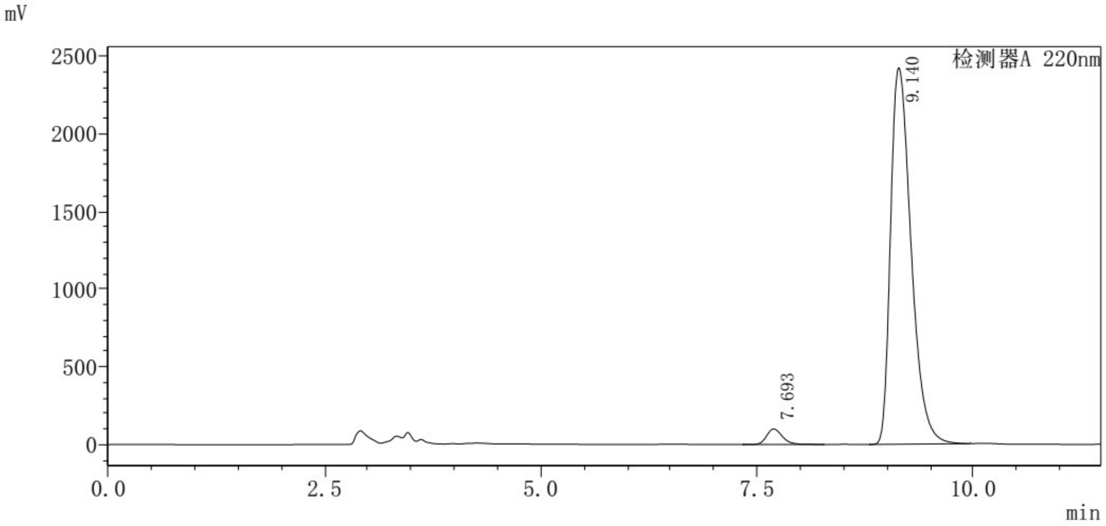

Peak#	Retention Time	Height	Area	Area%
1	7.693	99831	1257010	3.045
2	9.140	2422953	40018788	96.955
Total		2522784	41275798	100.000

(10) **2jc**

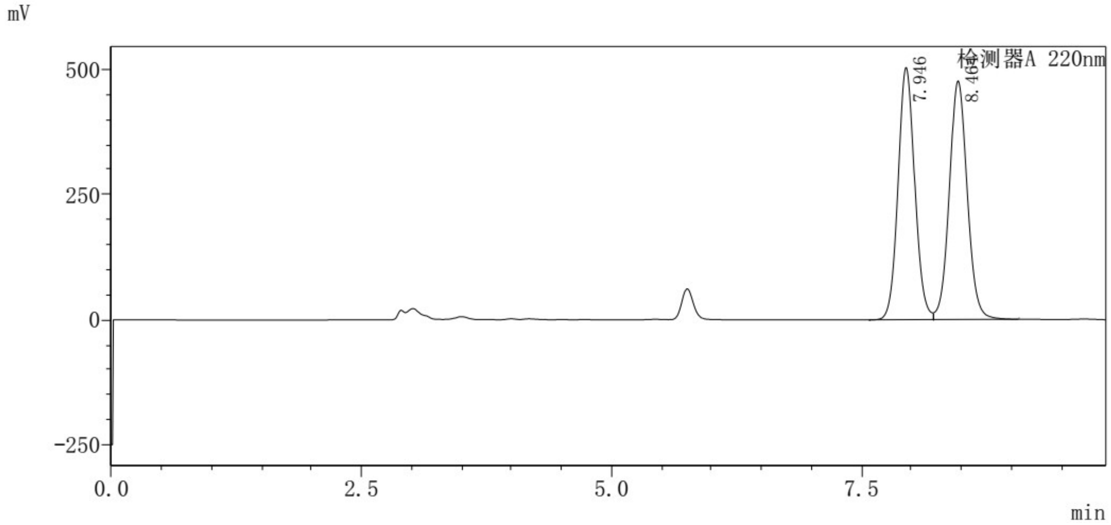

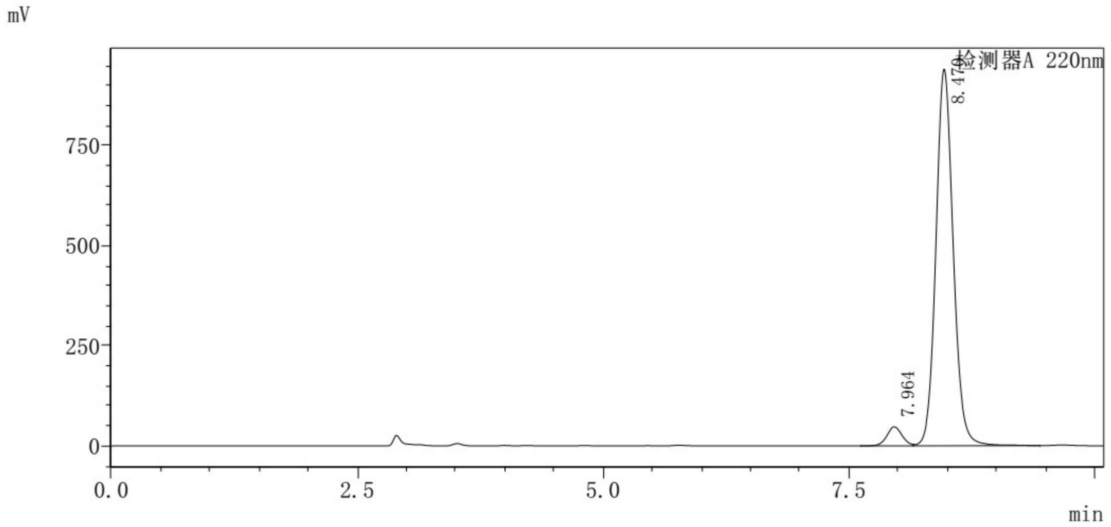

Peak#	Retention Time	Height	Area	Area%
1	7.964	47446	517391	4.324
2	8.470	940267	11447794	95.676
Total		987713	11965186	100.000

(11) **2kc**

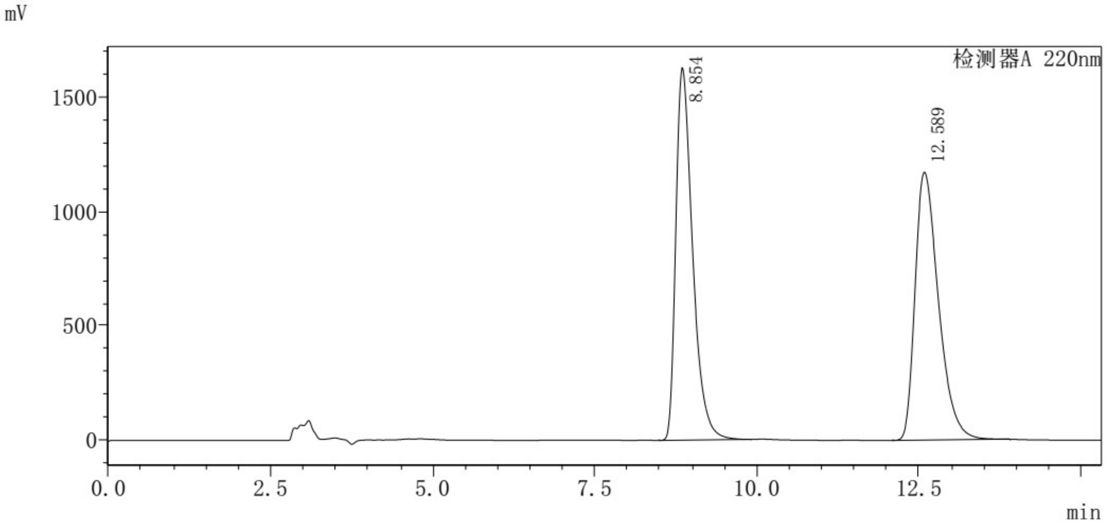

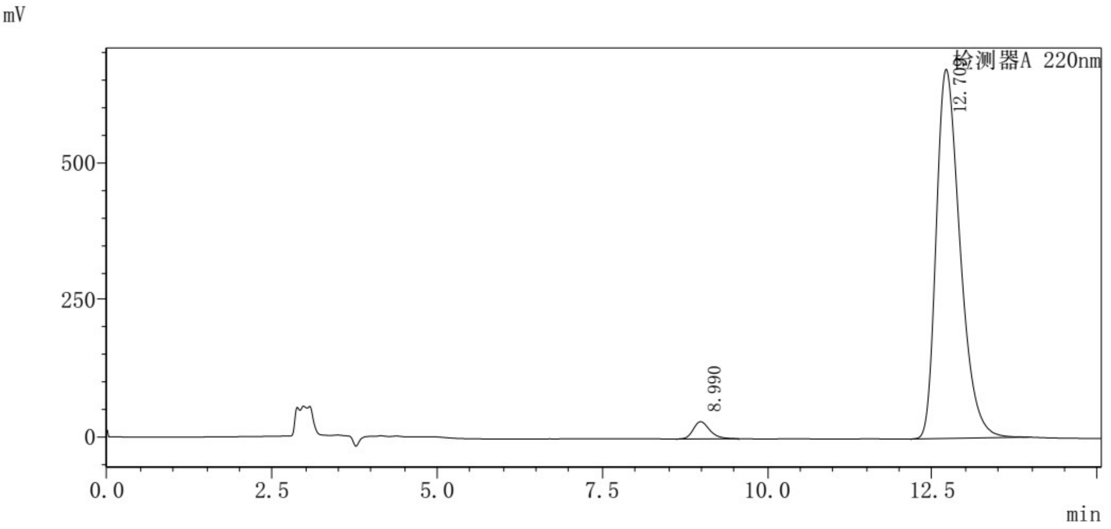

Peak#	Retention Time	Height	Area	Area%
1	8.990	31396	543844	3.120
2	12.709	674544	16889069	96.880
Total		705941	17432913	100.000

(12) **2lc**

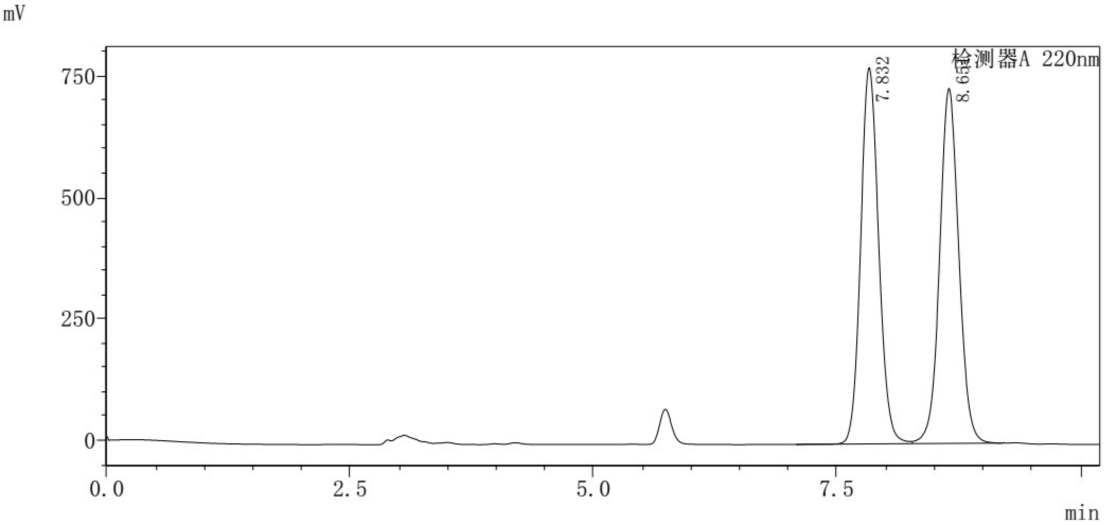

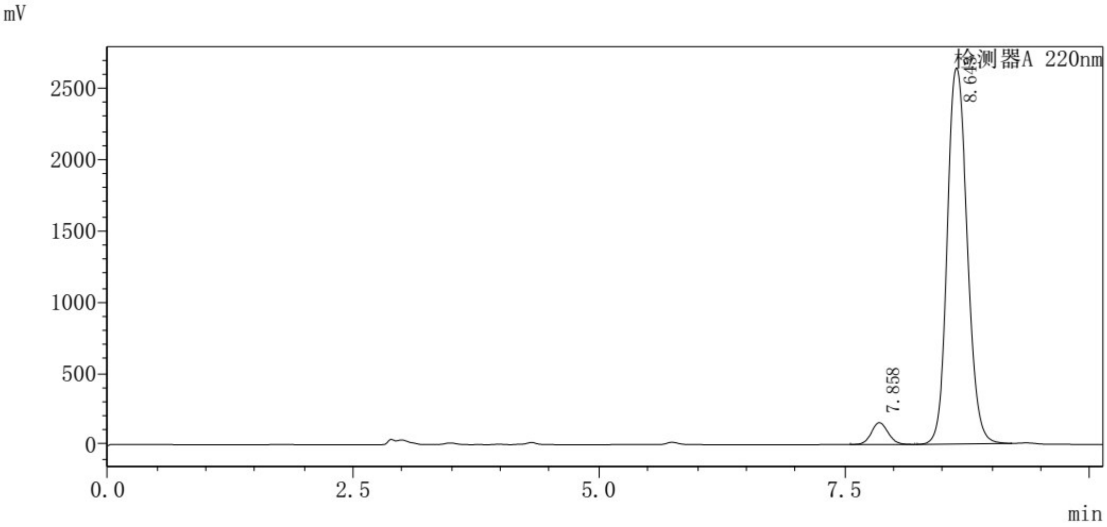

Peak#	Retention Time	Height	Area	Area%
1	7.858	153050	1814664	4.639
2	8.643	2640312	37300931	95.361
Total		2793362	39115595	100.000

(13) **2mc**

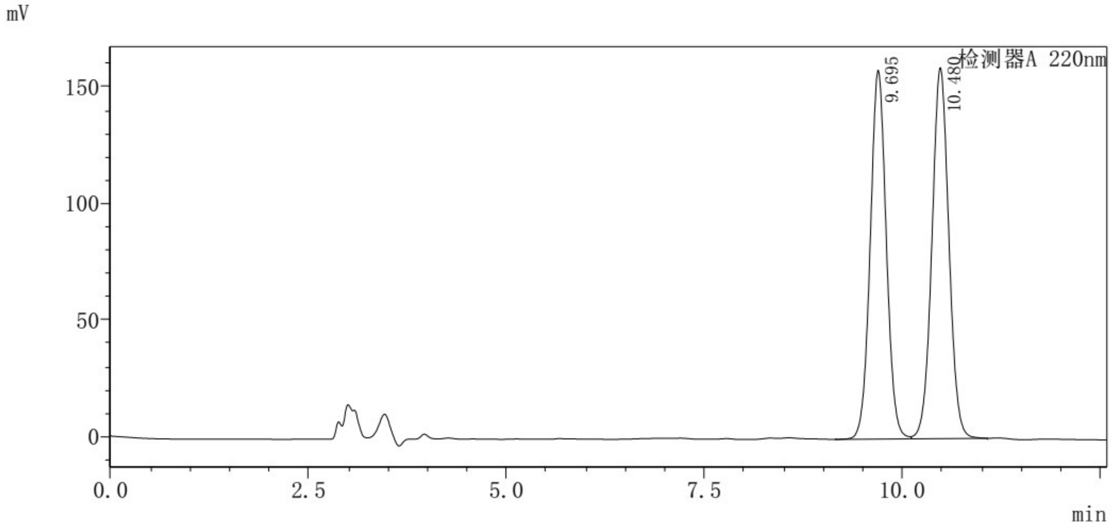

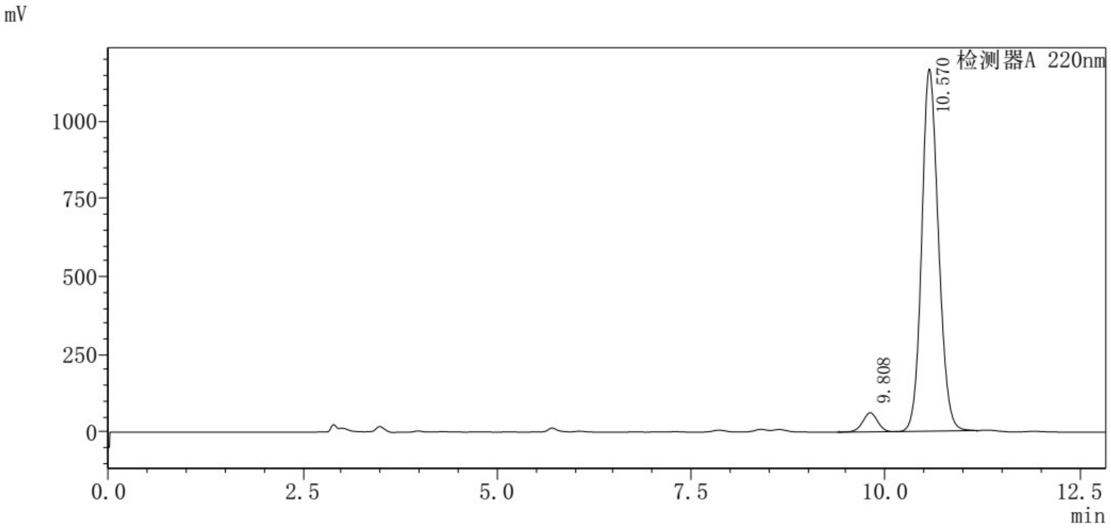

Peak#	Retention Time	Height	Area	Area%
1	9.808	61394	822347	4.504
2	10.570	1165648	17435755	95.496
Total		1227042	18258102	100.000

(14) **2nb**

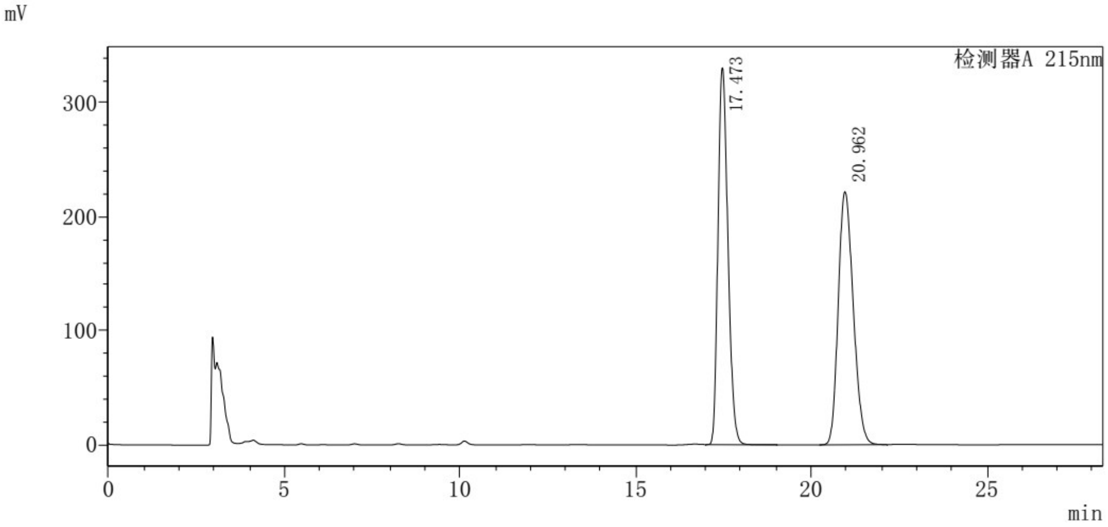

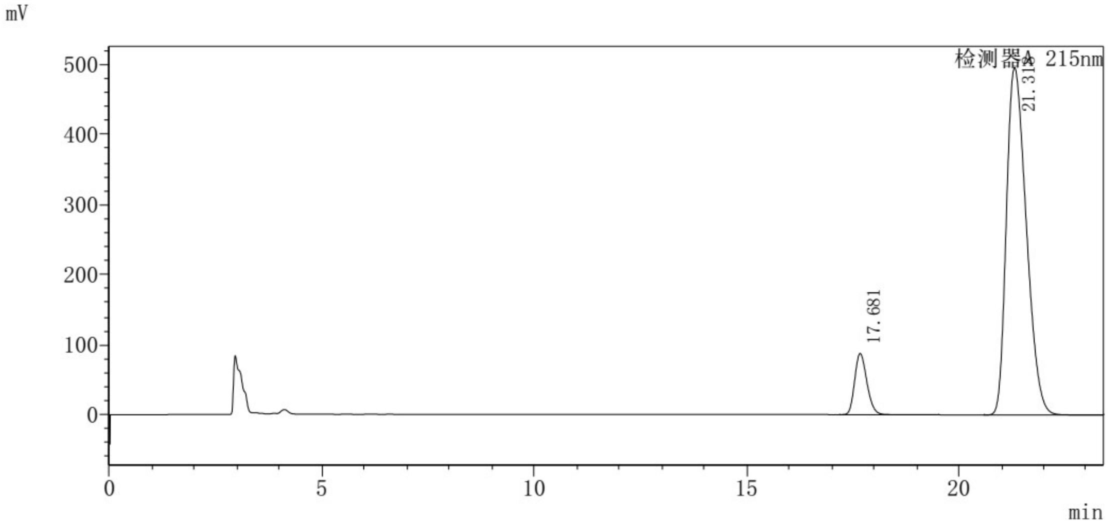

Peak#	Retention Time	Height	Area	Area%
1	17.681	87416	1749503	9.767
2	21.318	496385	16162782	90.233
Total		583801	17912285	100.000

(15) **2ab**

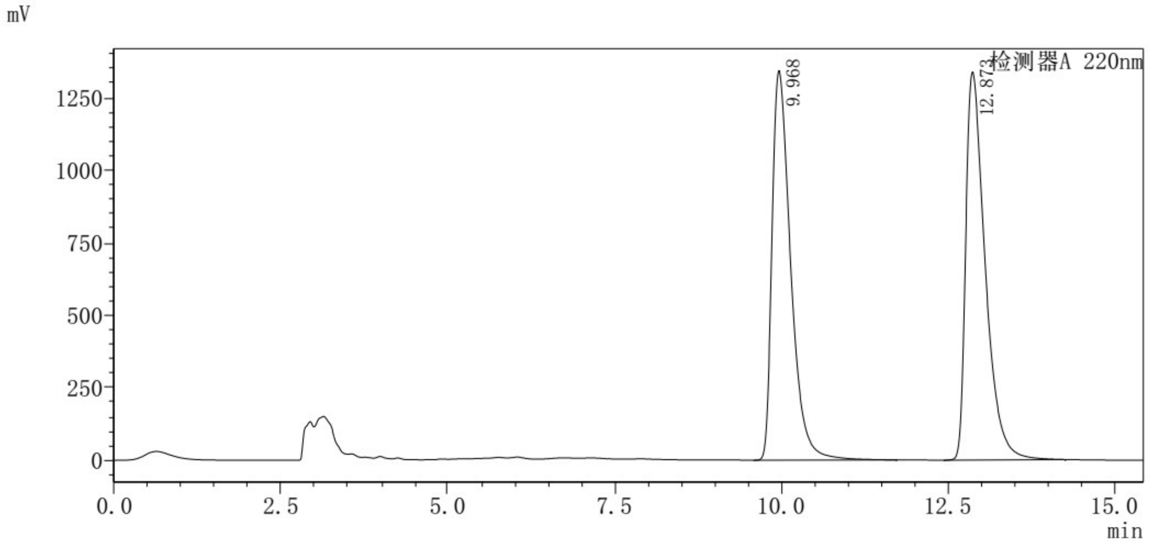

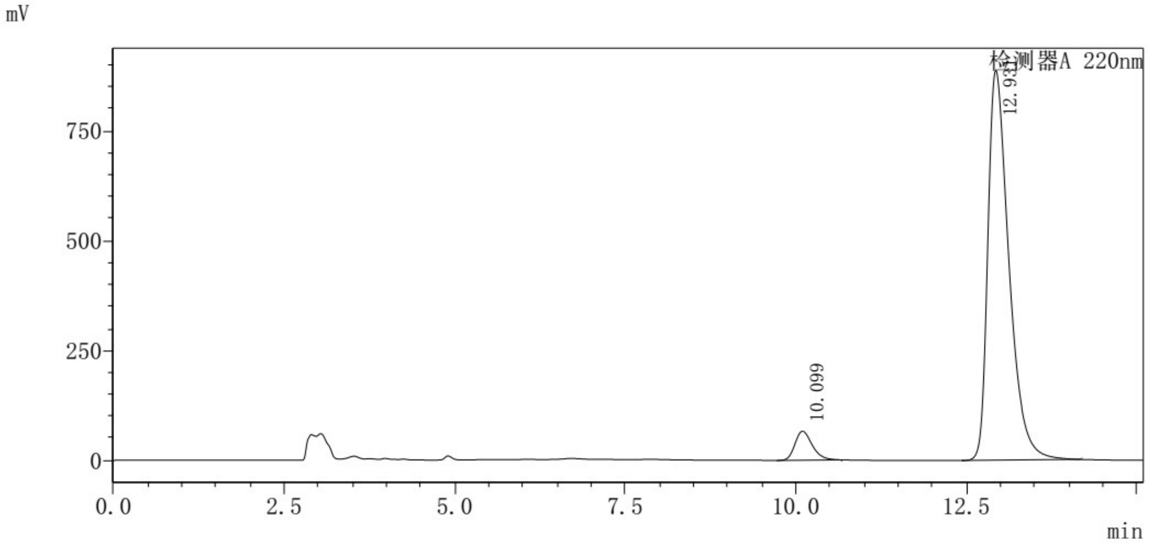

Peak#	Retention Time	Height	Area	Area%
1	10.099	65867	1153913	5.620
2	12.931	888286	19019594	94.380
Total		954152	20173507	100.000

(16) **4a**

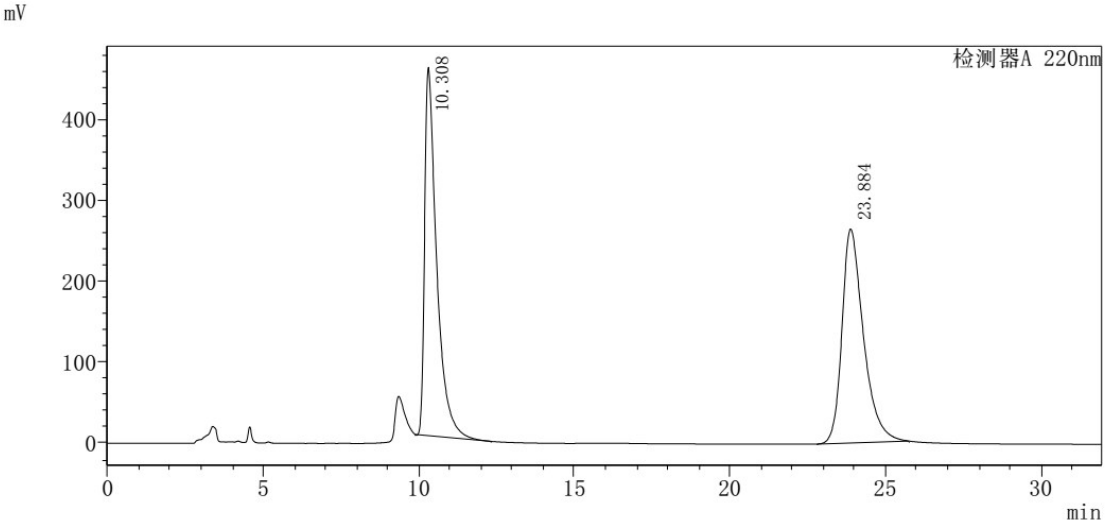

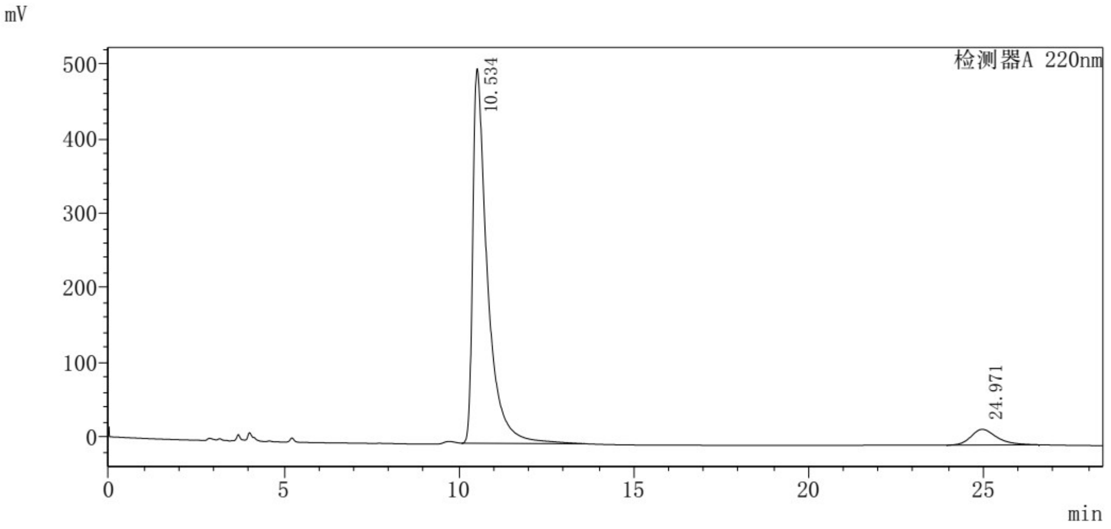

Peak#	Retention Time	Height	Area	Area%
1	10.534	502655	14596380	93.150
2	24.971	20953	1073376	6.850
Total		523608	15669756	100.000

(17) **4b**

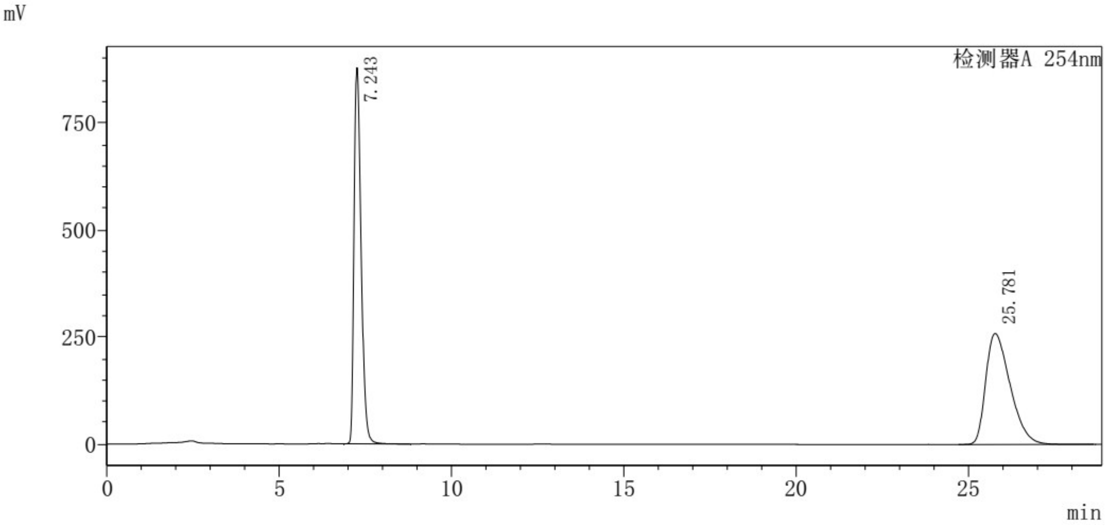

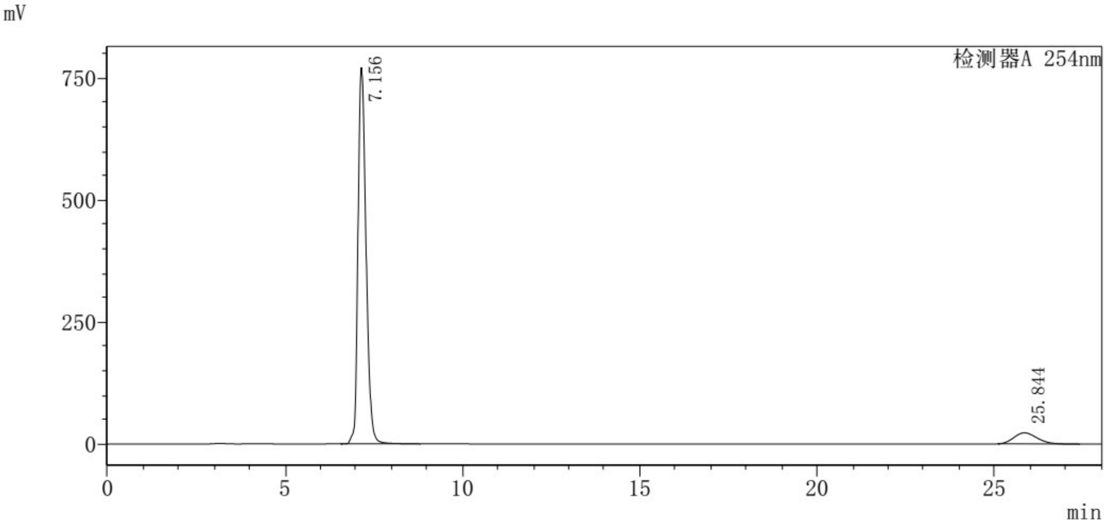

Peak#	Retention Time	Height	Area	Area%
1	7.156	770829	12182309	92.157
2	25.844	22767	1036726	7.843
Total		793596	13219034	100.000

(18) **4c**

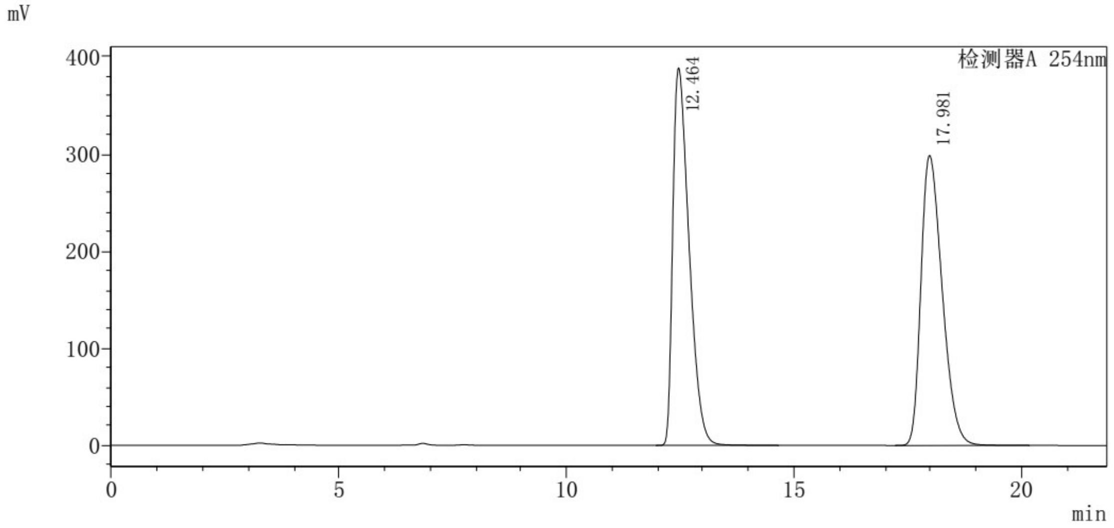

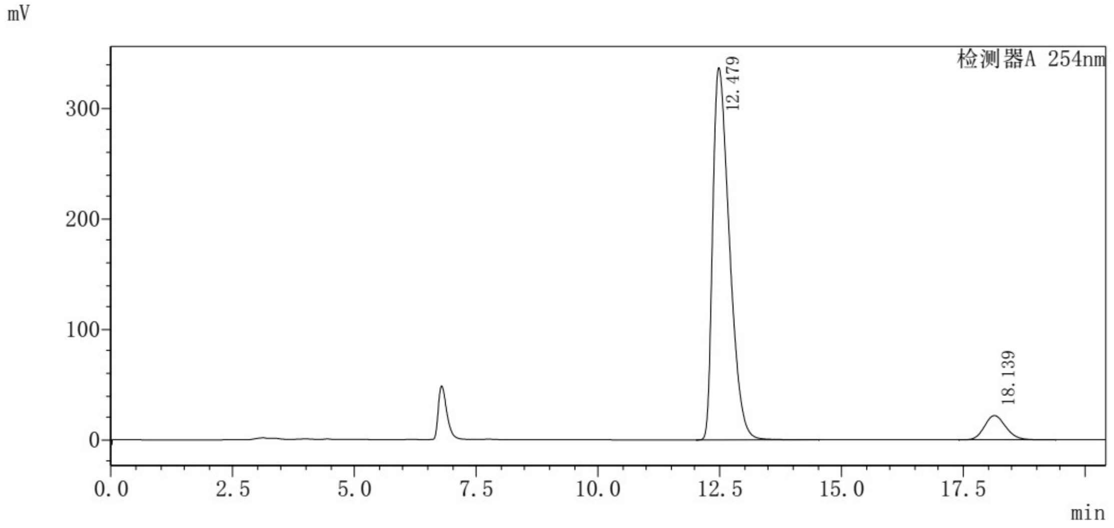

Peak#	Retention Time	Height	Area	Area%
1	12.479	336790	8045619	92.326
2	18.139	21963	668695	7.674
Total		358752	8714314	100.000

(19) **4d**

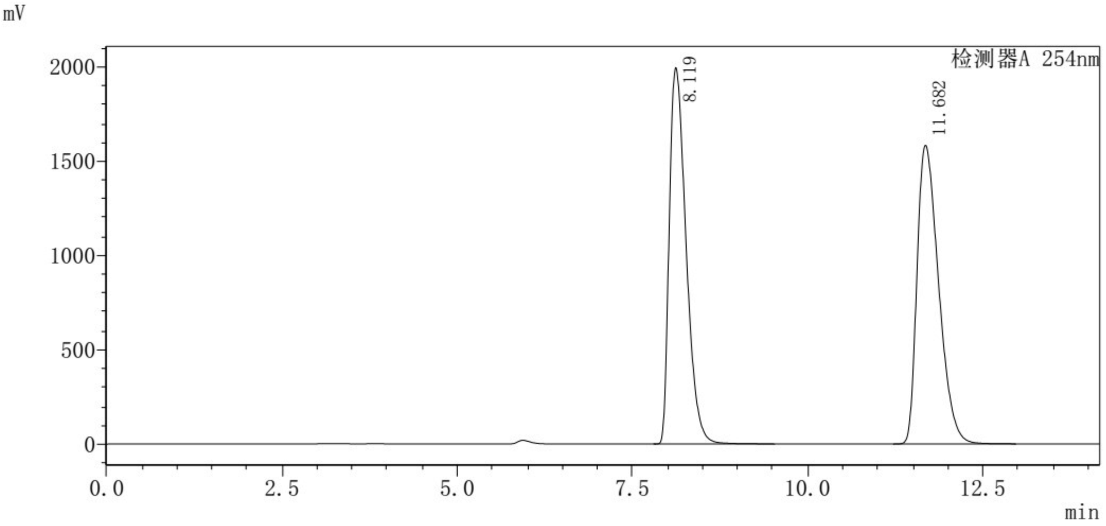

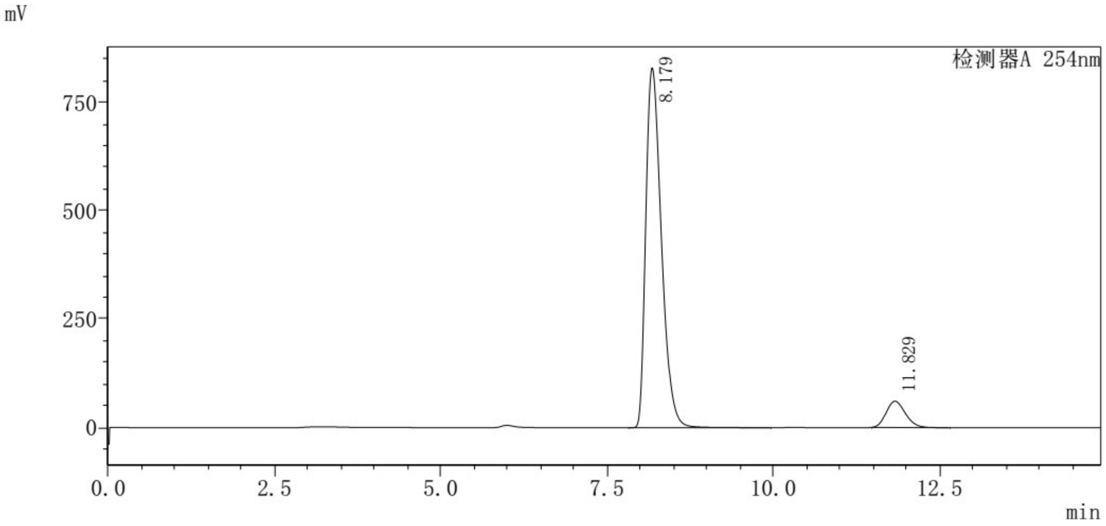

Peak#	Retention Time	Height	Area	Area%
1	8.179	829841	13570986	91.757
2	11.829	60468	1219173	8.243
Total		890308	14790158	100.000

(20) **4f**

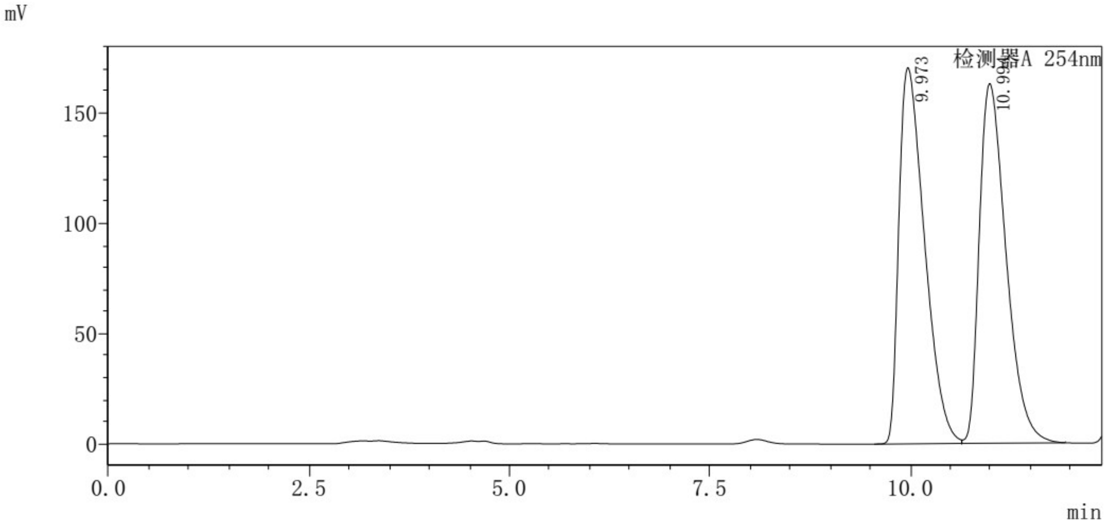

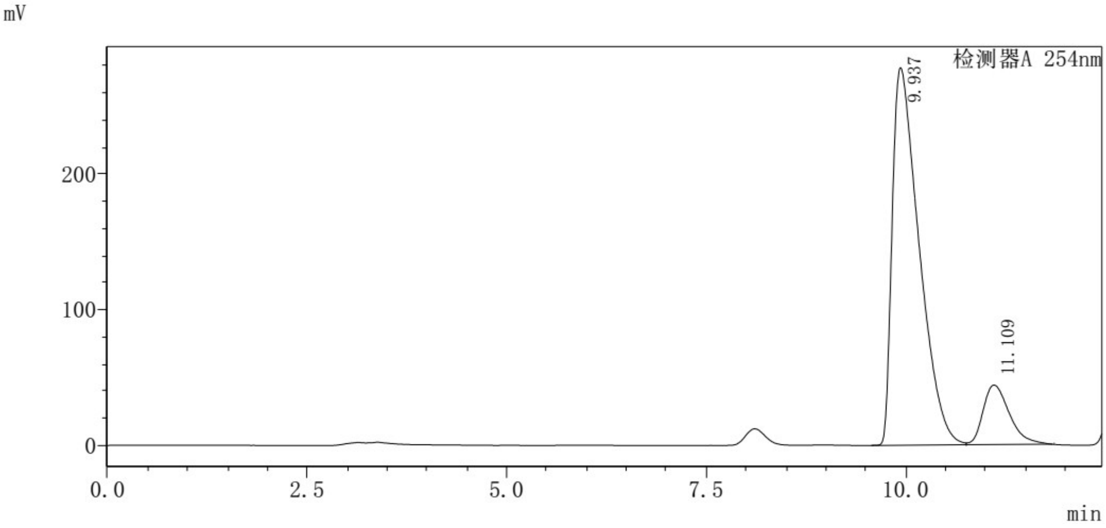

Peak#	Retention Time	Height	Area	Area%
1	9.937	278357	6516483	86.574
2	11.109	43972	1010559	13.426
Total		322329	7527042	100.000

(21) **6a**

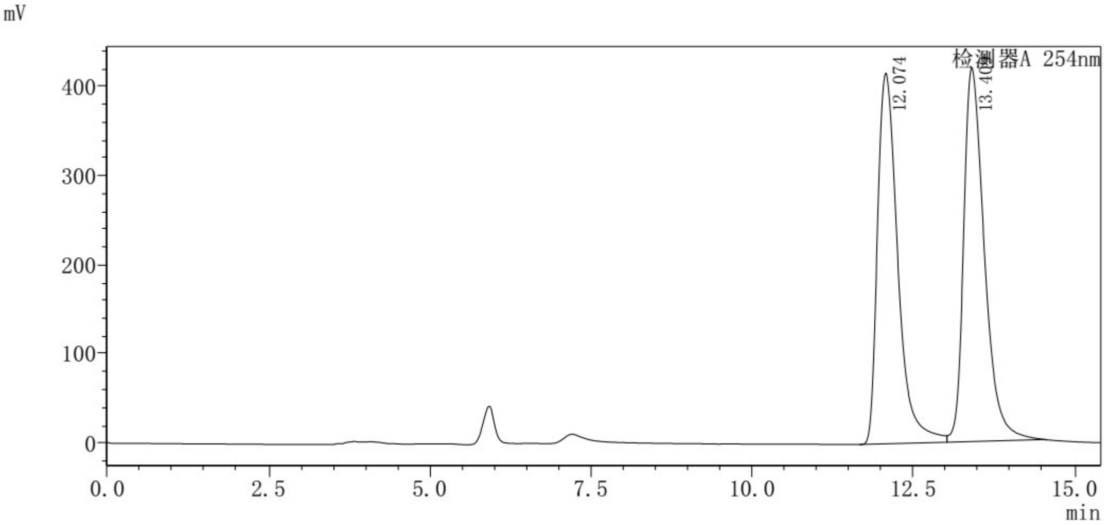

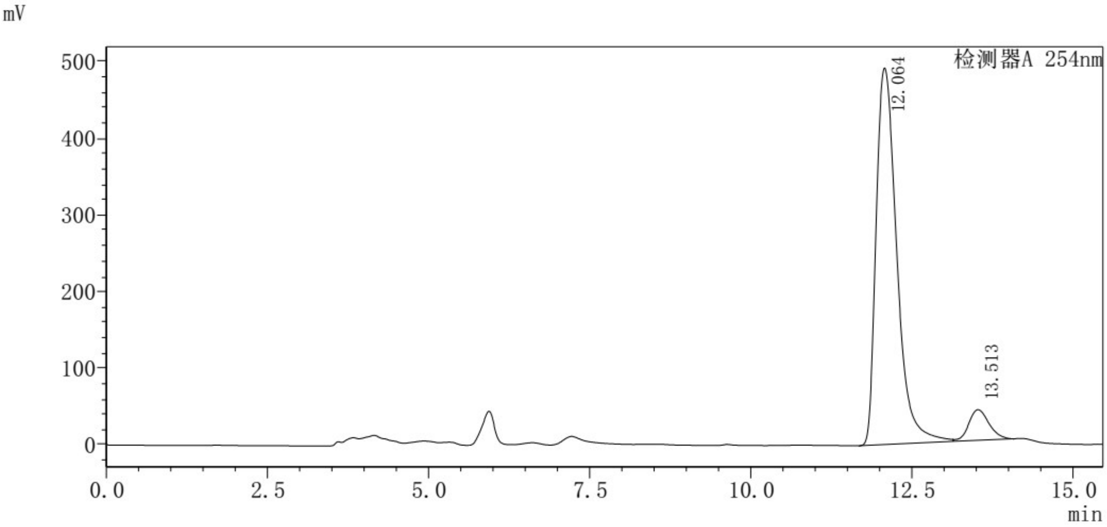

Peak#	Retention Time	Height	Area	Area%
1	12.064	491127	10939525	92.589
2	13.513	40067	875634	7.411
Total		531194	11815159	100.000

(22) **6b**

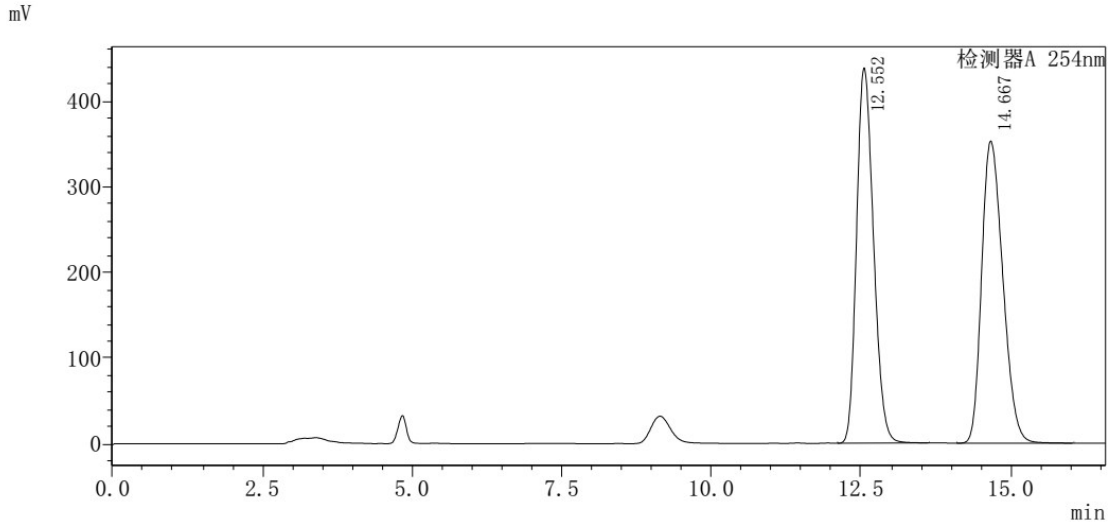

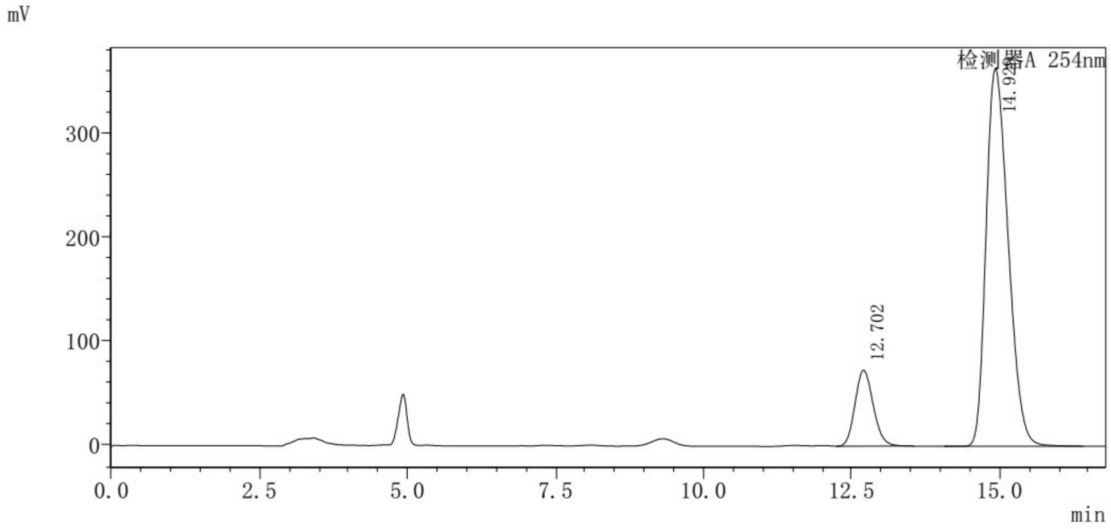

Peak#	Retention Time	Height	Area	Area%
1	12.702	73097	1566000	14.225
2	14.929	363361	9442757	85.775
Total		436458	11008757	100.000

(23) **6c**

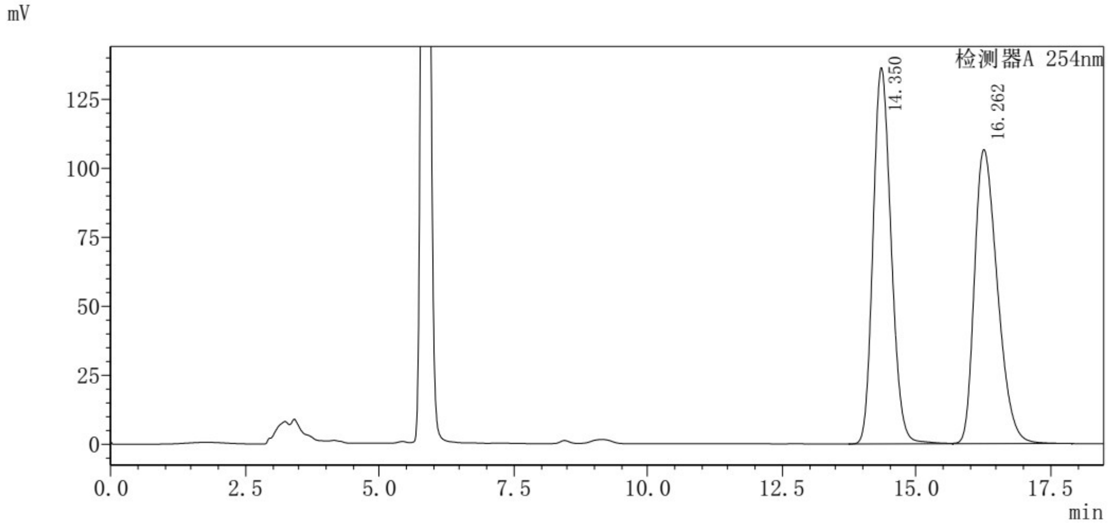

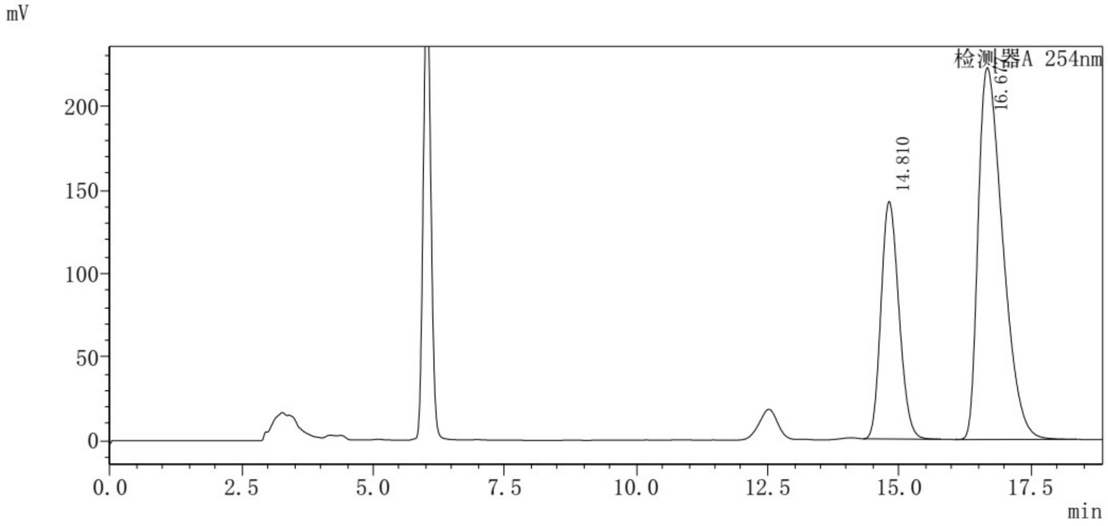

Peak#	Retention Time	Height	Area	Area%
1	14.810	142235	365023	31.782
2	16.677	222787	7250799	68.218
Total		365023	10628799	100.000

(24) **6d**

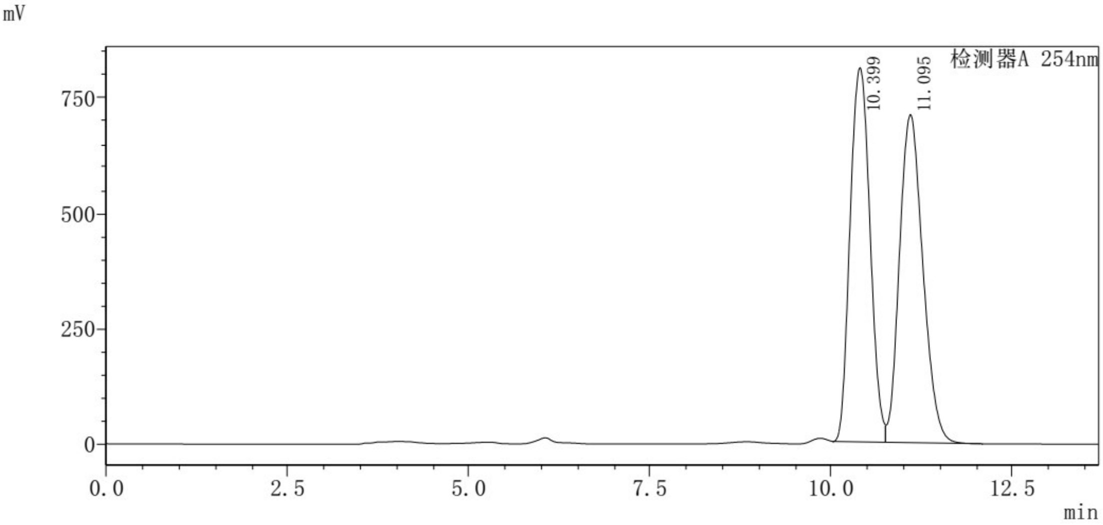

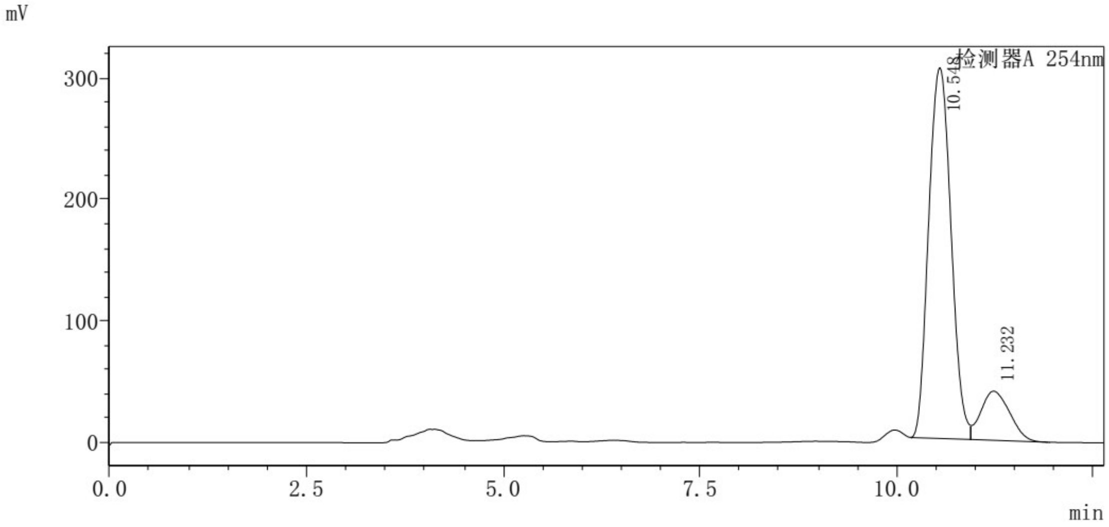

Peak#	Retention Time	Height	Area	Area%
1	10.548	305002	6028897	85.487
2	11.232	40480	1023541	14.513
Total		345482	7052438	100.000

(25) **6f**

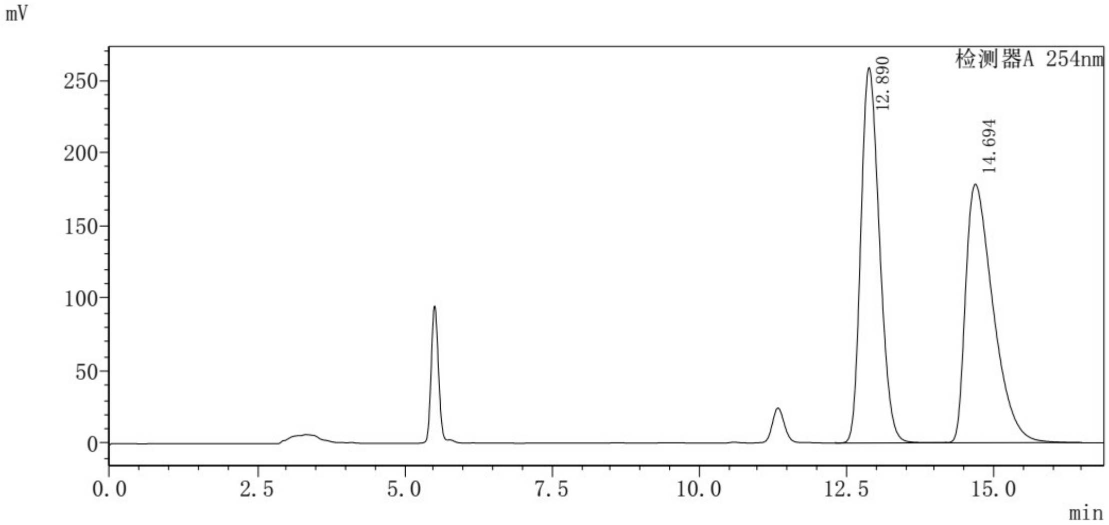

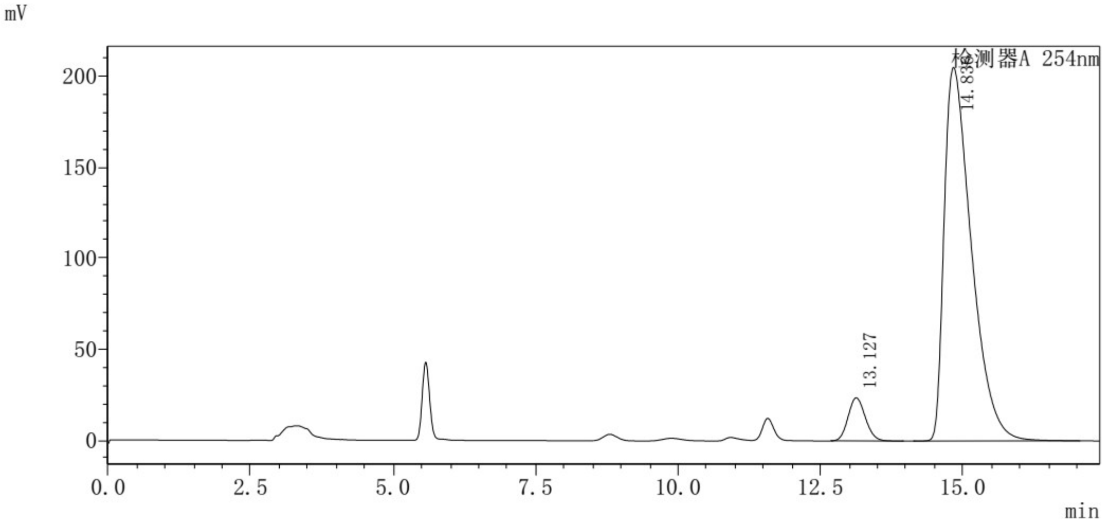

Peak#	Retention Time	Height	Area	Area%
1	13.127	23743	502035	6.833
2	14.836	205407	6845382	93.167
Total		229150	7347417	100.000

3. Optimizations of the Henry reaction of 2-Acylpyridines and 2-Acylpyridine *N*-Oxides

Table S1. Optimization of the Henry reaction of 2-Acylpyridine *N*-Oxides [a]

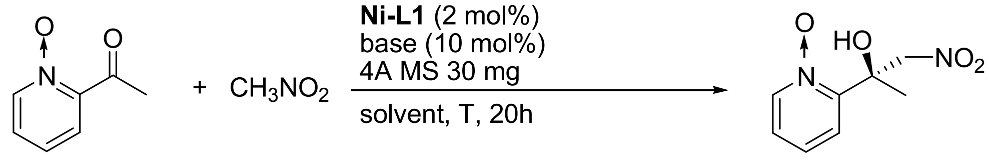

3a **4a**

Entry	Solvent	Base	T (°C)	Yield(%)[b]	Ee(%)[c]
1	THF	NMP[d]	35	94	46
2	THF	NMP[d]	0	51	50
3	MeOH	NMP[d]	0	84	70
4	EtOH	NMP[d]	0	66	76
5	CH_2Cl_2	NMP[d]	0	28	19
6	CH_3CN	NMP[d]	0	41	40
7	EtOH	EtN*i*Pr$_2$	0	99	62
8	EtOH	*i*Pr$_2$NH	0	99	63
9	EtOH	Et_3N	0	99	52
10	EtOH	*i*Pr$_2$NH	-20	99	78
11	EtOH	*i*Pr$_2$NH	-30	99	83
12	EtOH	*i*Pr$_2$NH	-40	97	85
13[e]	EtOH	*i*Pr$_2$NH	-40	92	86

[a] Reactions were carried out on a 0.2 mmol scale of 2-Acylpyridine *N*-Oxides **3a** in the mixture of solvent (0.8 mL) and nitromethane (0.2 mL) for 20h. The catalyst was pre-prepared. [b] Isolated yield. [c] Determined by HPLC analysis on a chiral stationary phase. [d] NMP = *N*-methylmorpholine. [e] *i*Pr$_2$NH (5 mol%).

Table S2 Optimization of the Henry reaction of 2-Acylpyridine *N*-Oxides [a]

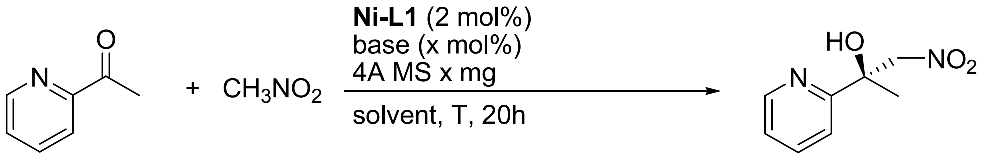

5a **6a**

Entry	Solvent	Base (mol %)	Additive	T (°C)	Yield(%)[b]	Ee(%)[c]
1	THF	NMP[d](10)	4Å MS (30 mg)	35	64	12
2	THF	NMP[d](10)	4Å MS (30 mg)	0	54	26
3	THF	EtN*i*Pr$_2$(10)	4Å MS (30 mg)	0	62	34

4	THF	iPr$_2$NH(10)	4Å MS (30 mg)	0	61	24
5	THF	Et$_3$N(10)	4Å MS (30 mg)	0	63	20
6	EtOH	EtNiPr$_2$(10)	4Å MS (30 mg)	0	69	16
7	CH_2Cl_2	EtNiPr$_2$(10)	4Å MS (30 mg)	0	46	23
8	EtOAc	EtNiPr$_2$(10)	4Å MS (30 mg)	0	34	19
9	CH_3CN	EtNiPr$_2$(10)	4Å MS (30 mg)	0	41	15
10	THF	EtNiPr$_2$(10)	4Å MS (30 mg)	-10	58	69
11	THF	EtNiPr$_2$(10)	4Å MS (30 mg)	-20	51	75
12	THF	EtNiPr$_2$(10)	4Å MS (30 mg)	-30	35	83
13	THF	EtNiPr$_2$(50)	4Å MS (30 mg)	-30	36	81
14	THF	EtNiPr$_2$(100)	4Å MS (30 mg)	-30	38	70
15	THF	EtNiPr$_2$(10)	4Å MS (20 mg)	-20	42	75
16	THF	EtNiPr$_2$(10)	4Å MS (40 mg)	-20	54	85
17	THF	EtNiPr$_2$(10)	4Å MS (50 mg)	-20	56	78

[a] Reactions were carried out on a 0.2 mmol scale of 2-Acylpyridine **5a** in the mixture of solvent (0.8 mL) and nitromethane (0.2 mL) for 20h. The catalyst was pre-prepared. [b] Isolated yield. [c] Determined by HPLC analysis on a chiral stationary phase. [d] NMP = *N*-methylmorpholine.

4. Selected NMR spectra

2ac

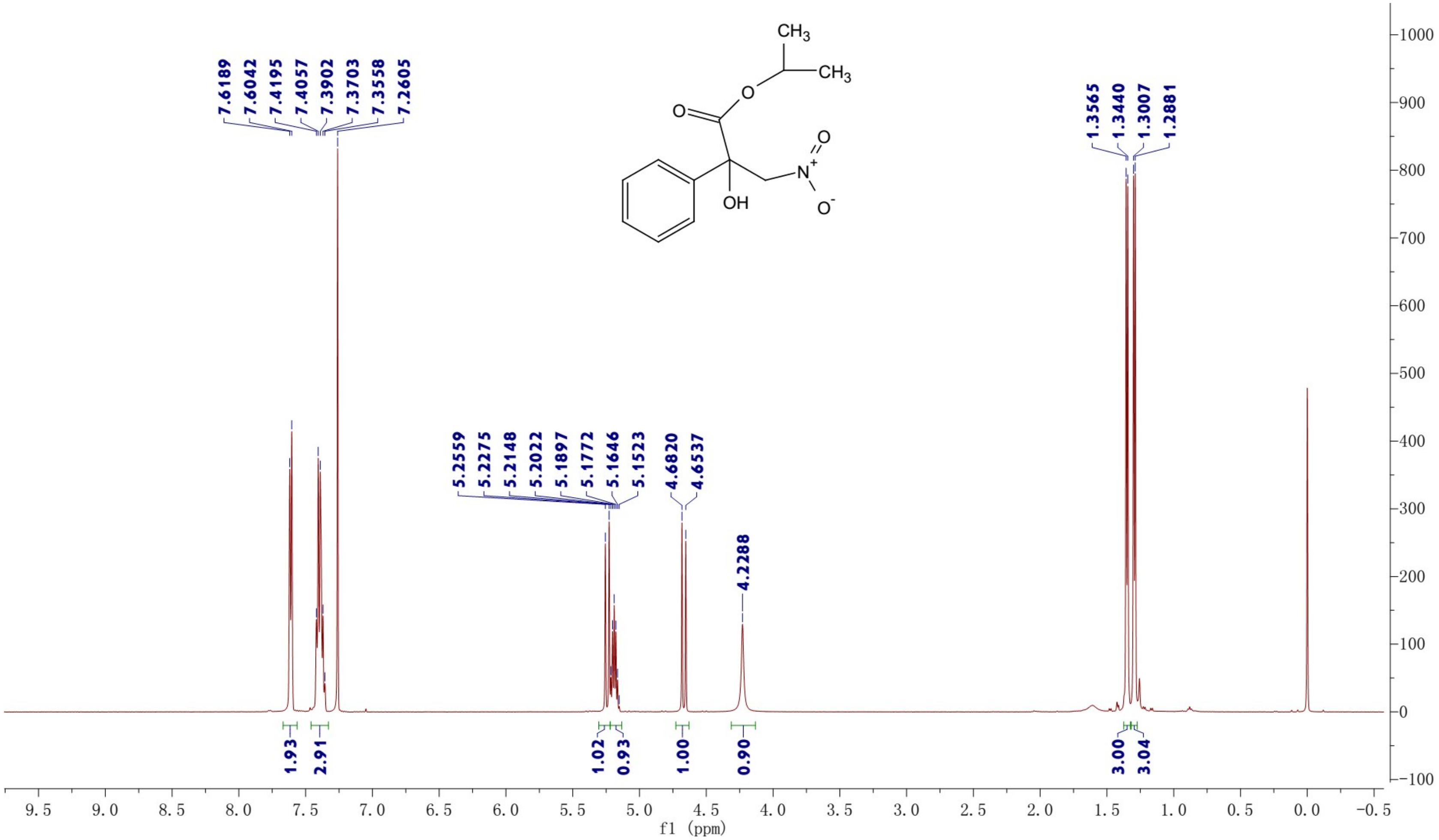

2ac

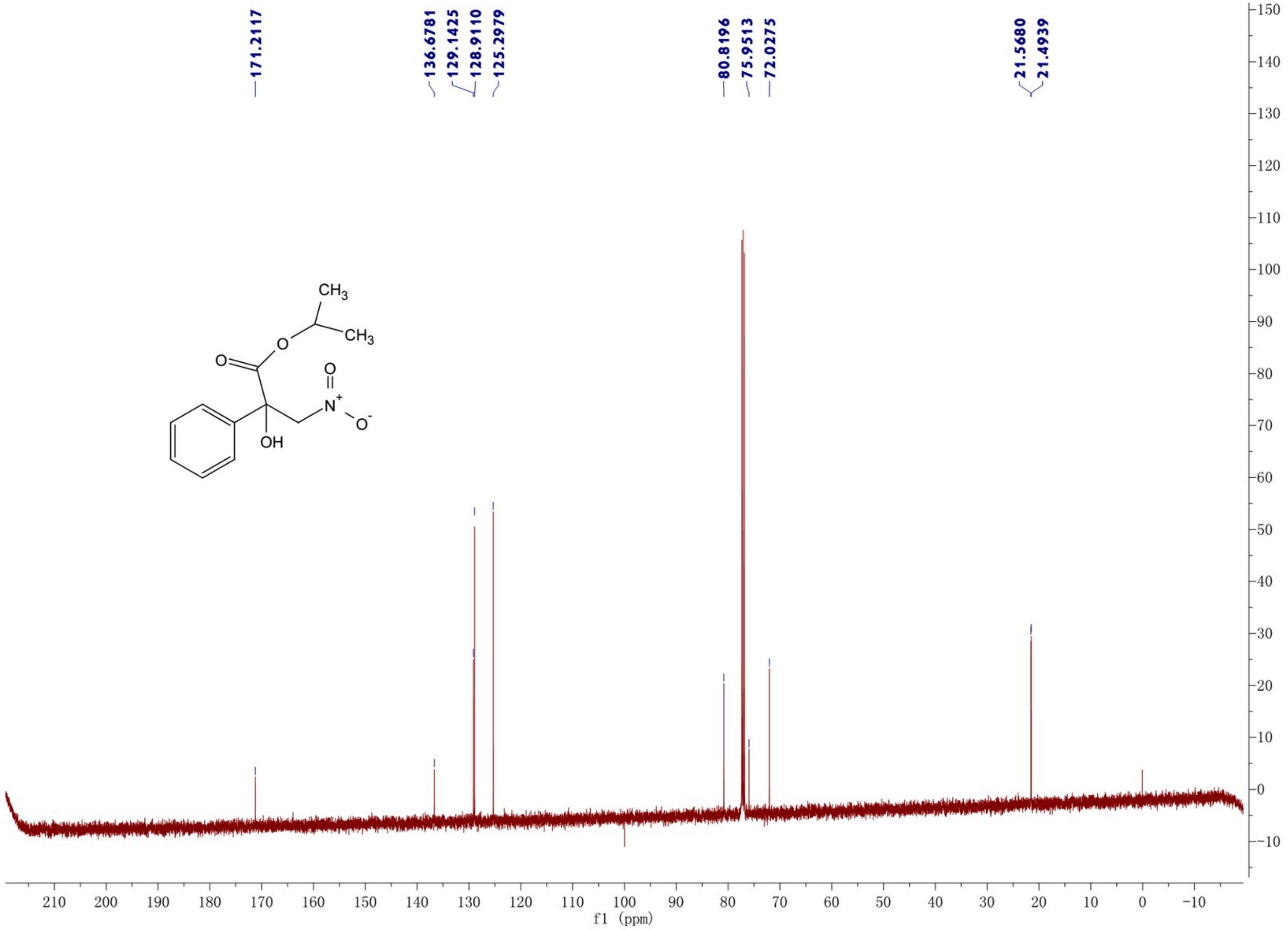
171.2117
136.6781
129.1425
128.9110
125.2979
80.8196
75.9513
72.0275
21.5680
21.4939
CH3
CH3
O
O
O
N+
O-
OH
150
140
130
120
110
100
90
80
70
60
50
40
30
20
10
0
-10
210
200
190
180
170
160
150
140
130
120
110
100
90
80
70
60
50
40
30
20
10
0
-10
f1 (ppm)

2bc

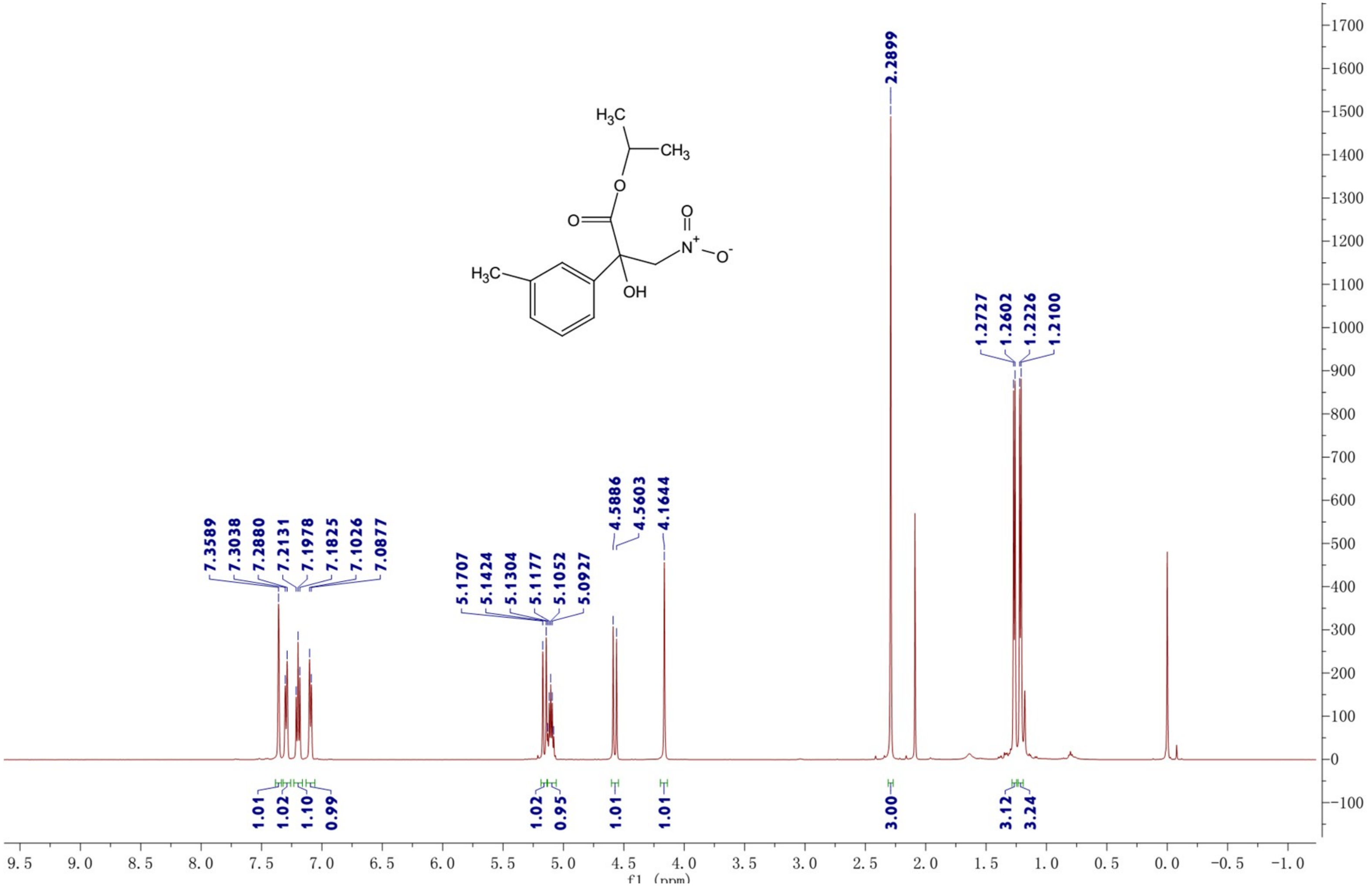

H3C
CH3
O
O
O
N+
O-
H3C
OH
2.2899
1.2727
1.2602
1.2226
1.2100
4.5886
4.5603
4.1644
7.3589
7.3038
7.2880
7.2131
7.1978
7.1825
7.1026
7.0877
5.1707
5.1424
5.1304
5.1177
5.1052
5.0927
1.01
1.02
1.10
0.99
1.02
0.95
1.01
1.01
3.00
3.12
3.24
f1 (ppm)

2bc

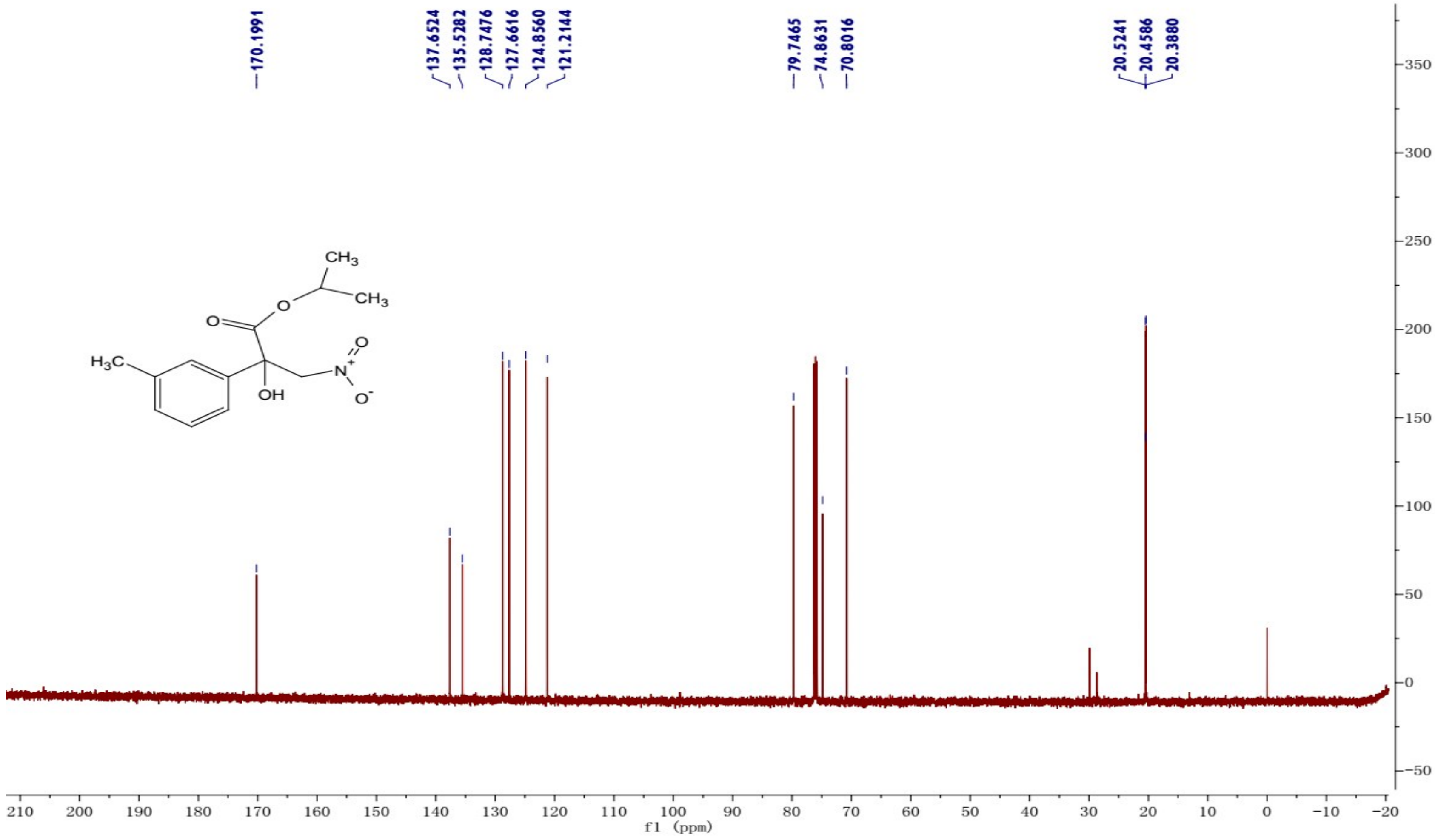

170.1991
137.6524
135.5282
128.7476
127.6616
124.8560
121.2144
79.7465
74.8631
70.8016
20.5241
20.4586
20.3880
CH3
CH3
O
O
H3C
OH
N+
O
O-
f1 (ppm)

2cc

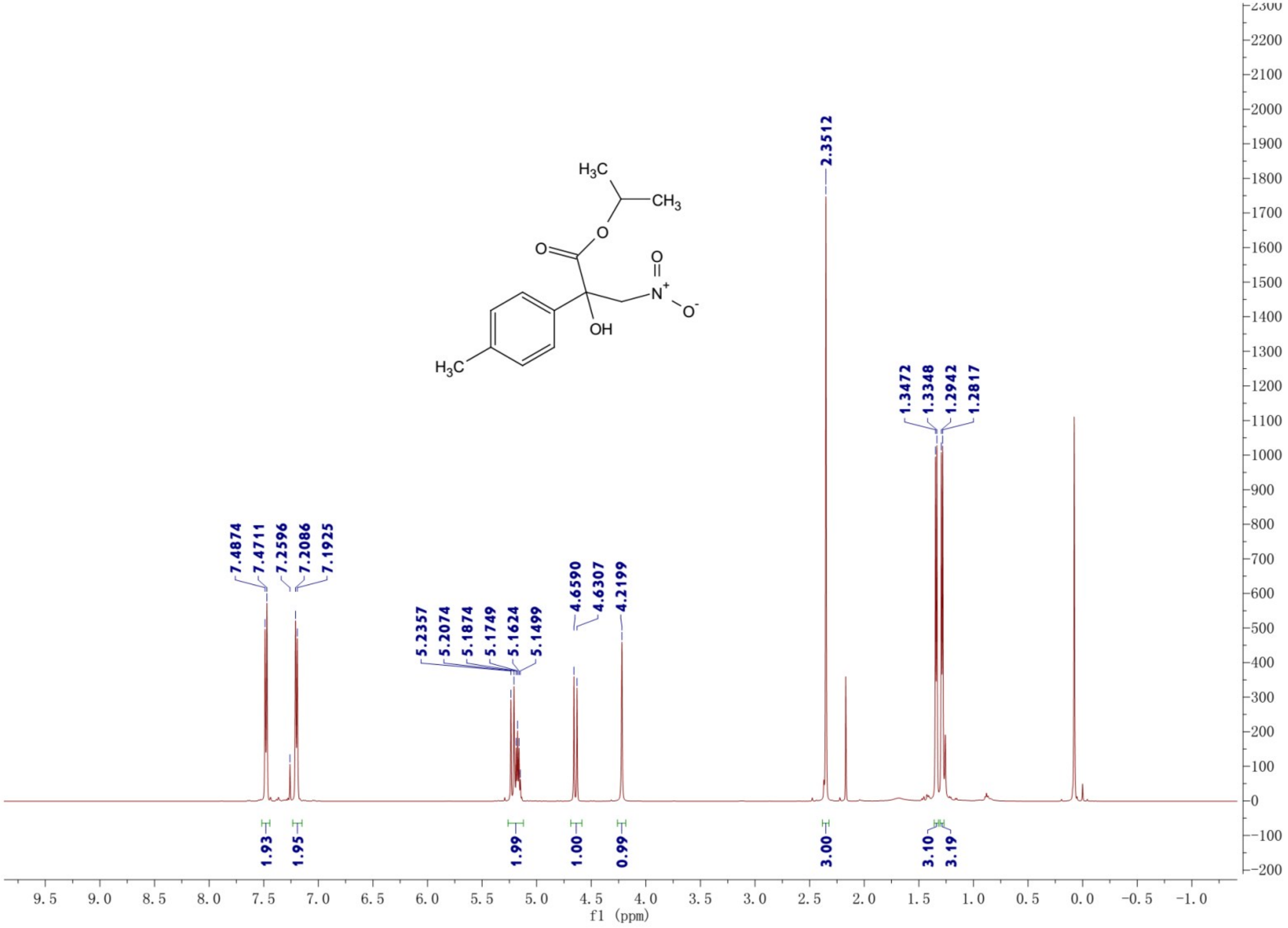

H3C
CH3
O
O
O
N+
O-
OH
H3C
7.4874
7.4711
7.2596
7.2086
7.1925
5.2357
5.2074
5.1874
5.1749
5.1624
5.1499
4.6590
4.6307
4.2199
2.3512
1.3472
1.3348
1.2942
1.2817
1.93
1.95
1.99
1.00
0.99
3.00
3.10
3.19
f1 (ppm)

2cc

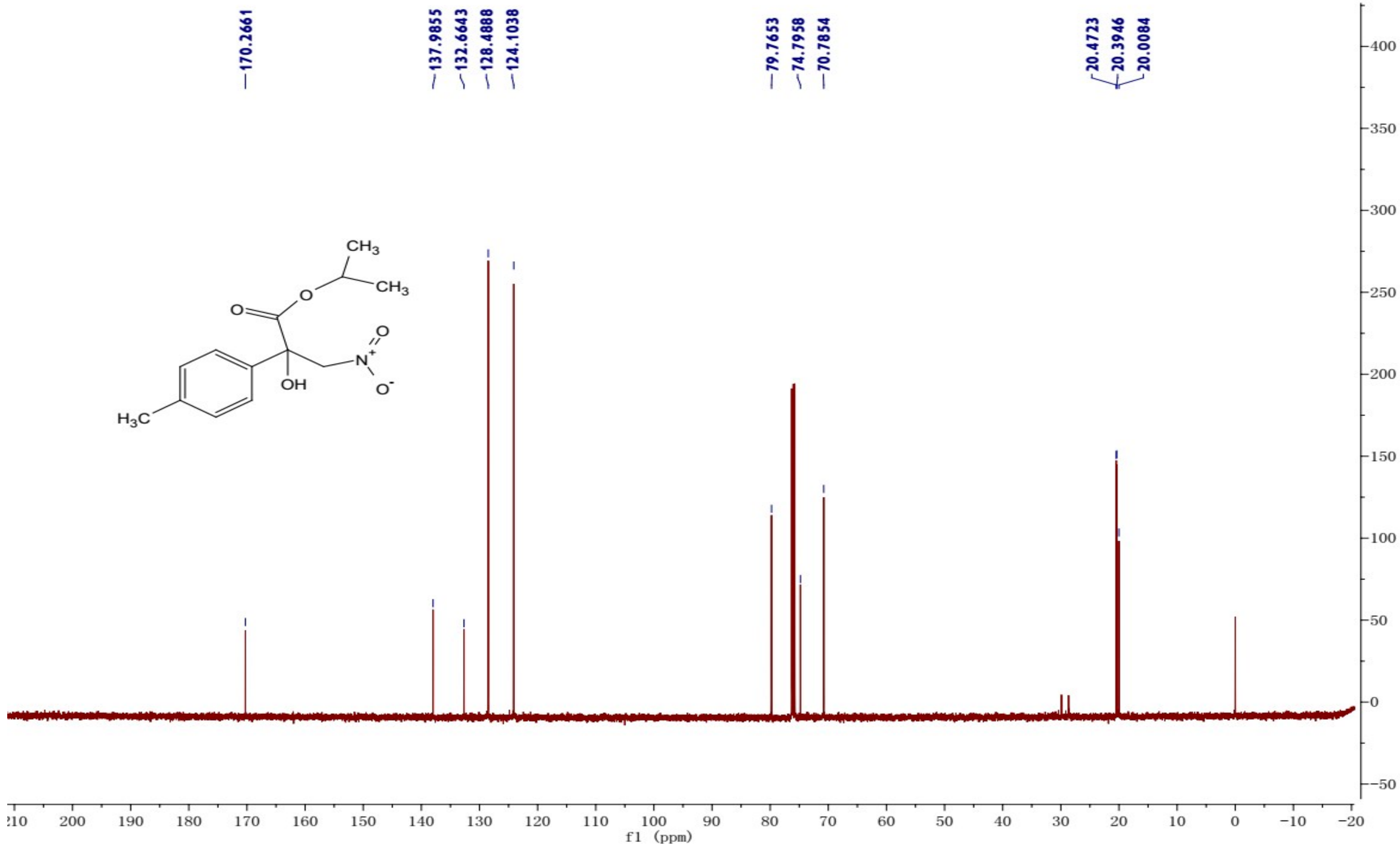

170.2661
137.9855
132.6643
128.4888
124.1038
79.7653
74.7958
70.7854
20.4723
20.3946
20.0084
CH3
CH3
O
O
OH
N+
O
O-
H3C
f1 (ppm)

2dc

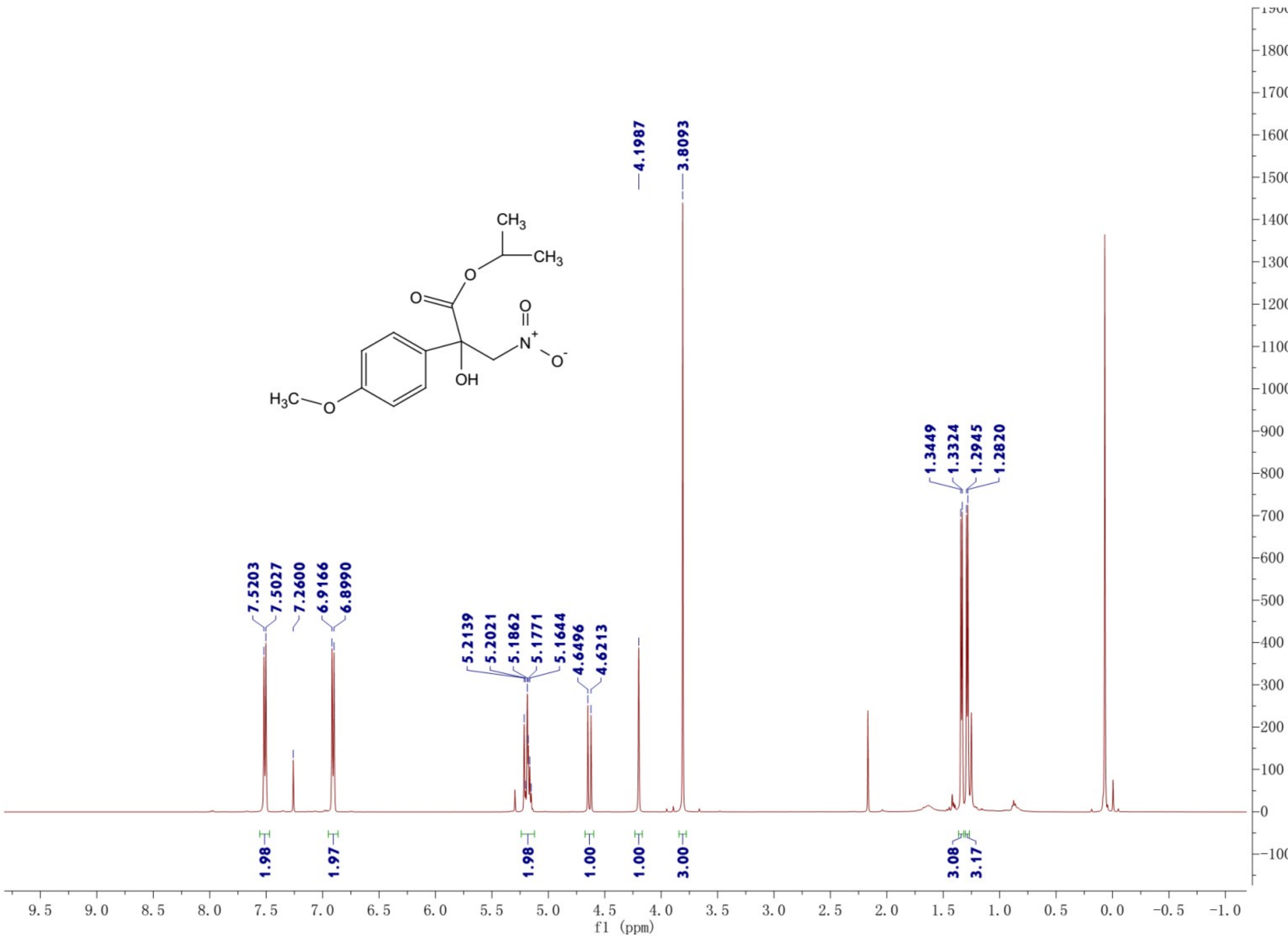

7.5203
7.5027
7.2600
6.9166
6.8990
5.2139
5.2021
5.1862
5.1771
5.1644
4.6496
4.6213
4.1987
3.8093
1.3449
1.3324
1.2945
1.2820
1.98
1.97
1.98
1.00
1.00
3.00
3.08
3.17
f1 (ppm)
CH3
CH3
O
O
N+
O
O-
OH
H3C
O

2dc

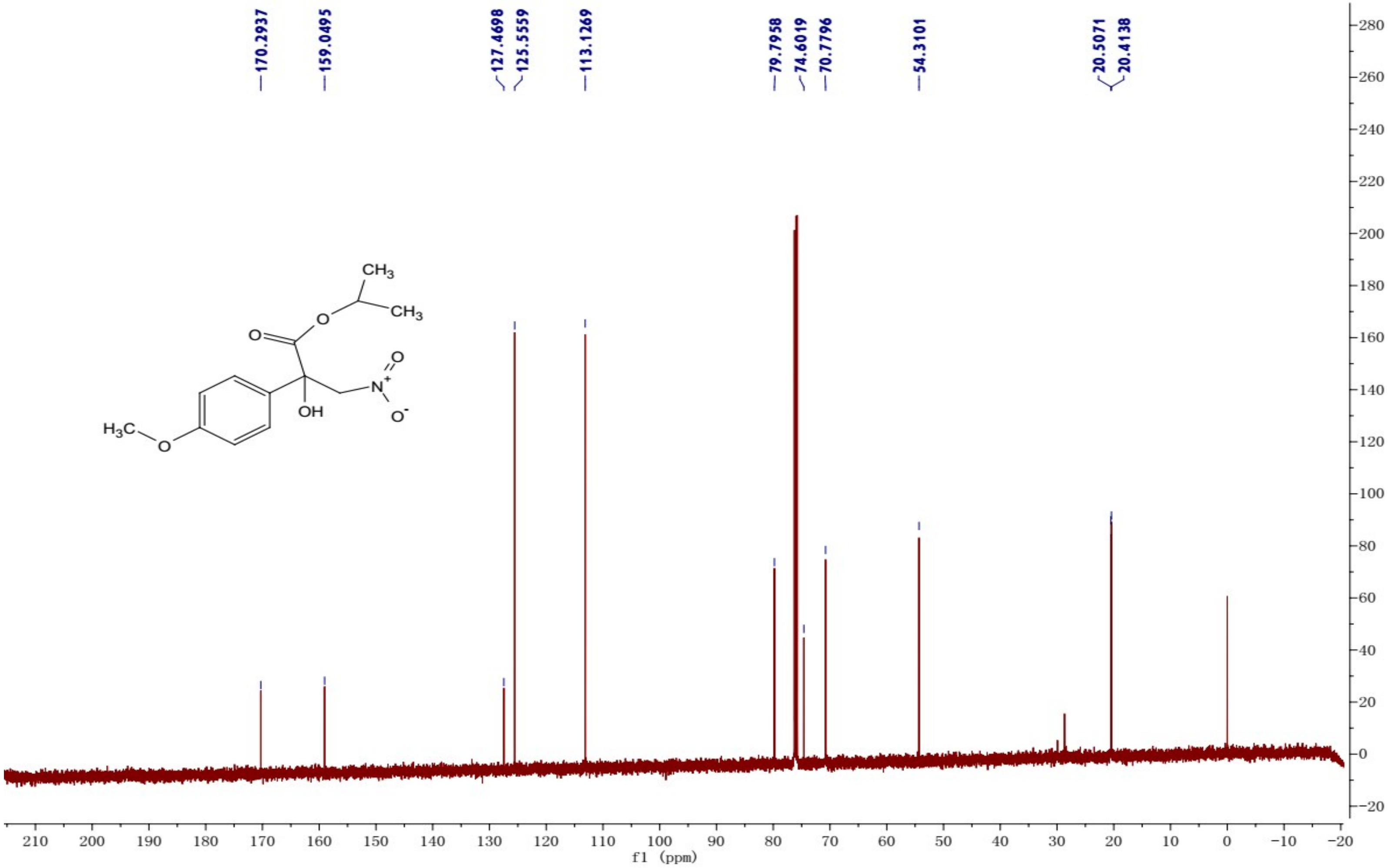
170.2937
159.0495
127.4698
125.5559
113.1269
79.7958
74.6019
70.7796
54.3101
20.5071
20.4138
f1 (ppm)

2ec

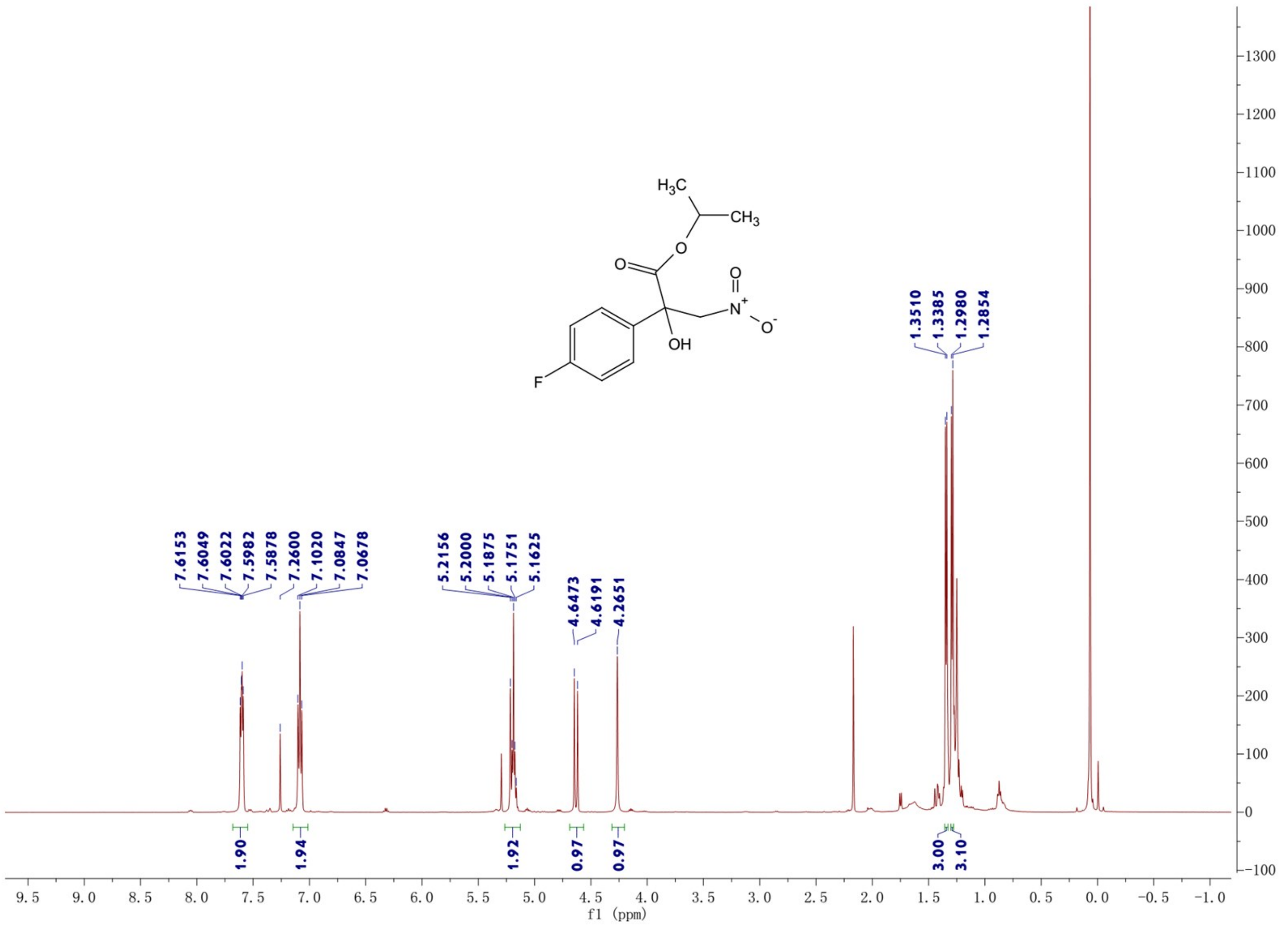
H3C
CH3
O
O
O
N+
O-
OH
F
7.6153
7.6049
7.6022
7.5982
7.5878
7.2600
7.1020
7.0847
7.0678
5.2156
5.2000
5.1875
5.1751
5.1625
4.6473
4.6191
4.2651
1.3510
1.3385
1.2980
1.2854
1.90
1.94
1.92
0.97
0.97
3.00
3.10
f1 (ppm)

2ec

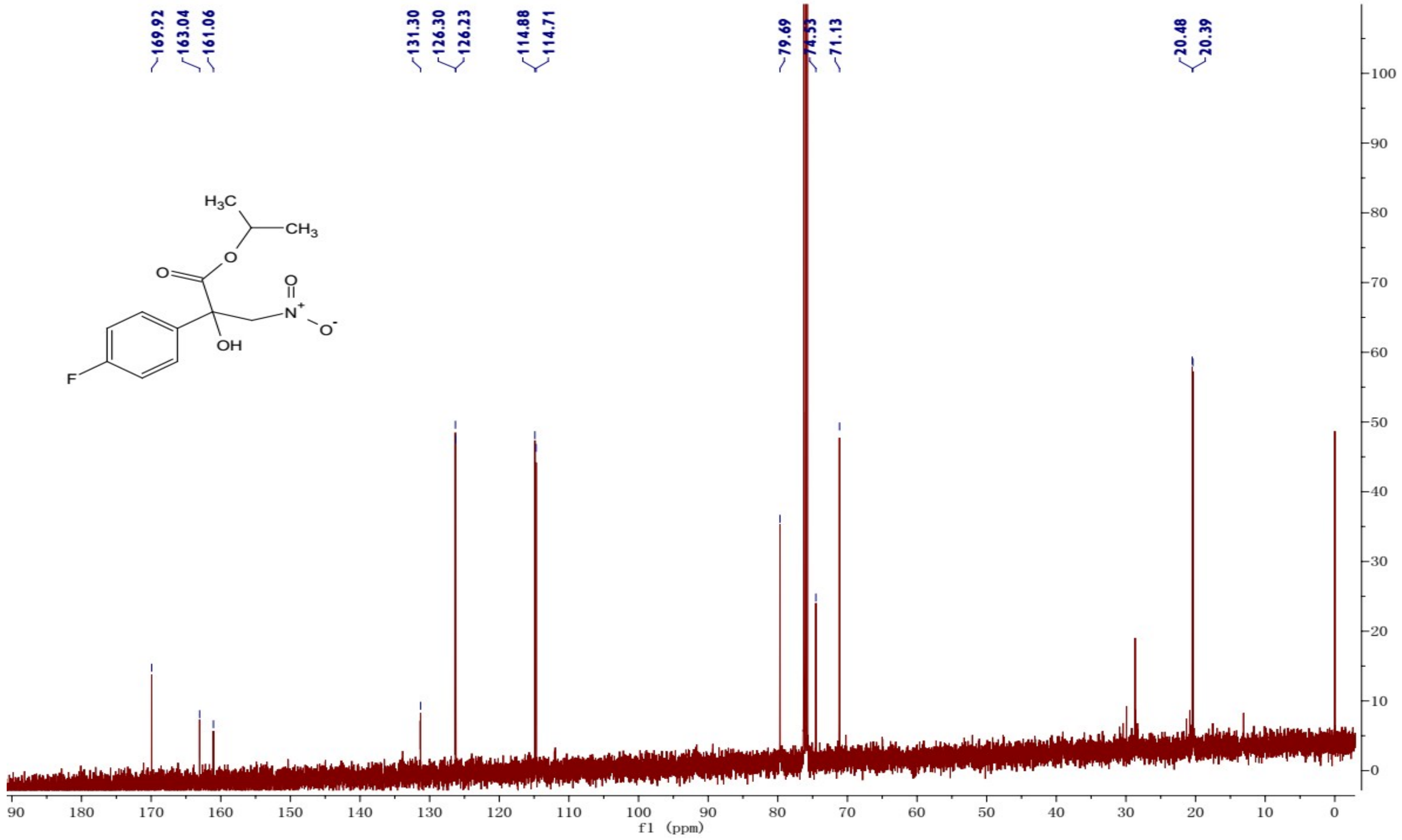
169.92
163.04
161.06
131.30
126.30
126.23
114.88
114.71
79.69
74.53
71.13
20.48
20.39
H_3C
CH_3
O
O
O
N^+
O^-
OH
F
100
90
80
70
60
50
40
30
20
10
0
90
180
170
160
150
140
130
120
110
100
90
80
70
60
50
40
30
20
10
0
f1 (ppm)

2fc

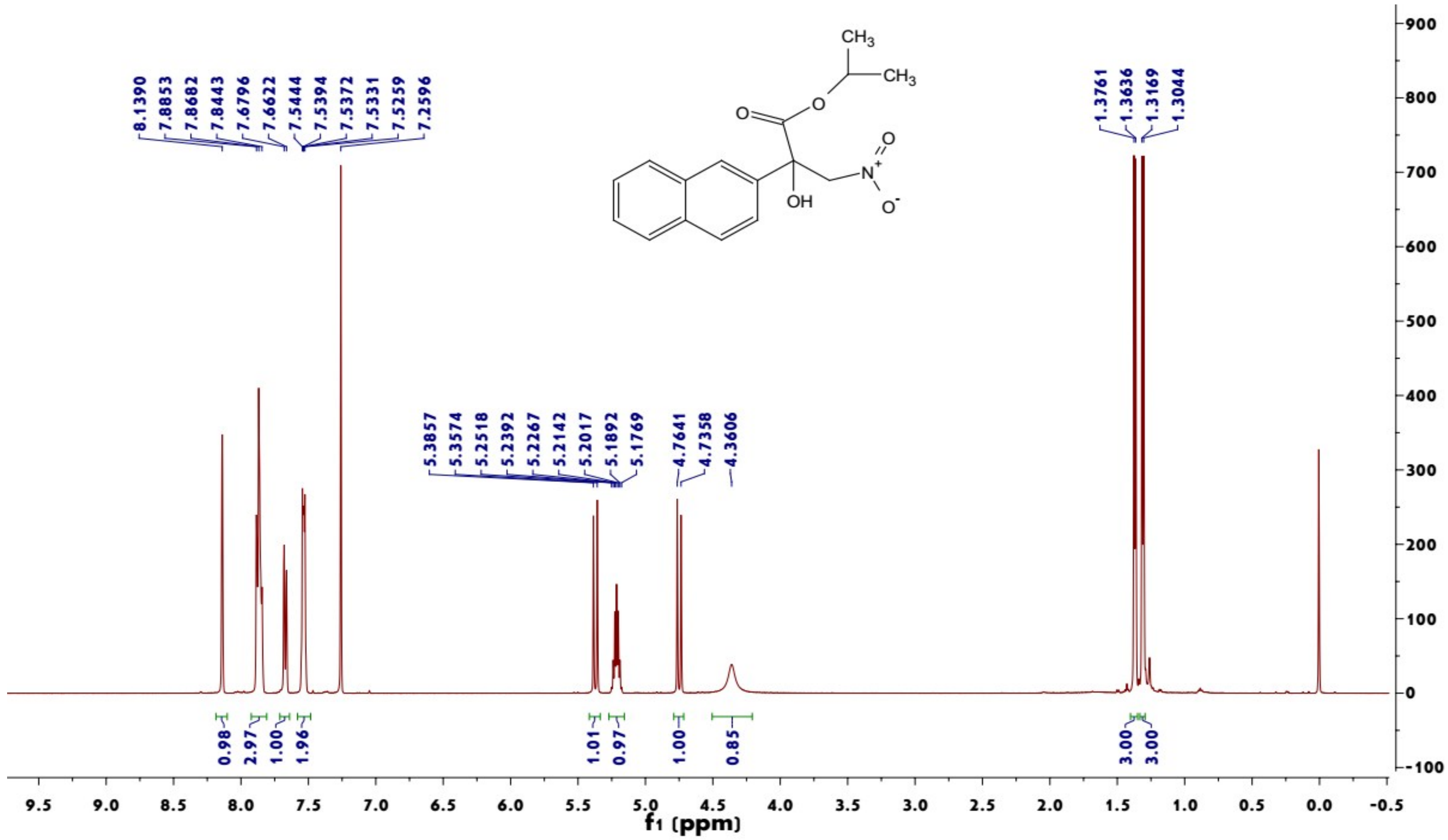

CH3
CH3
O
O
N+
O
O-
OH
8.1390
7.8853
7.8682
7.8443
7.6796
7.6622
7.5444
7.5394
7.5372
7.5331
7.5259
7.2596
5.3857
5.3574
5.2518
5.2392
5.2267
5.2142
5.2017
5.1892
5.1769
4.7641
4.7358
4.3606
1.3761
1.3636
1.3169
1.3044
0.98
2.97
1.00
1.96
1.01
0.97
1.00
0.85
3.00
3.00
f1 (ppm)

2fc

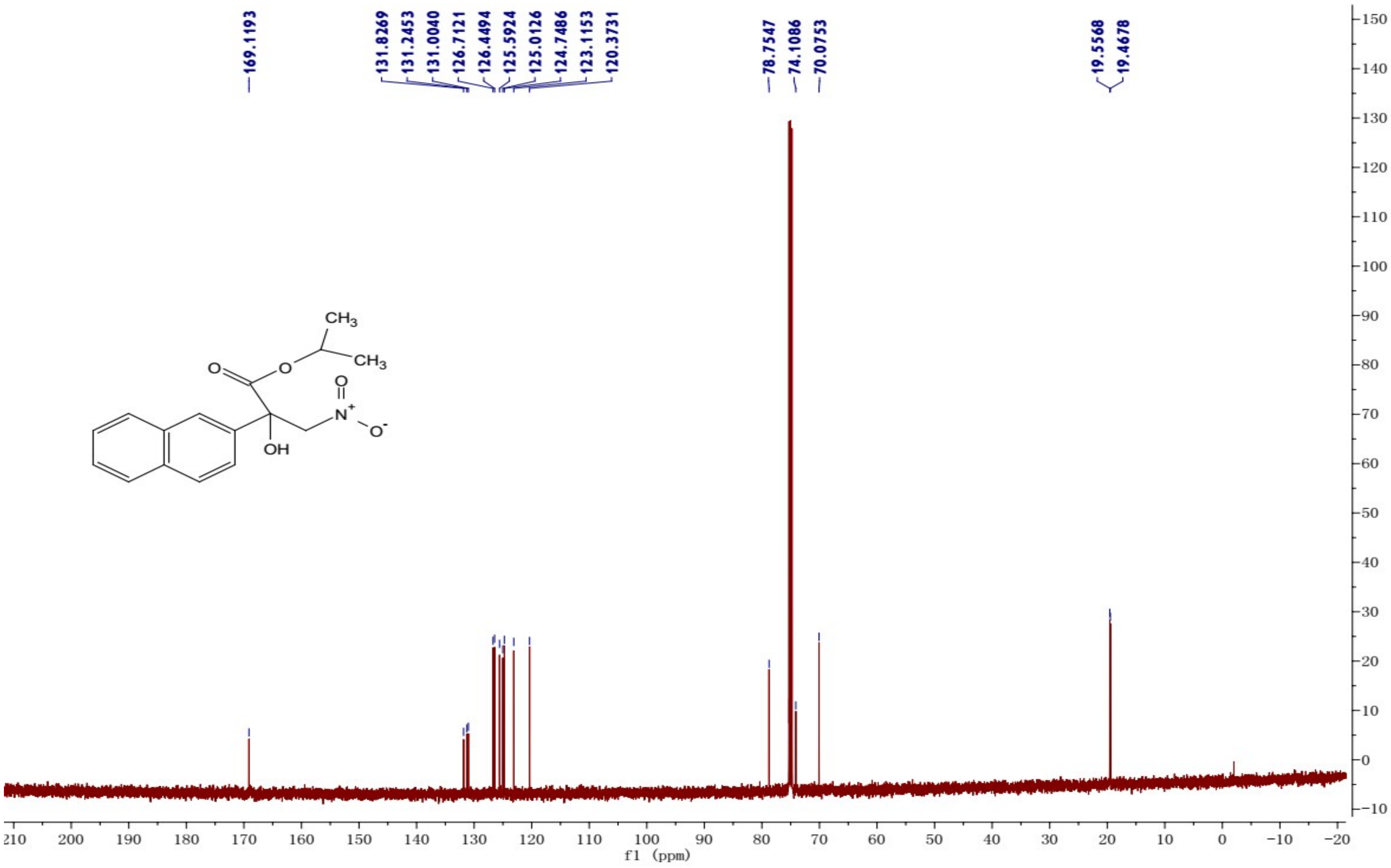

169.1193
131.8269
131.2453
131.0040
126.7121
126.4494
125.5924
125.0126
124.7486
123.1153
120.3731
78.7547
74.1086
70.0753
19.5568
19.4678
CH3
CH3
O
O
O
N+
O-
OH
f1 (ppm)

2gc

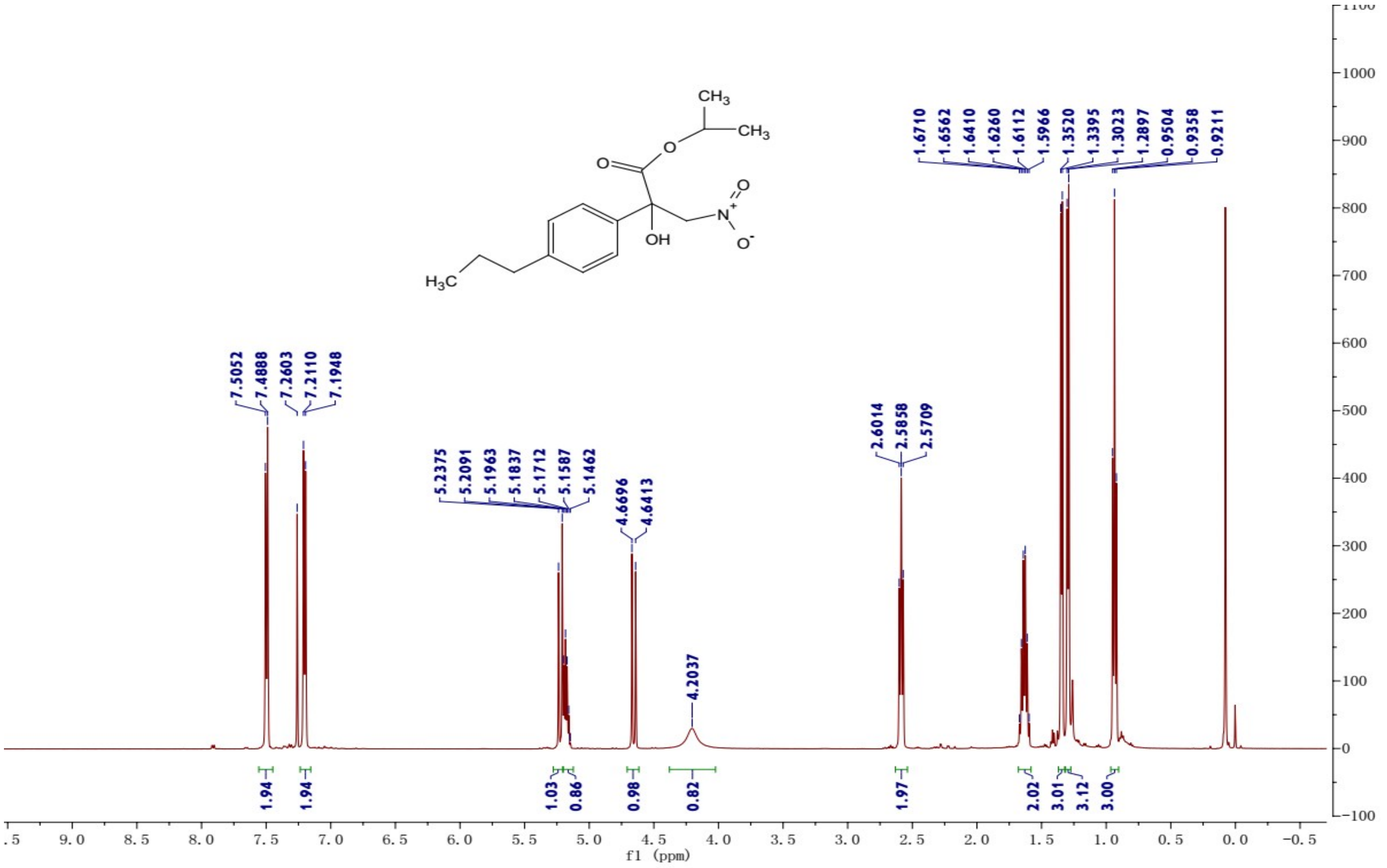
7.5052
7.4888
7.2603
7.2110
7.1948
5.2375
5.2091
5.1963
5.1837
5.1712
5.1587
5.1462
4.6696
4.6413
4.2037
2.6014
2.5858
2.5709
1.6710
1.6562
1.6410
1.6260
1.6112
1.5966
1.3520
1.3395
1.3023
1.2897
0.9504
0.9358
0.9211
1.94
1.94
1.03
0.86
0.98
0.82
1.97
2.02
3.01
3.12
3.00
f1 (ppm)

2gc

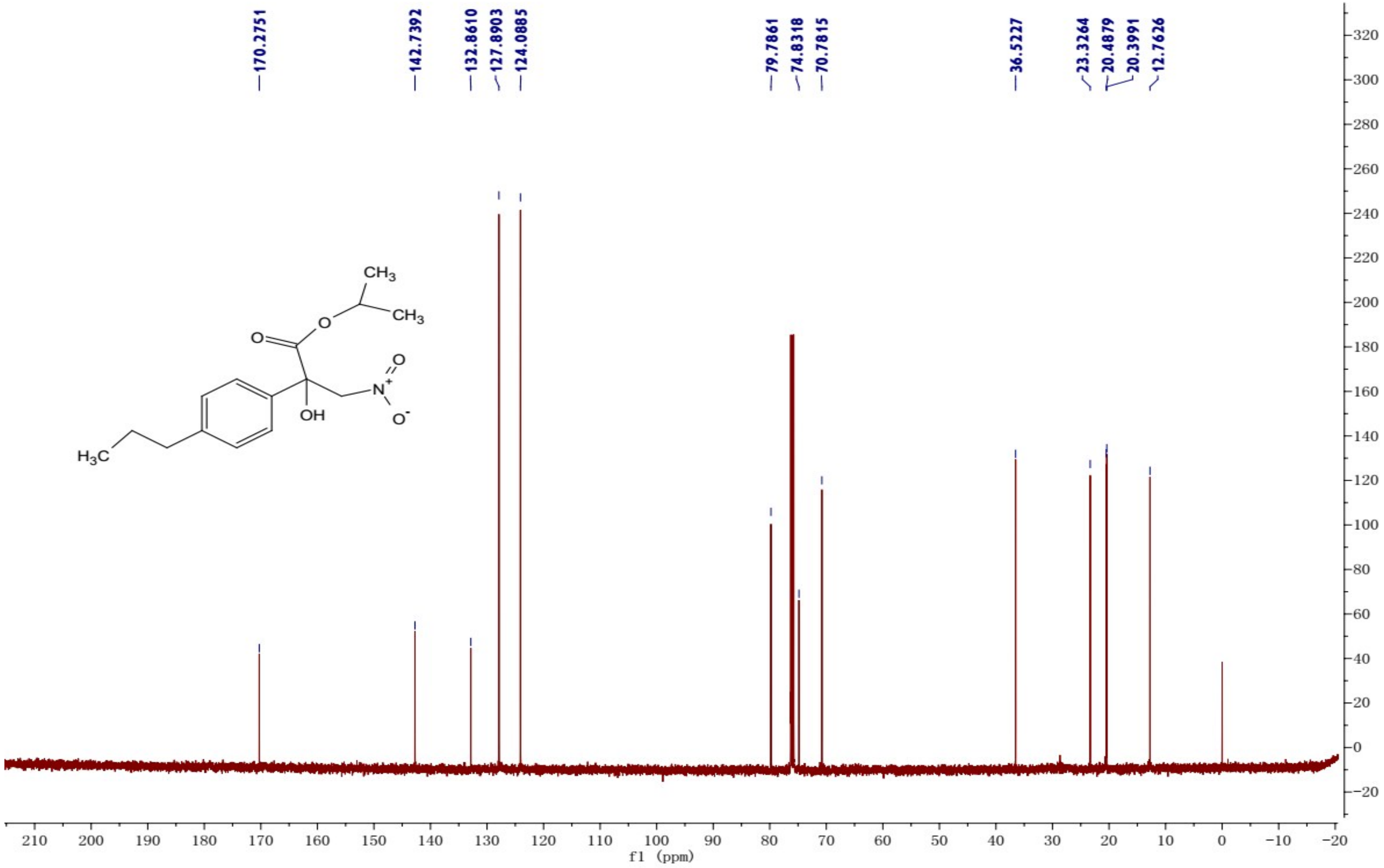
170.2751
142.7392
132.8610
127.8903
124.0885
79.7861
74.8318
70.7815
36.5227
23.3264
20.4879
20.3991
12.7626
f1 (ppm)
CH3
CH3
O
O
OH
N+
O
O-
H3C

2hc

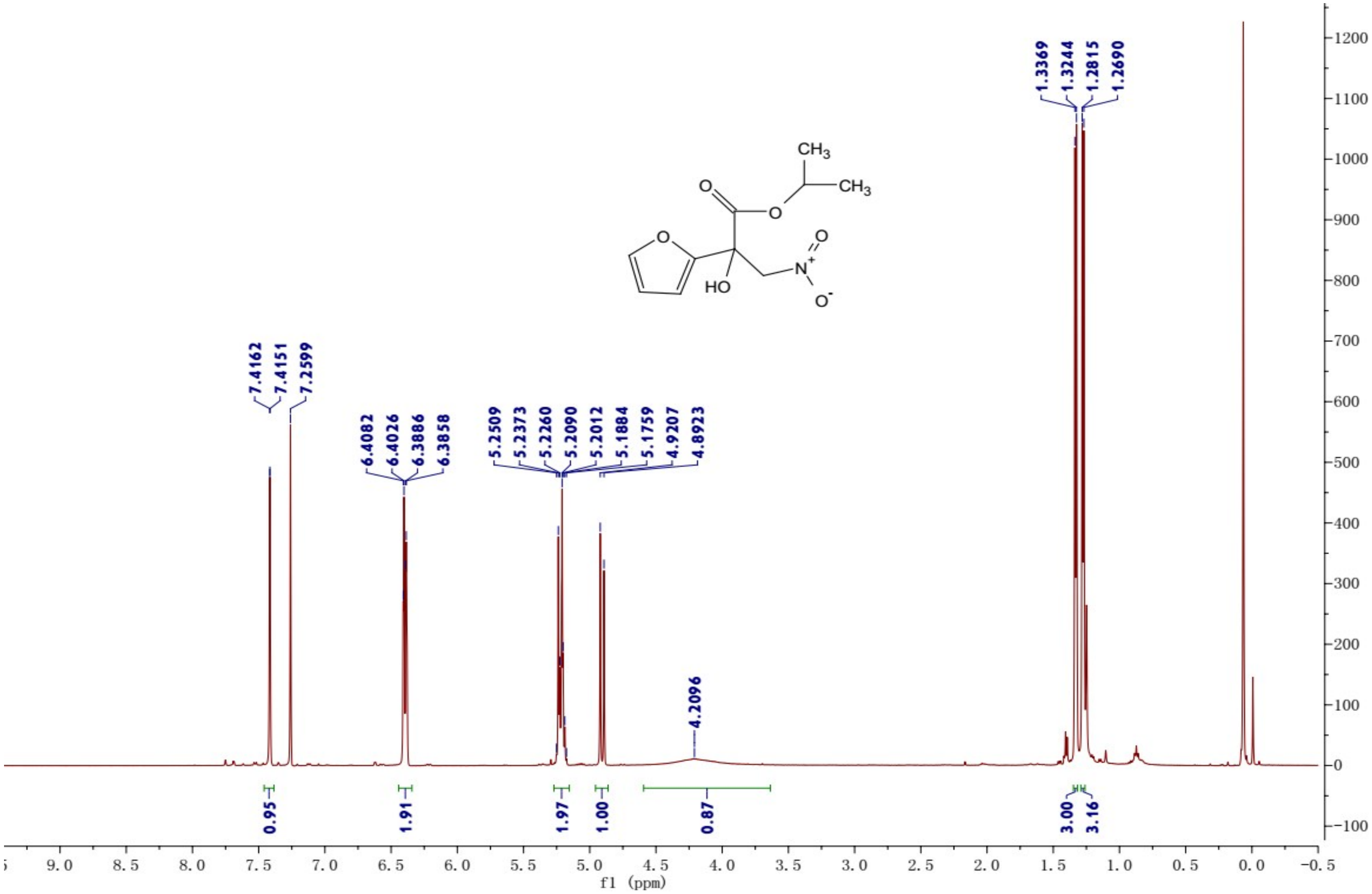
7.4162
7.4151
7.2599
6.4082
6.4026
6.3886
6.3858
5.2509
5.2373
5.2260
5.2090
5.2012
5.1884
5.1759
4.9207
4.8923
4.2096
1.3369
1.3244
1.2815
1.2690
0.95
1.91
1.97
1.00
0.87
3.00
3.16
f1 (ppm)

2hc

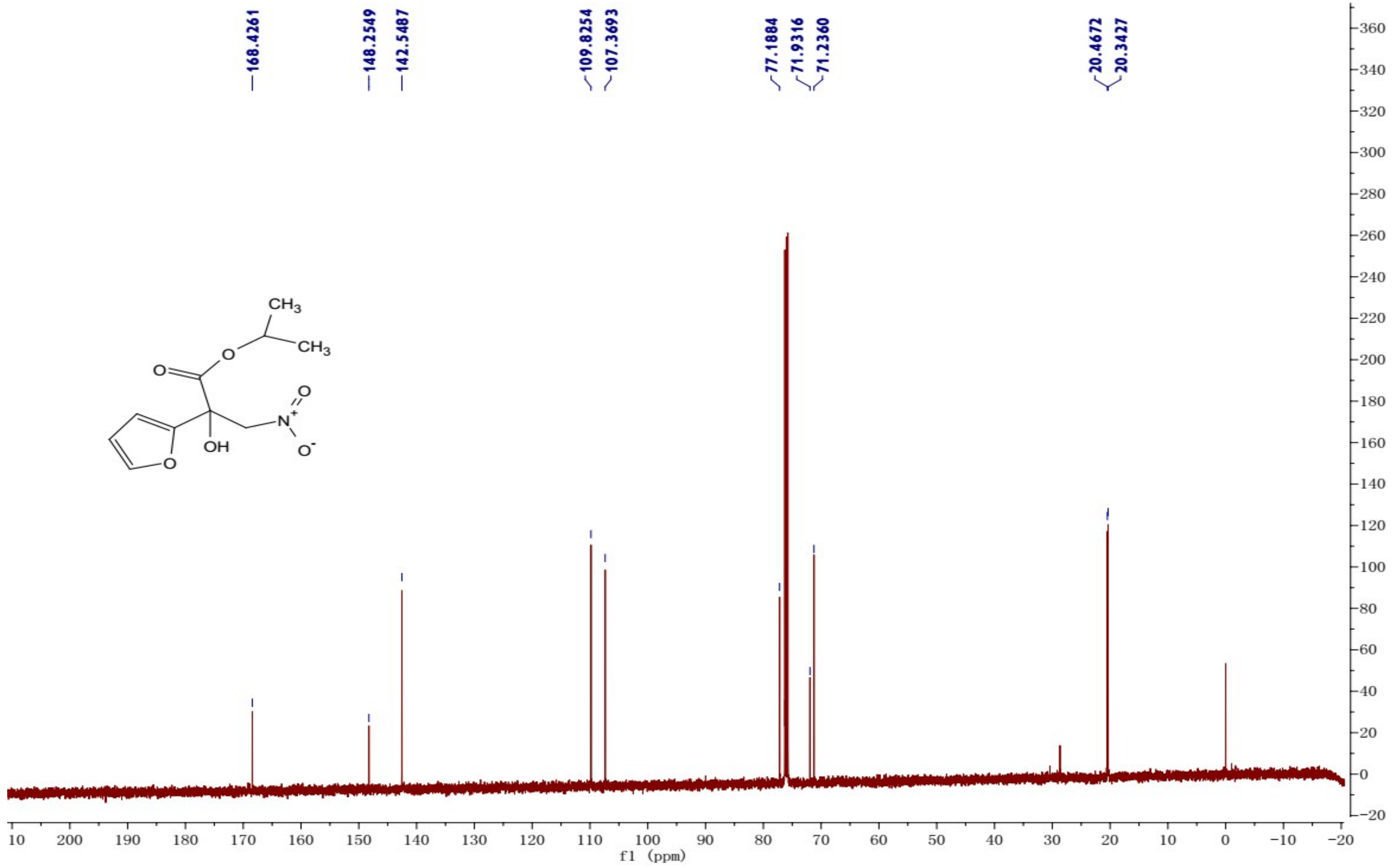

168.4261
148.2549
142.5487
109.8254
107.3693
77.1884
71.9316
71.2360
20.4672
20.3427
f1 (ppm)

2ic

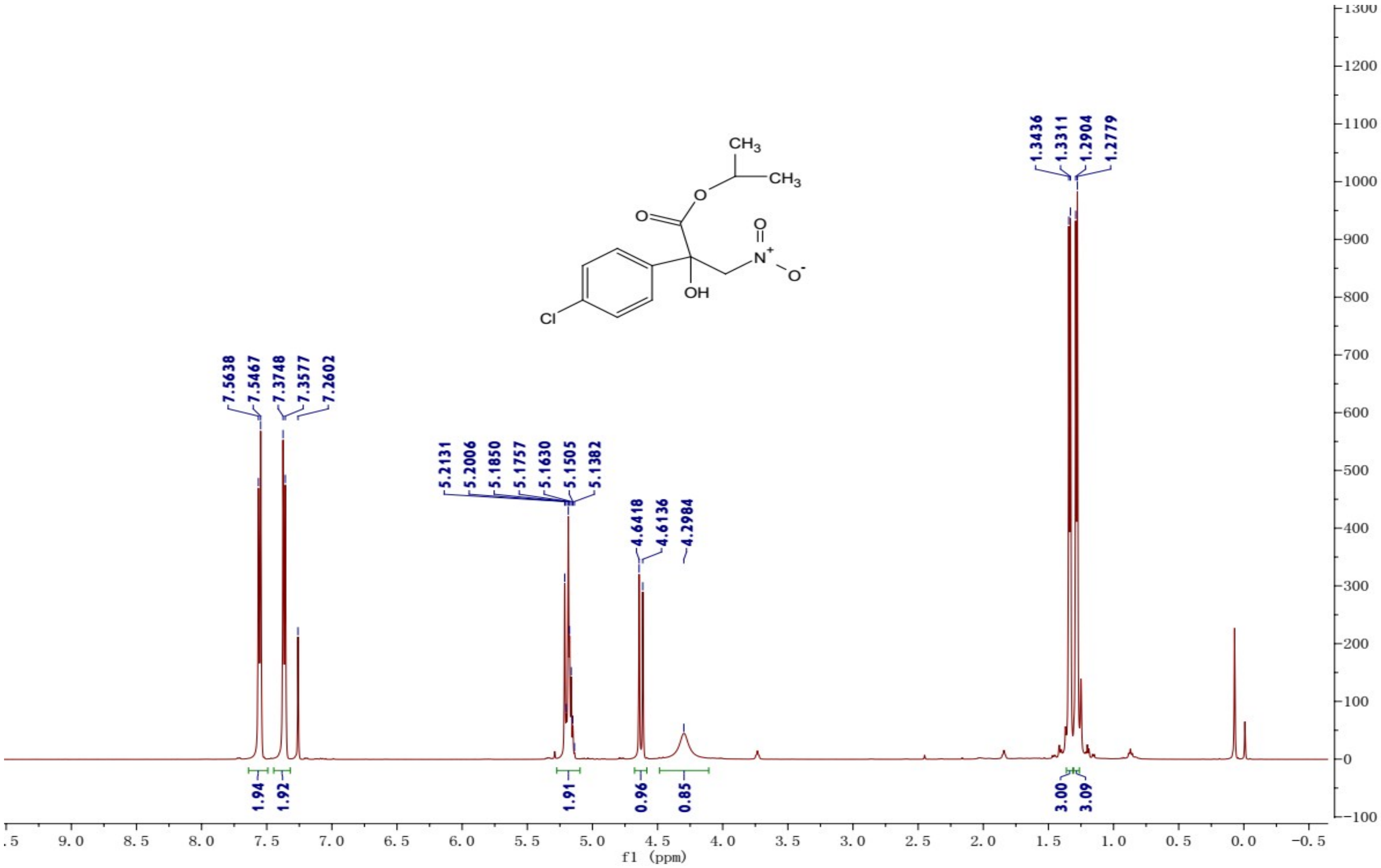

CH3
CH3
O
O
N+
O
O-
OH
Cl
7.5638
7.5467
7.3748
7.3577
7.2602
5.2131
5.2006
5.1850
5.1757
5.1630
5.1505
5.1382
4.6418
4.6136
4.2984
1.3436
1.3311
1.2904
1.2779
1.94
1.92
1.91
0.96
0.85
3.00
3.09
f1 (ppm)

2ic

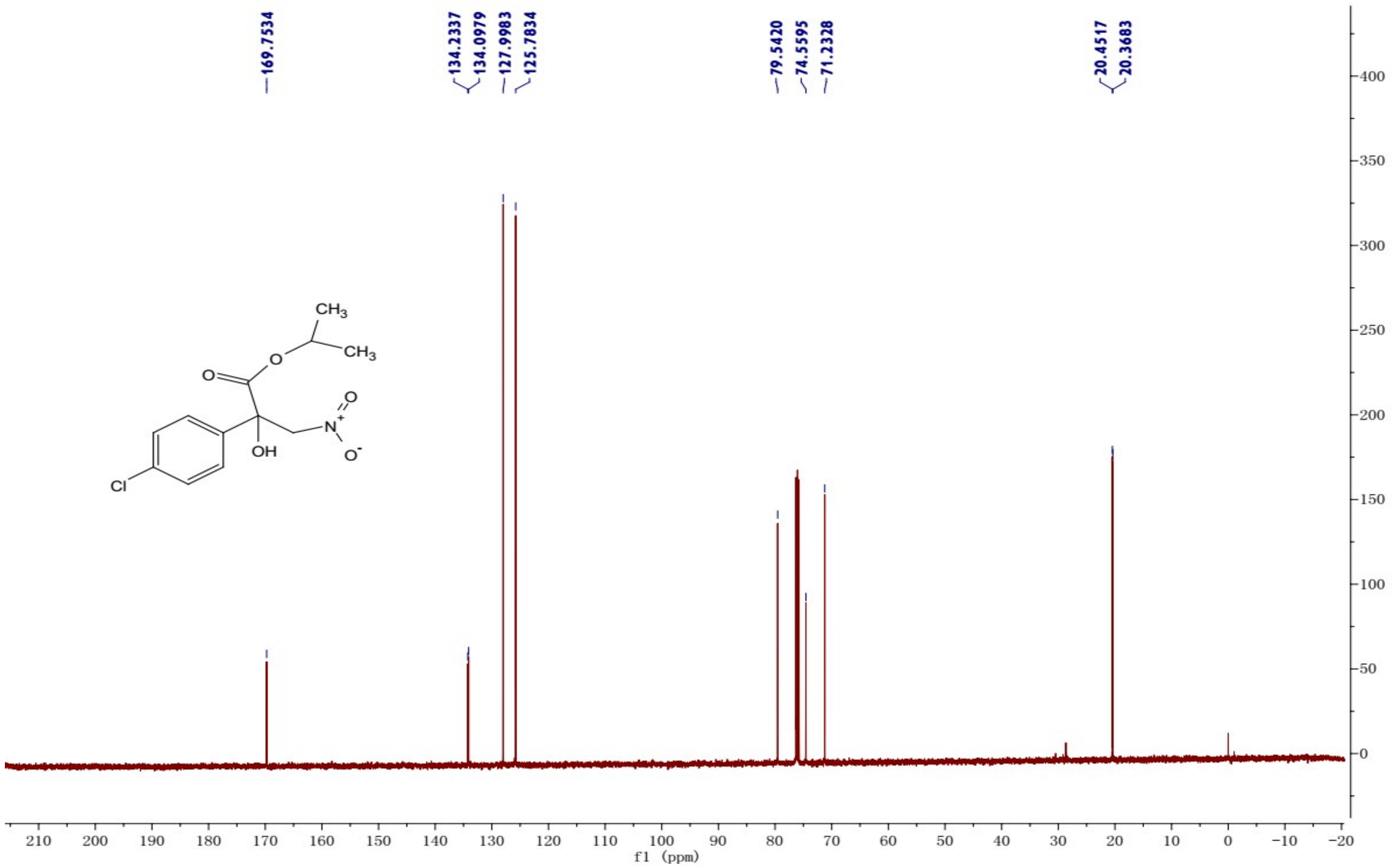
169.7534
134.2337
134.0979
127.9983
125.7834
79.5420
74.5595
71.2328
20.4517
20.3683
CH3
CH3
O
O
O
N+
O-
OH
Cl
f1 (ppm)

2jc

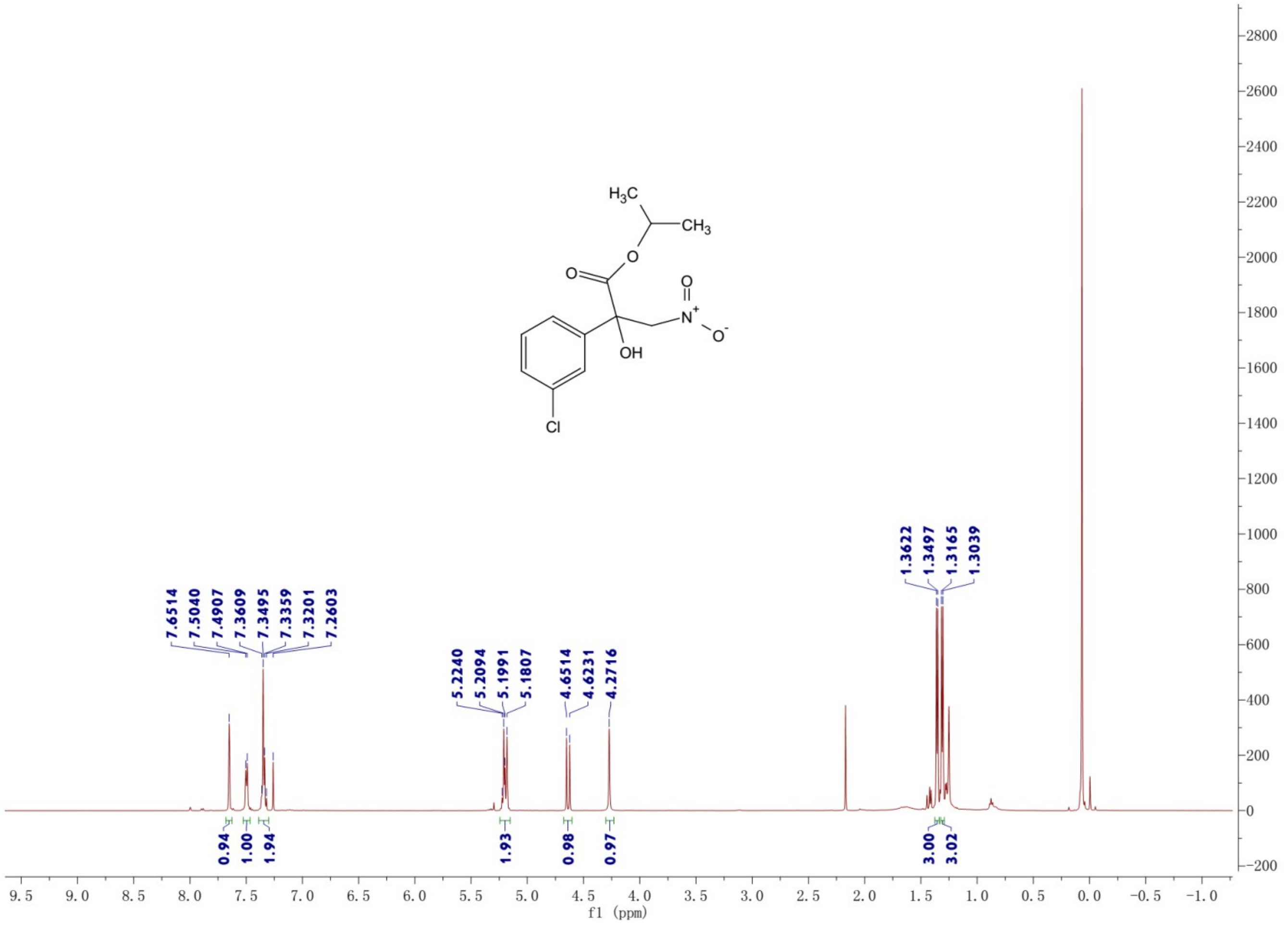
H3C
CH3
O
O
O
N+
O-
OH
Cl
7.6514
7.5040
7.4907
7.3609
7.3495
7.3359
7.3201
7.2603
5.2240
5.2094
5.1991
5.1807
4.6514
4.6231
4.2716
1.3622
1.3497
1.3165
1.3039
0.94
1.00
1.94
1.93
0.98
0.97
3.00
3.02
f1 (ppm)

2jc

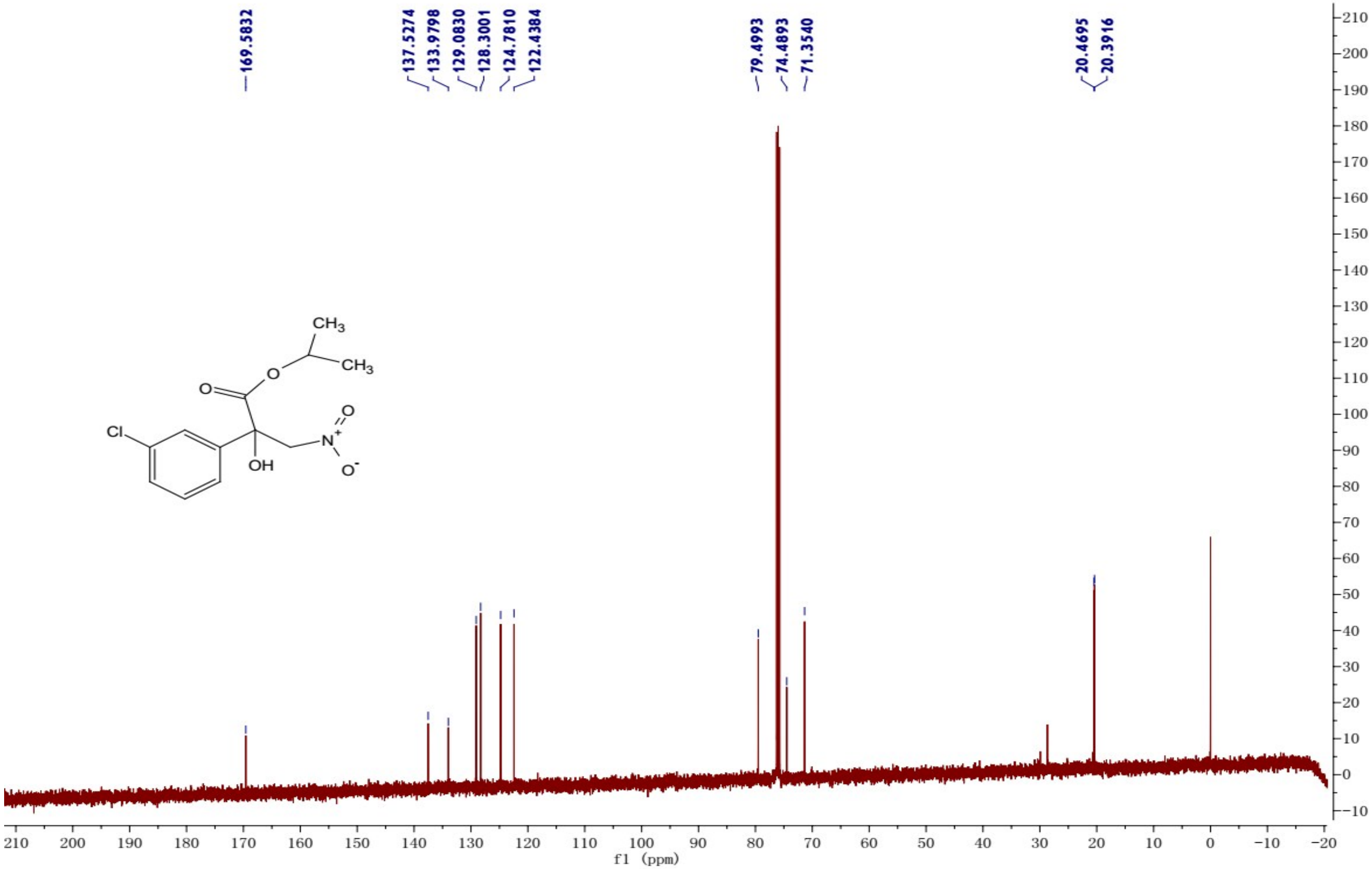

169.5832
137.5274
133.9798
129.0830
128.3001
124.7810
122.4384
79.4993
74.4893
71.3540
20.4695
20.3916
CH3
CH3
O
O
Cl
OH
N+
O
O-
f1 (ppm)

2kc

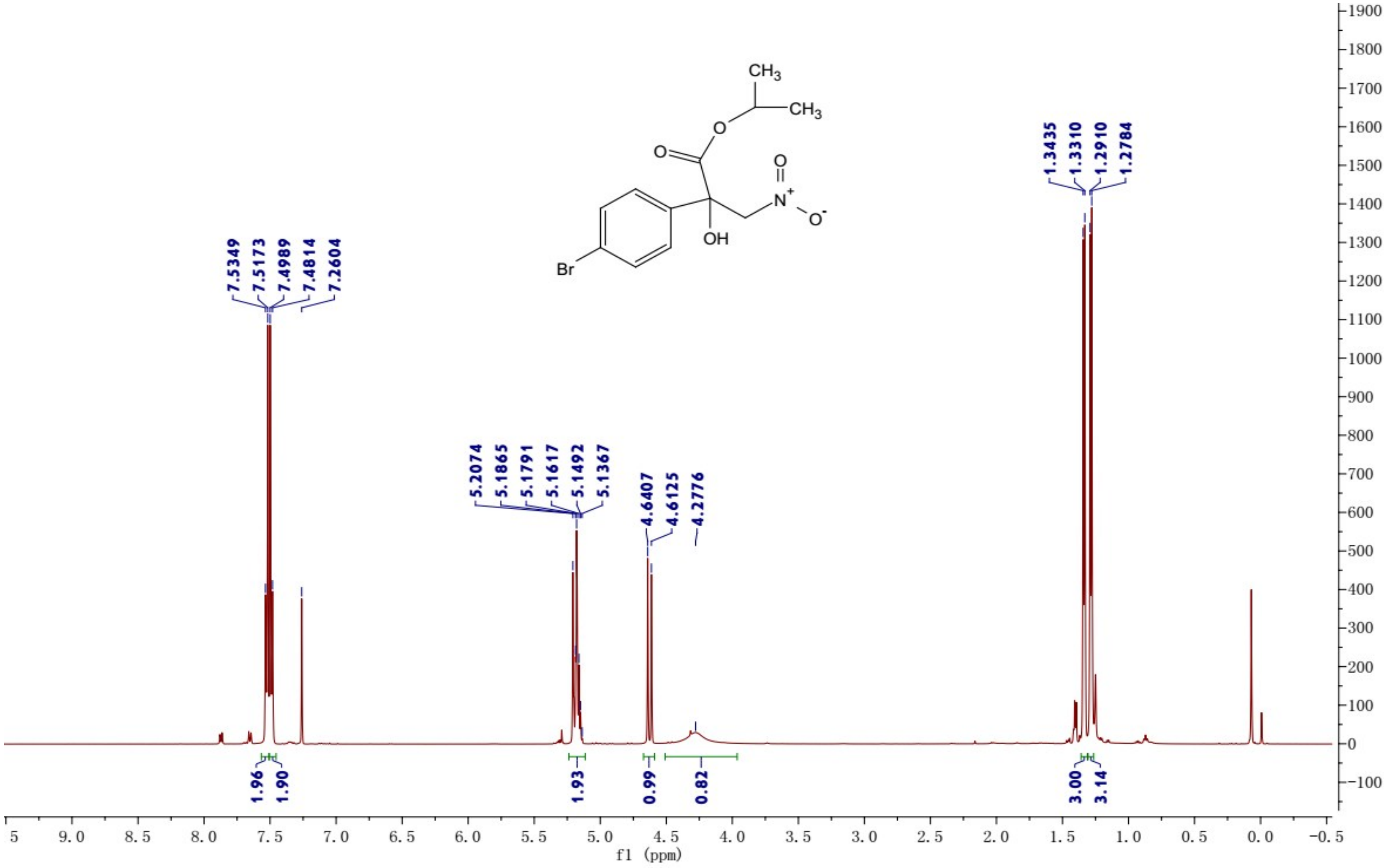

CH3
CH3
O
O
O
N+
O-
OH
Br
7.5349
7.5173
7.4989
7.4814
7.2604
5.2074
5.1865
5.1791
5.1617
5.1492
5.1367
4.6407
4.6125
4.2776
1.3435
1.3310
1.2910
1.2784
1.96
1.90
1.93
0.99
0.82
3.00
3.14
f1 (ppm)

2kc

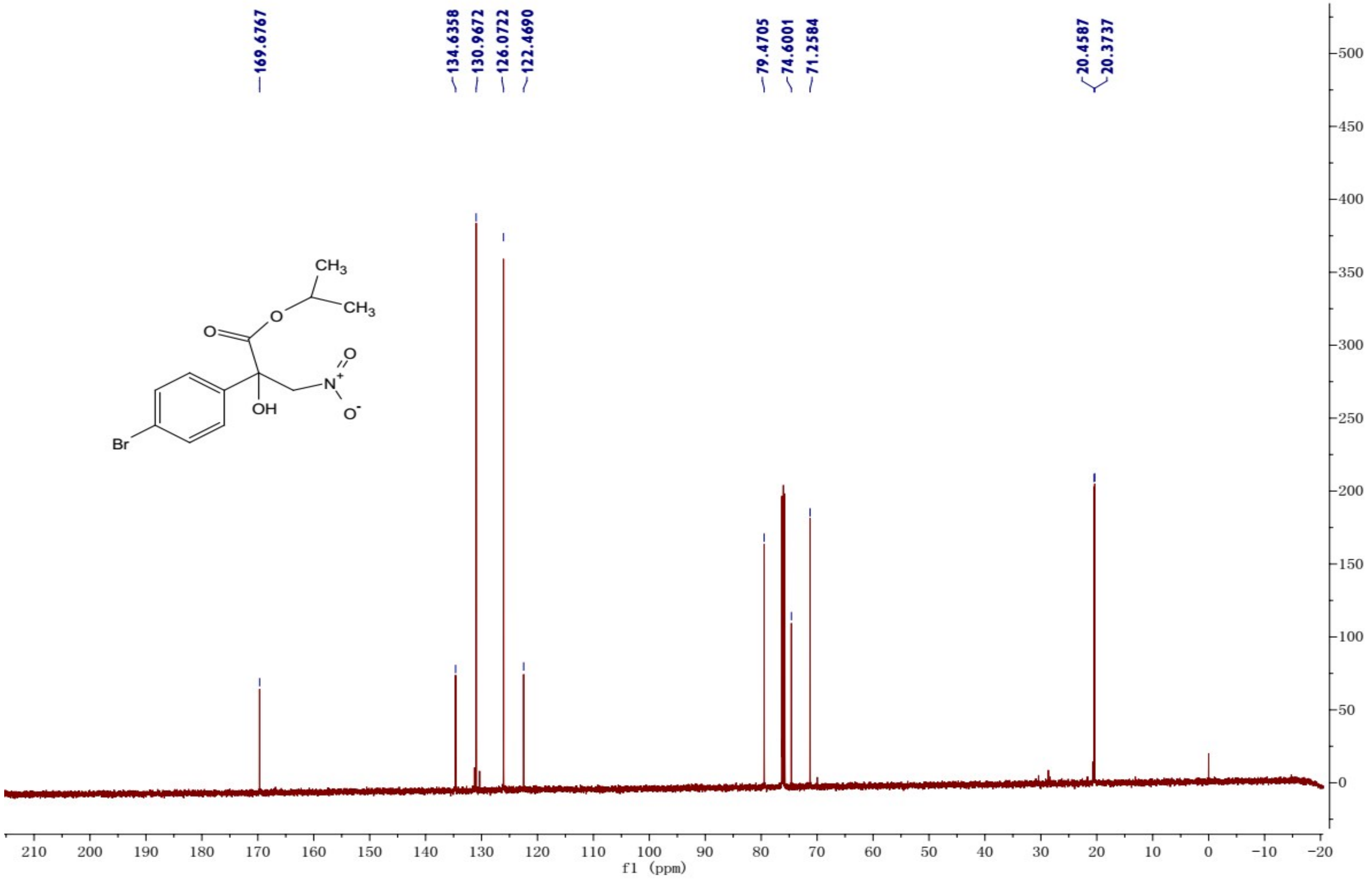
169.6767
134.6358
130.9672
126.0722
122.4690
79.4705
74.6001
71.2584
20.4587
20.3737
CH3
O
CH3
O
O
N+
O-
OH
Br
f1 (ppm)

2lc

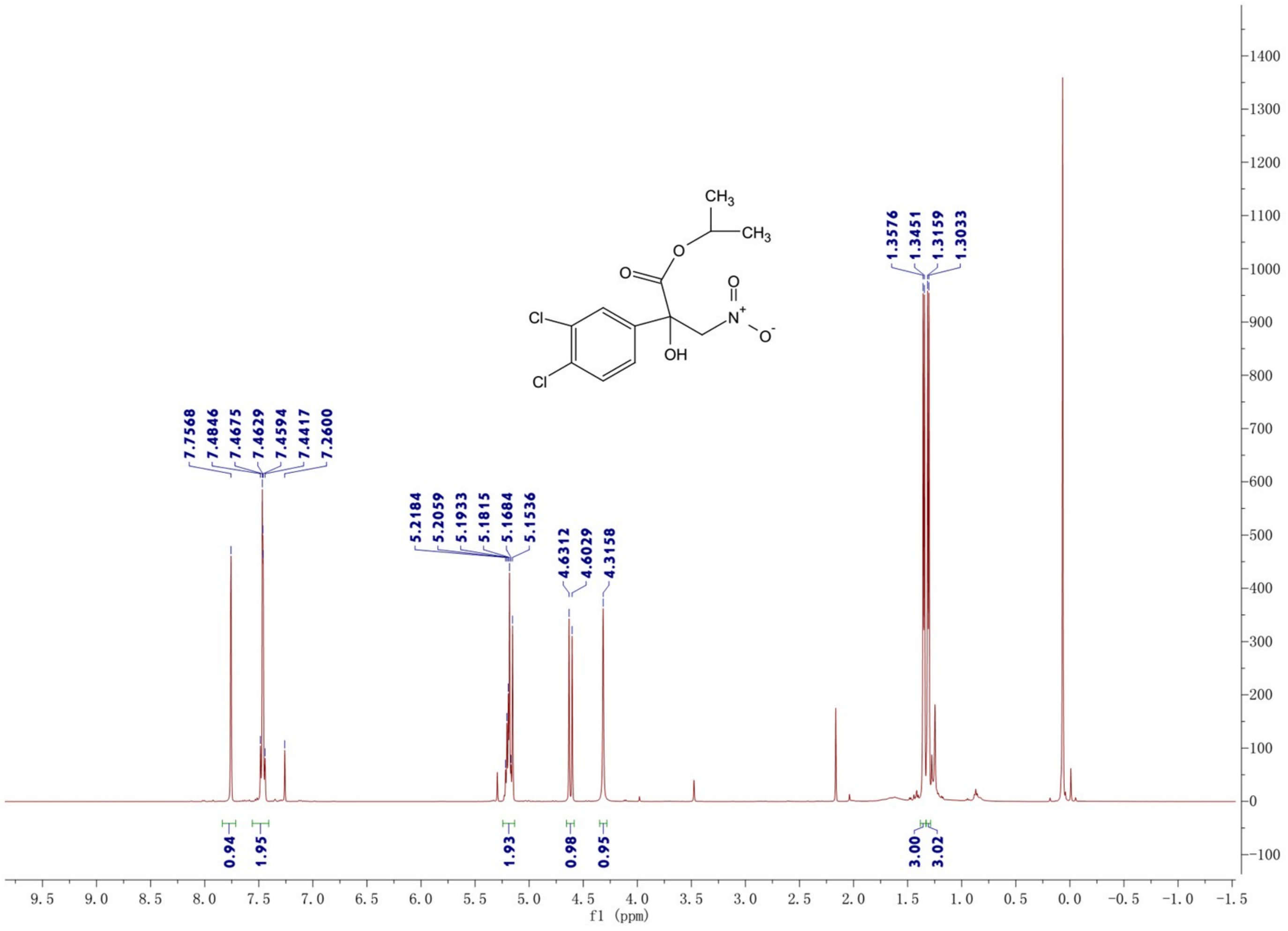
7.7568
7.4846
7.4675
7.4629
7.4594
7.4417
7.2600
5.2184
5.2059
5.1933
5.1815
5.1684
5.1536
4.6312
4.6029
4.3158
1.3576
1.3451
1.3159
1.3033
0.94
1.95
1.93
0.98
0.95
3.00
3.02
f1 (ppm)

2lc

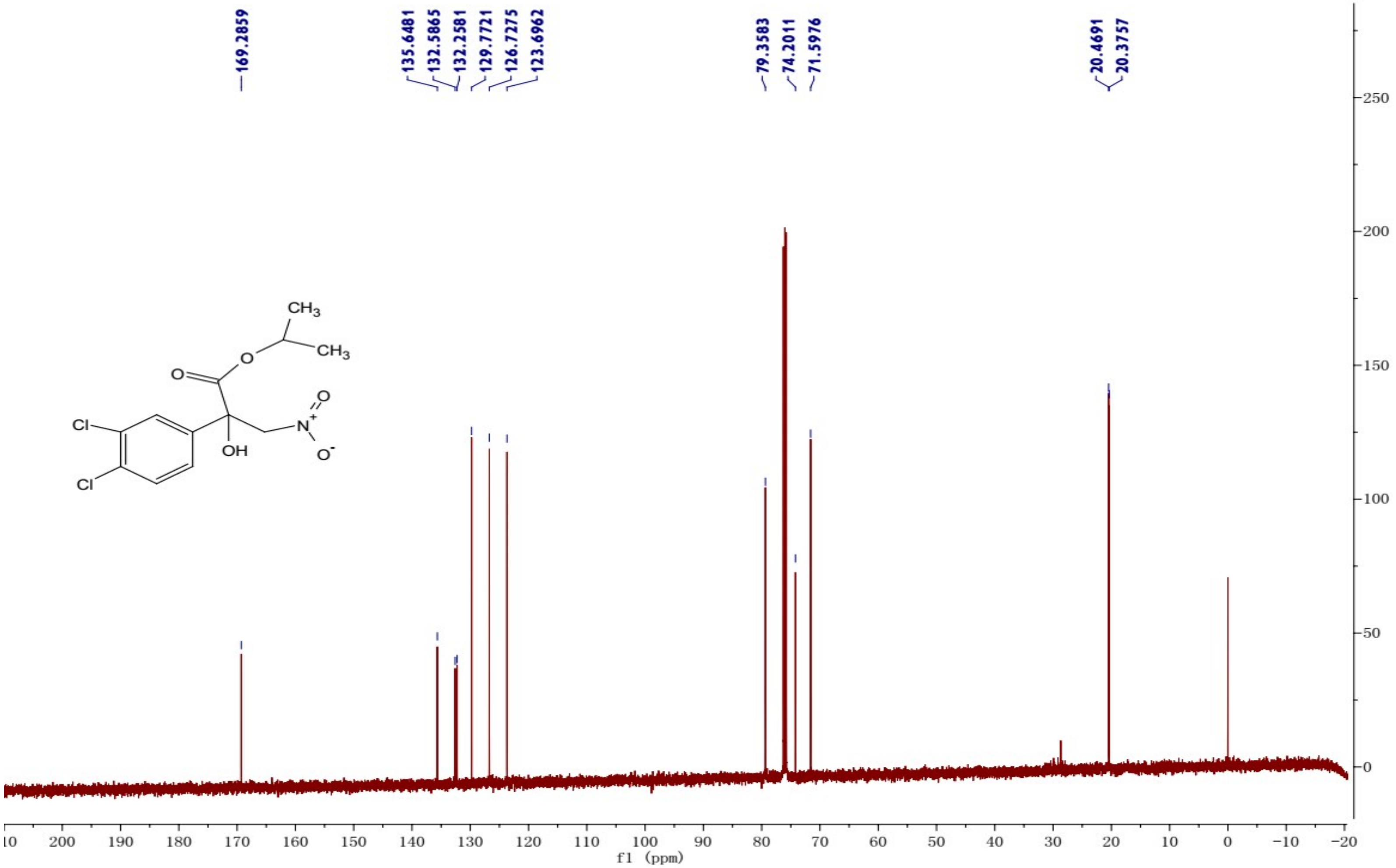

169.2859
135.6481
132.5865
132.2581
129.7721
126.7275
123.6962
79.3583
74.2011
71.5976
20.4691
20.3757
CH3
CH3
O
O
Cl
N
O
OH
Cl
O-
250
200
150
100
50
0
210
200
190
180
170
160
150
140
130
120
110
100
90
80
70
60
50
40
30
20
10
0
−10
−20
f1 (ppm)

2mc

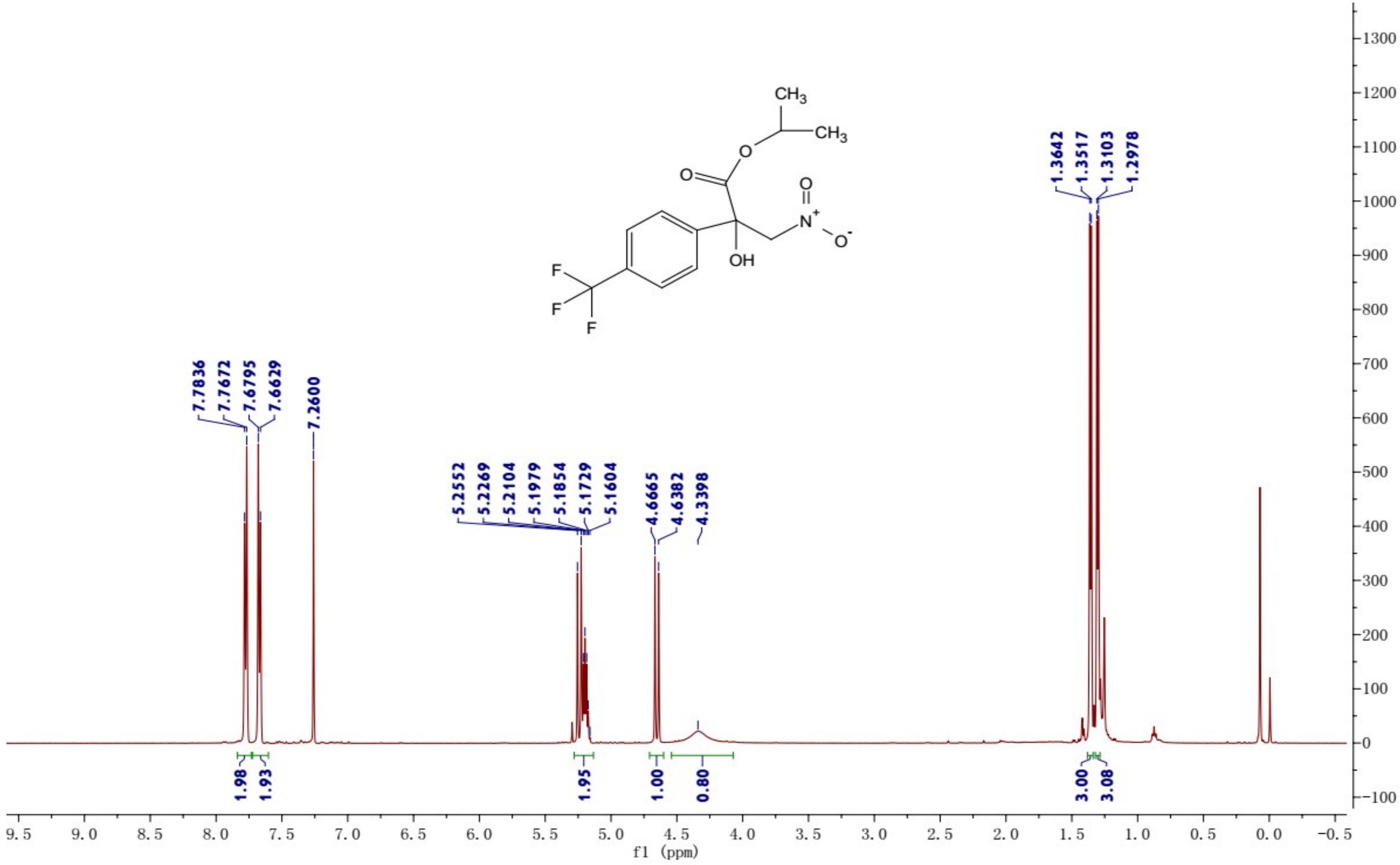

7.7836
7.7672
7.6795
7.6629
7.2600
5.2552
5.2269
5.2104
5.1979
5.1854
5.1729
5.1604
4.6665
4.6382
4.3398
1.3642
1.3517
1.3103
1.2978
1.98
1.93
1.95
1.00
0.80
3.00
3.08
f1 (ppm)
CH3
CH3
O
O
N+
O
O-
OH
F
F
F

2mc

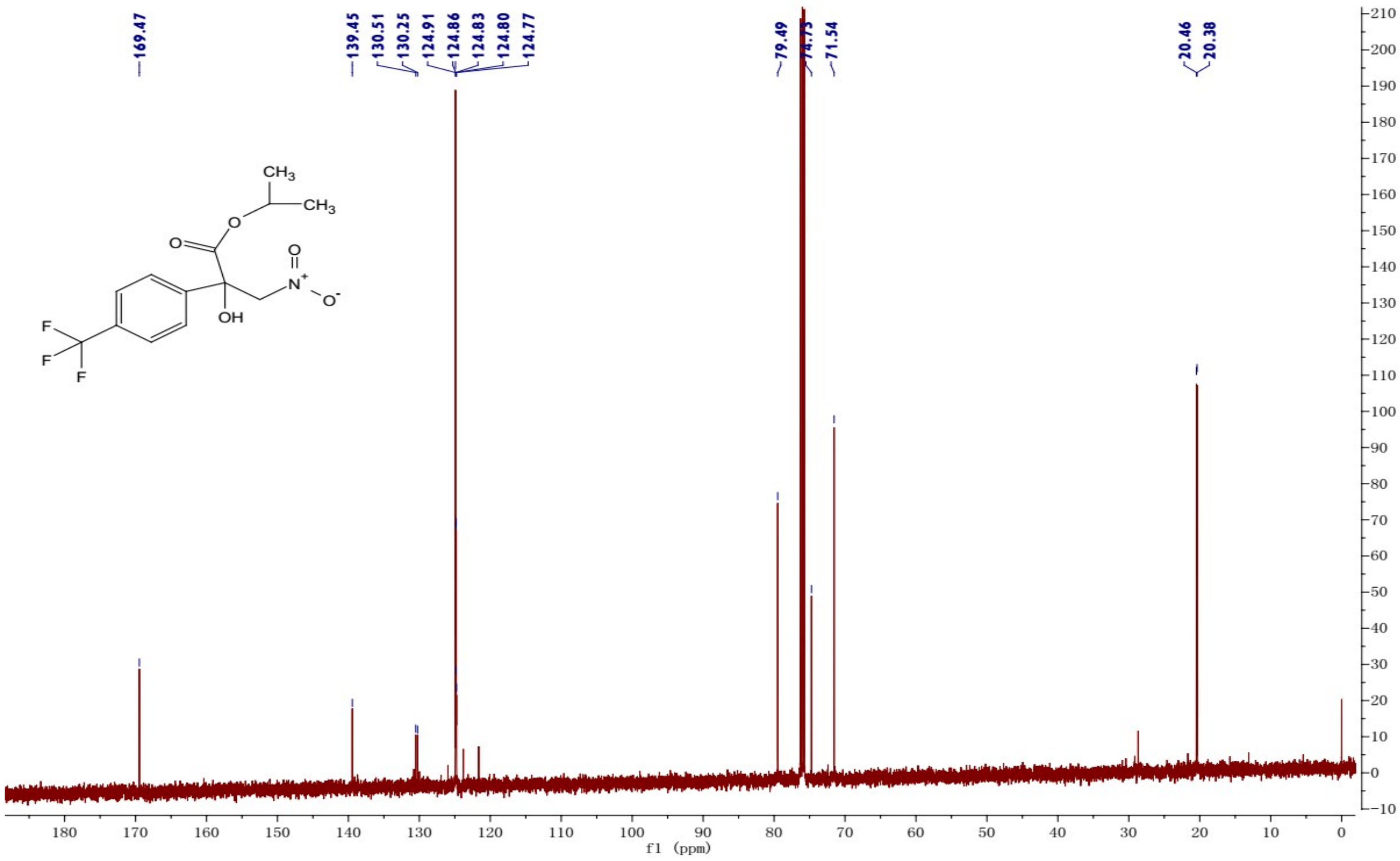

169.47
139.45
130.51
130.25
124.91
124.86
124.83
124.80
124.77
79.49
71.54
20.46
20.38
f1 (ppm)

2nb

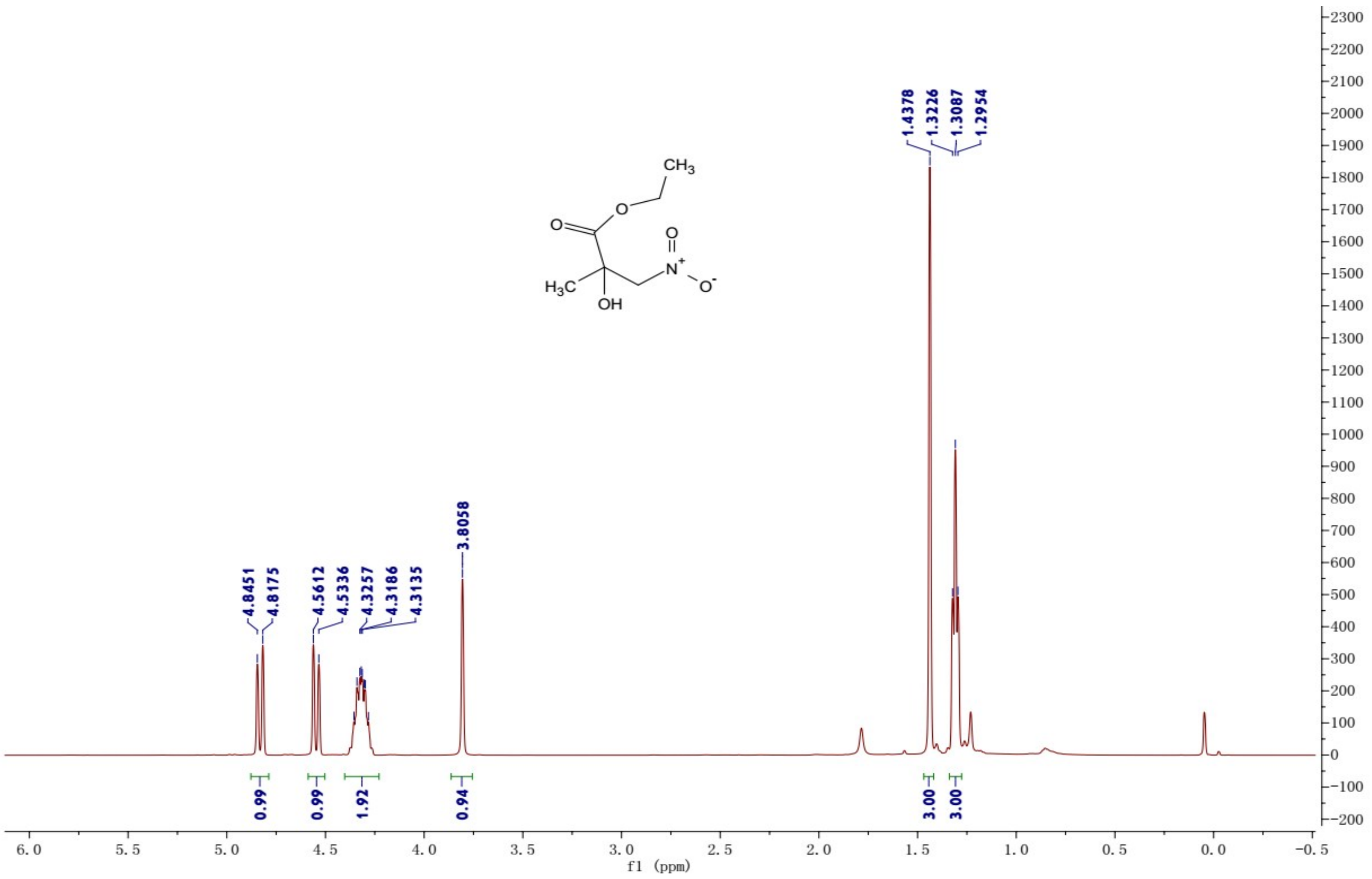

4.8451
4.8175
4.5612
4.5336
4.3257
4.3186
4.3135
3.8058
1.4378
1.3226
1.3087
1.2954
0.99
0.99
1.92
0.94
3.00
3.00
f1 (ppm)

2nb

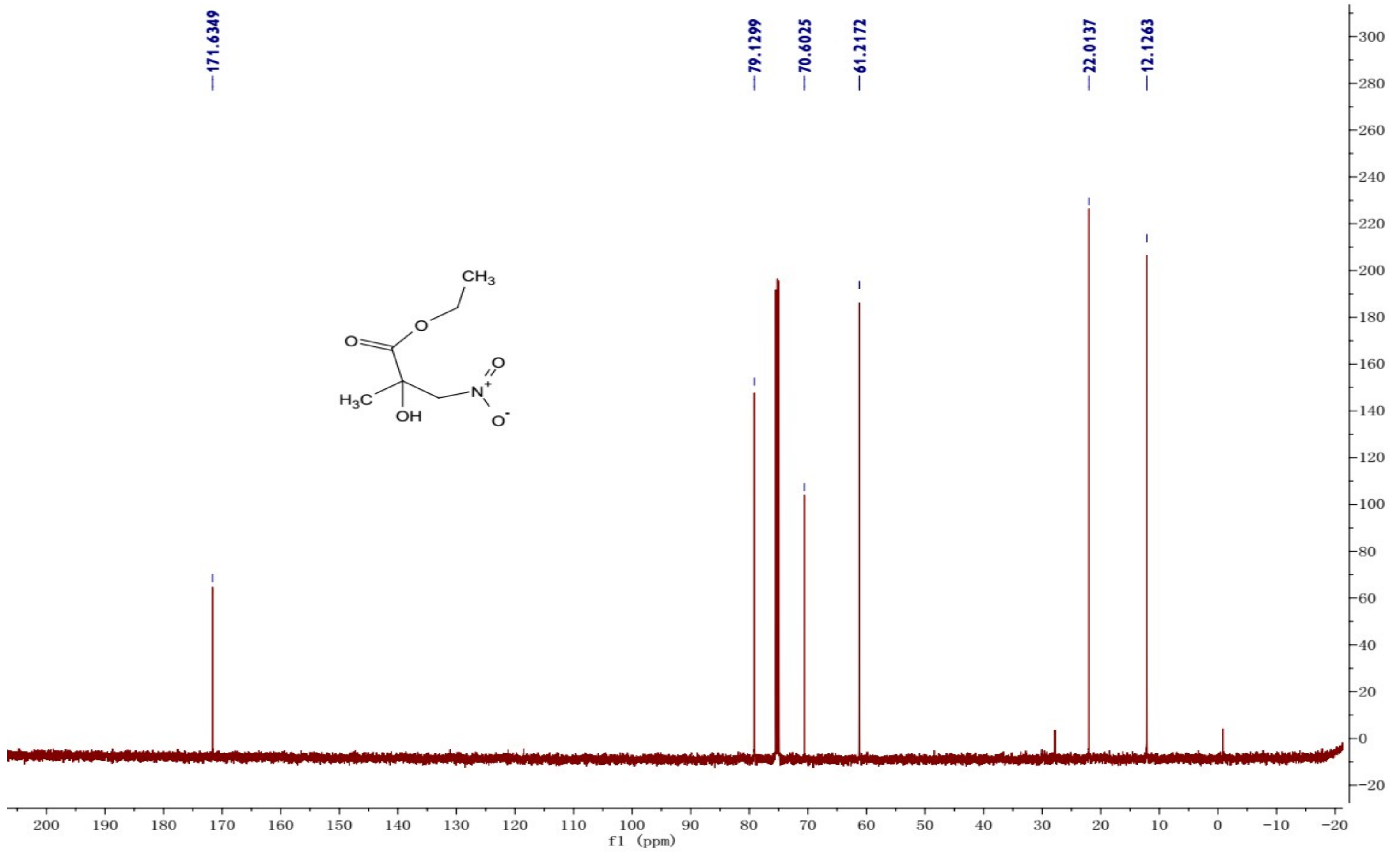

171.6349
79.1299
70.6025
61.2172
22.0137
12.1263
CH3
O
O
O
N+
O-
H3C
OH
f1 (ppm)

2aa

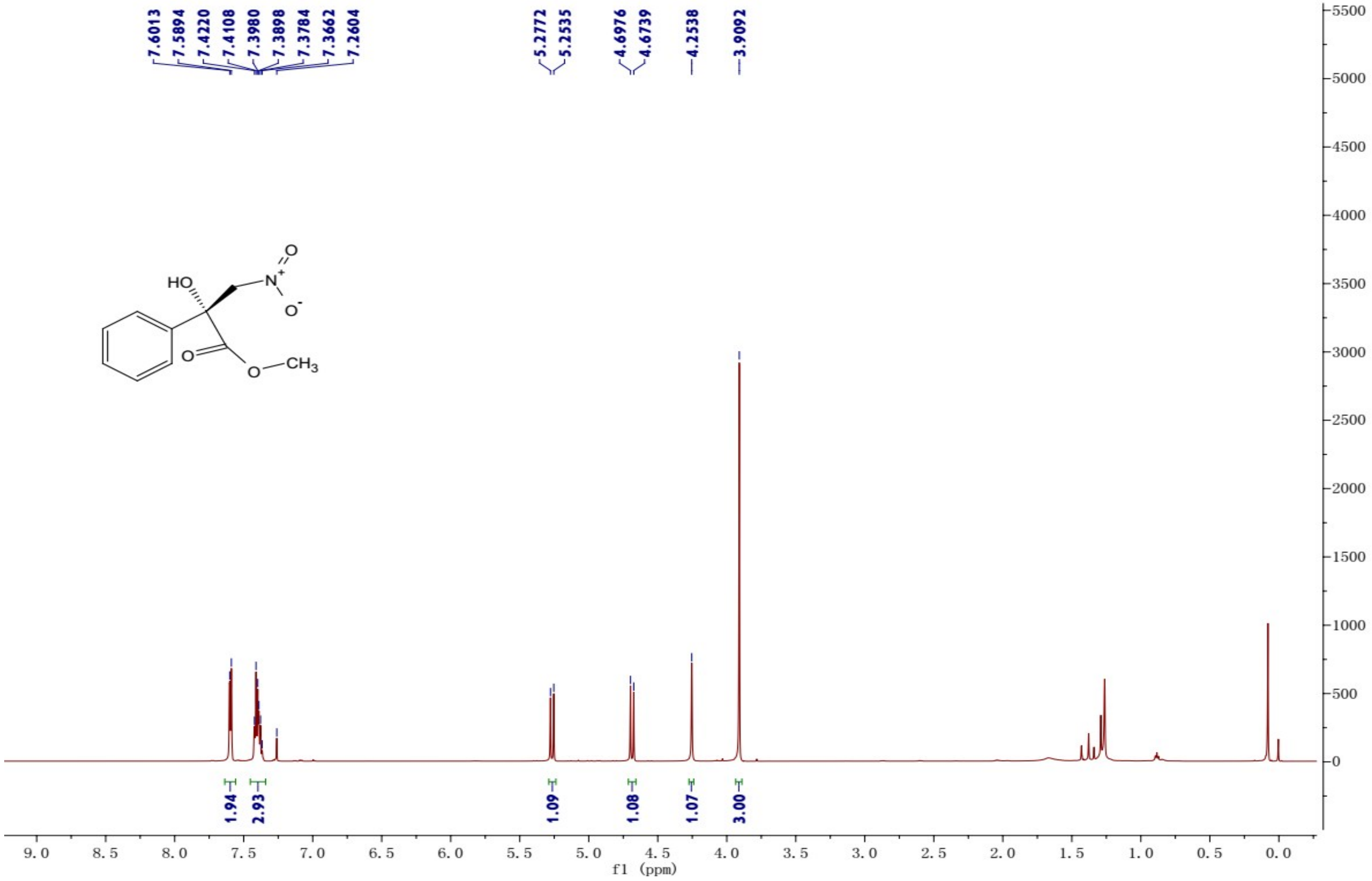

7.6013
7.5894
7.4220
7.4108
7.3980
7.3898
7.3784
7.3662
7.2604
5.2772
5.2535
4.6976
4.6739
4.2538
3.9092
HO
N
O
O
O
O
CH3
1.94
2.93
1.09
1.08
1.07
3.00
f1 (ppm)

2ab

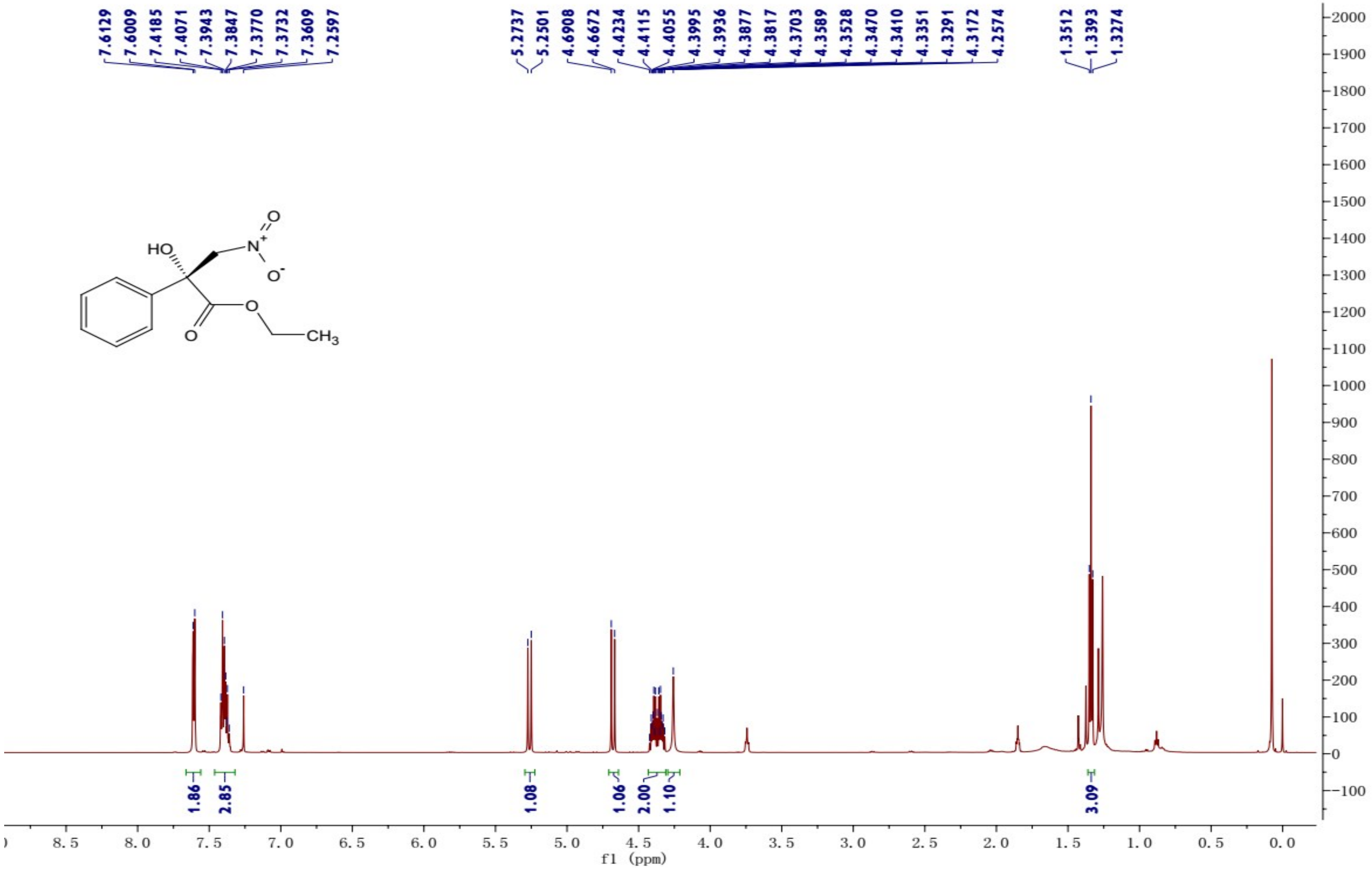
7.6129
7.6009
7.4185
7.4071
7.3943
7.3847
7.3770
7.3732
7.3609
7.2597
5.2737
5.2501
4.6908
4.6672
4.4234
4.4115
4.4055
4.3995
4.3936
4.3877
4.3817
4.3703
4.3589
4.3528
4.3470
4.3410
4.3351
4.3291
4.3172
4.2574
1.3512
1.3393
1.3274
HO
N
O
O
O
O
CH3
1.86
2.85
1.08
1.06
2.00
1.10
3.09
f1 (ppm)

4a

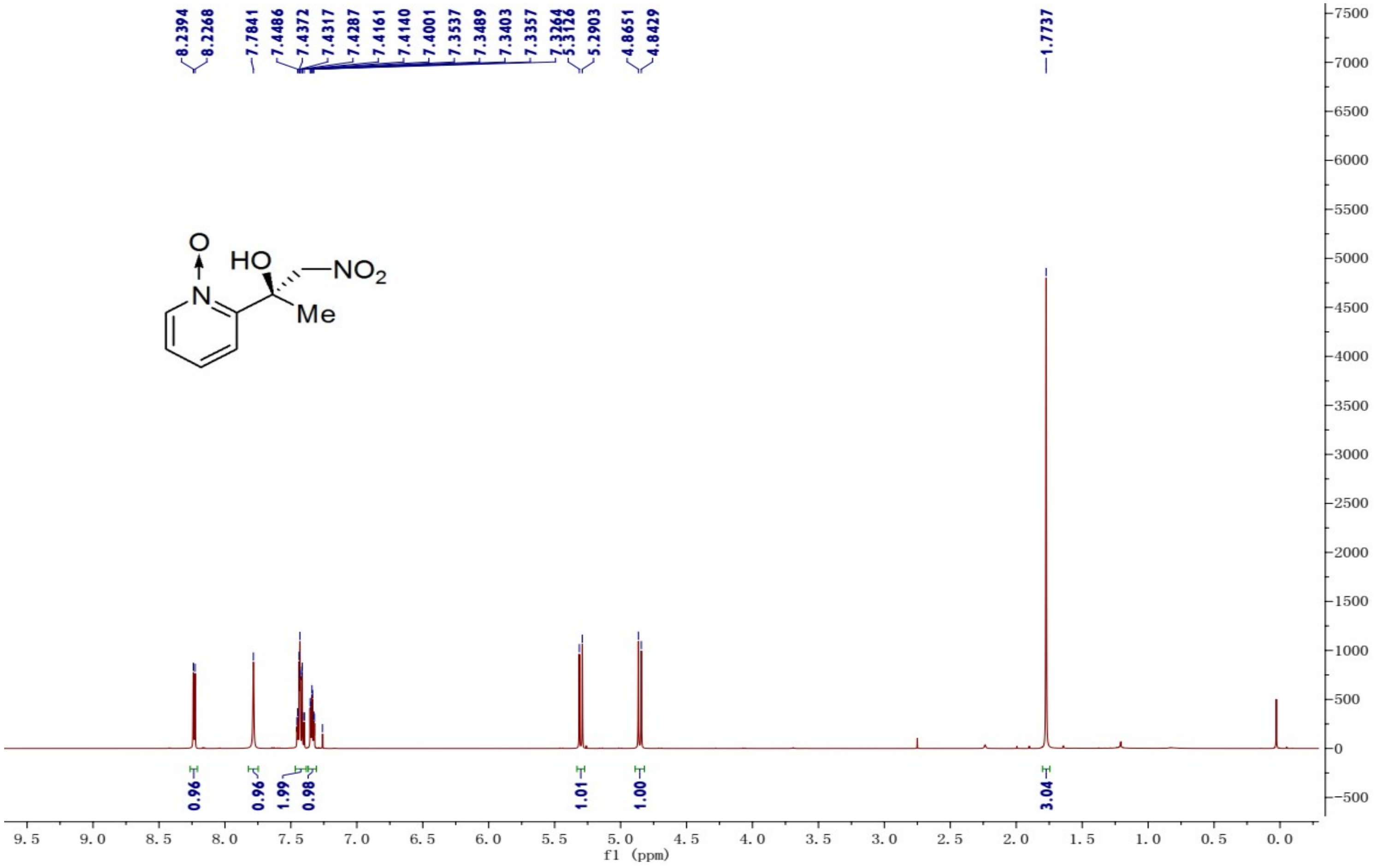

8.2394
8.2268
7.7841
7.4486
7.4372
7.4317
7.4287
7.4161
7.4140
7.4001
7.3537
7.3489
7.3403
7.3357
7.3264
5.3126
5.2903
4.8651
4.8429
1.7737
HO
NO2
N
O
Me
0.96
0.96
1.99
0.98
1.01
1.00
3.04
f1 (ppm)

4b

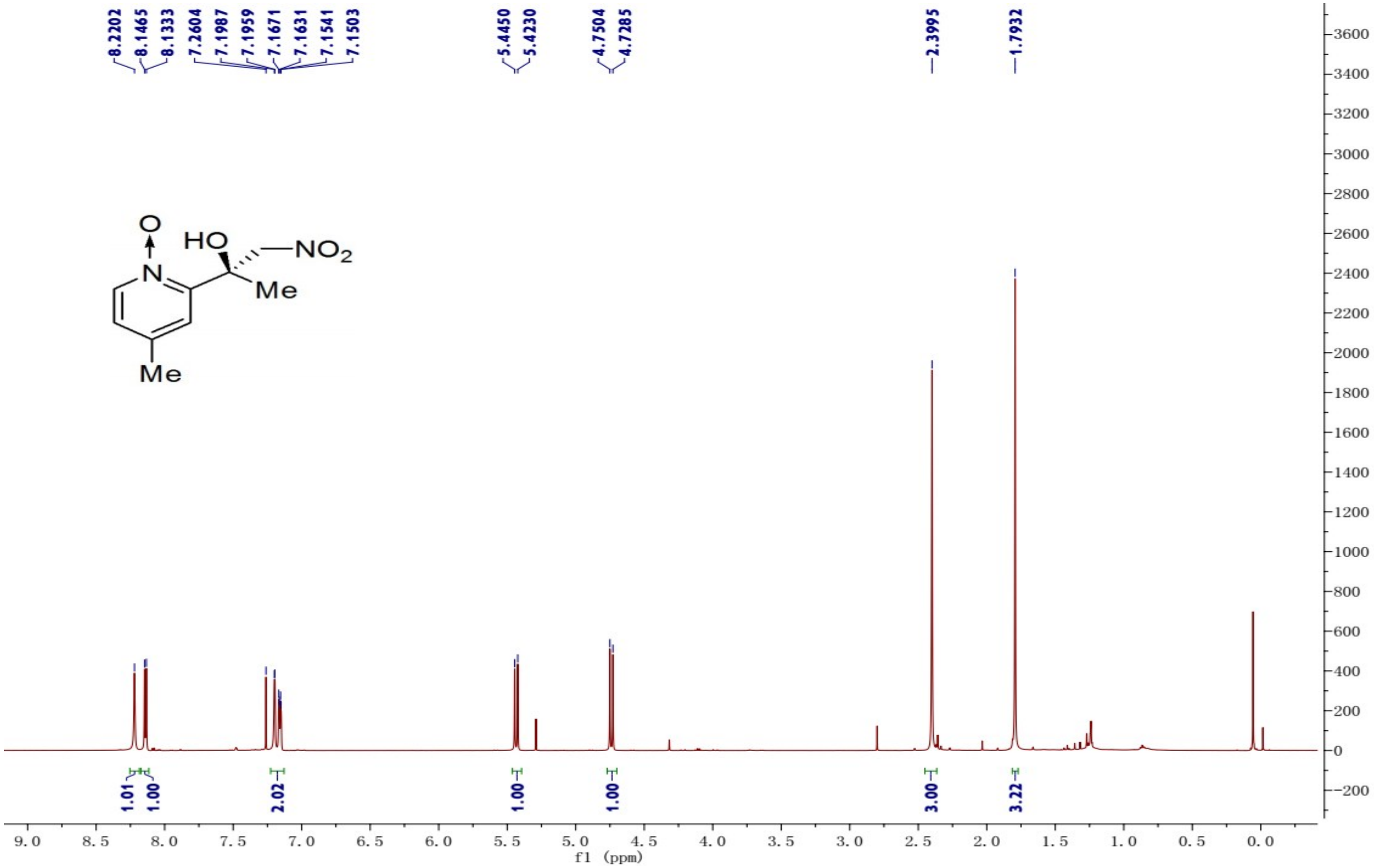
8.2202
8.1465
8.1333
7.2604
7.1987
7.1959
7.1671
7.1631
7.1541
7.1503
5.4450
5.4230
4.7504
4.7285
2.3995
1.7932
HO
NO_2
N
O
Me
Me
1.01
1.00
2.02
1.00
1.00
3.00
3.22
f1 (ppm)

4c

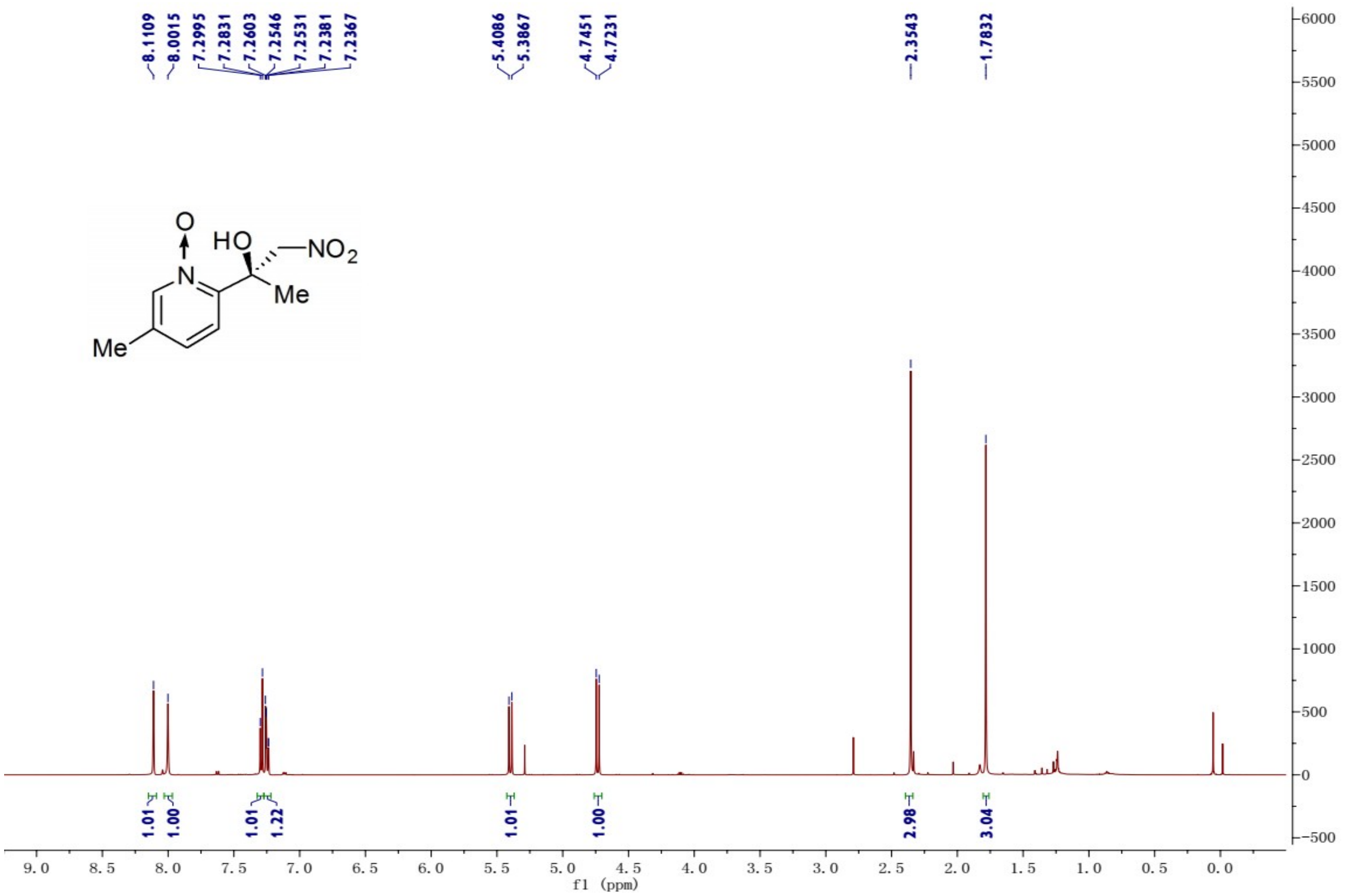

8.1109
8.0015
7.2995
7.2831
7.2603
7.2546
7.2531
7.2381
7.2367
5.4086
5.3867
4.7451
4.7231
2.3543
1.7832
HO
NO_2
N
O
Me
Me
1.01
1.00
1.01
1.22
1.01
1.00
2.98
3.04
f1 (ppm)

4d

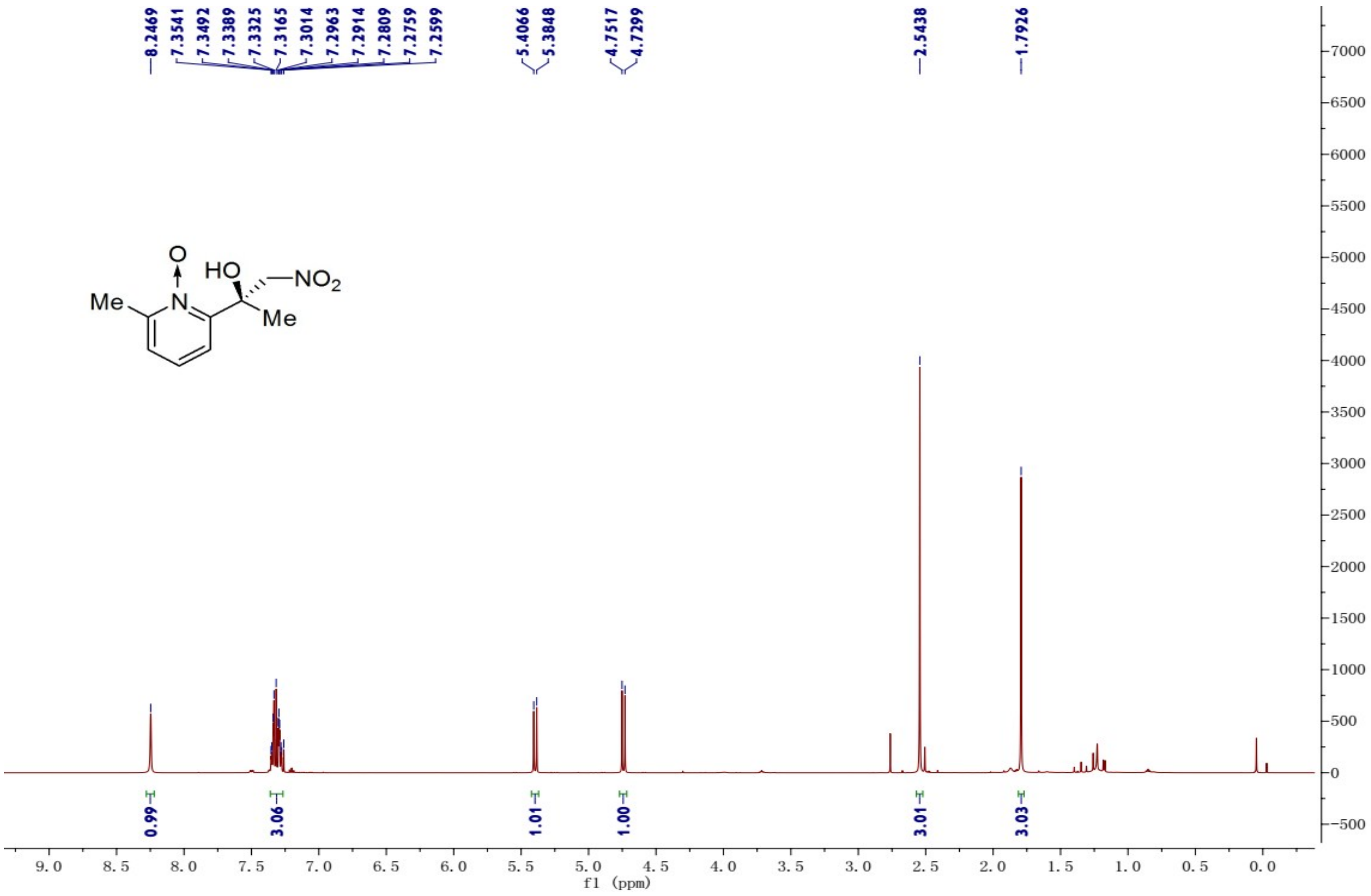
8.2469
7.3541
7.3492
7.3389
7.3325
7.3165
7.3014
7.2963
7.2914
7.2809
7.2759
7.2599
5.4066
5.3848
4.7517
4.7299
2.5438
1.7926
HO
NO2
Me
N
O
Me
0.99
3.06
1.01
1.00
3.01
3.03
f1 (ppm)

4f

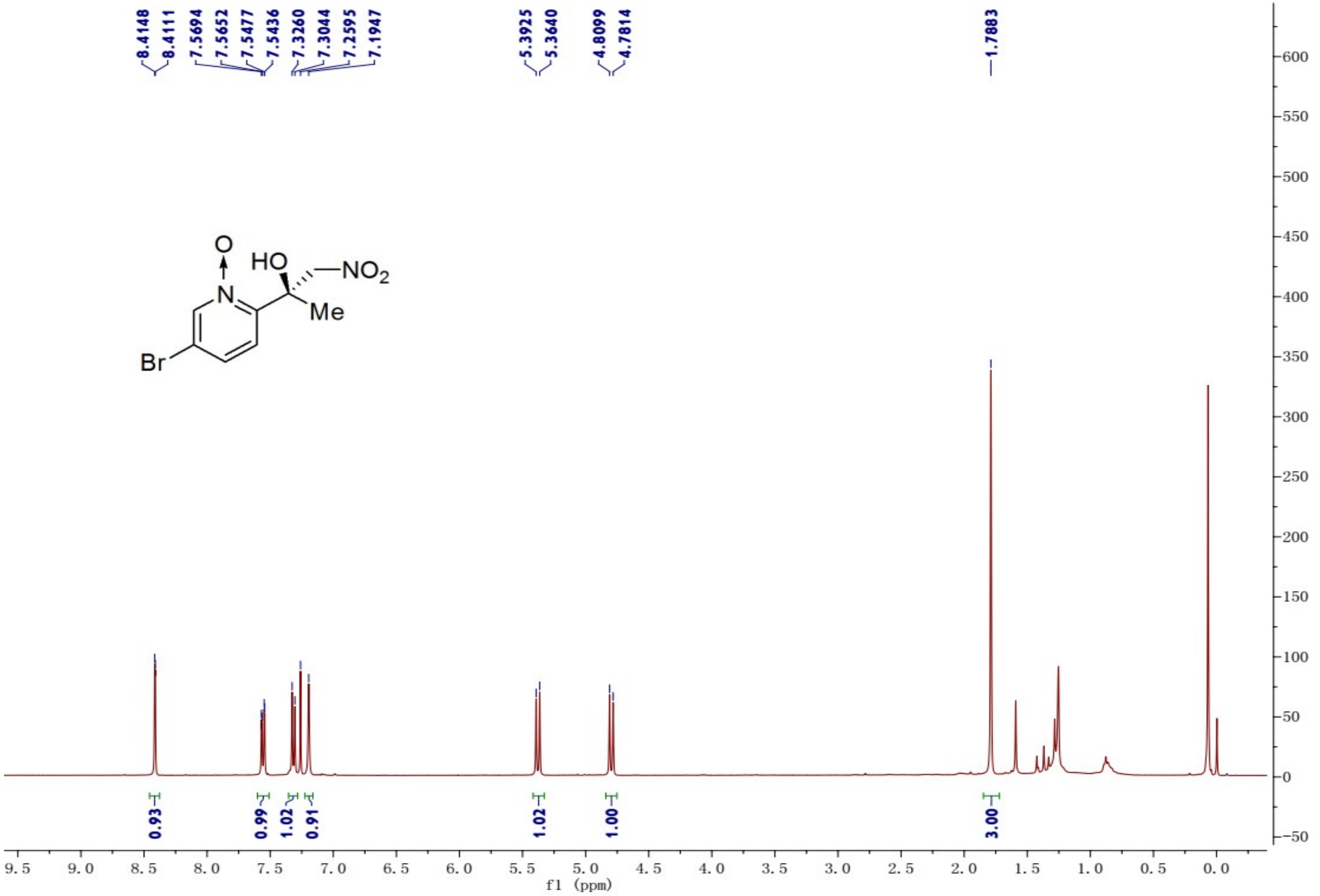
8.4148
8.4111
7.5694
7.5652
7.5477
7.5436
7.3260
7.3044
7.2595
7.1947
5.3925
5.3640
4.8099
4.7814
1.7883
HO
NO2
N
O
Me
Br
0.93
0.99
1.02
0.91
1.02
1.00
3.00
f1 (ppm)

4g

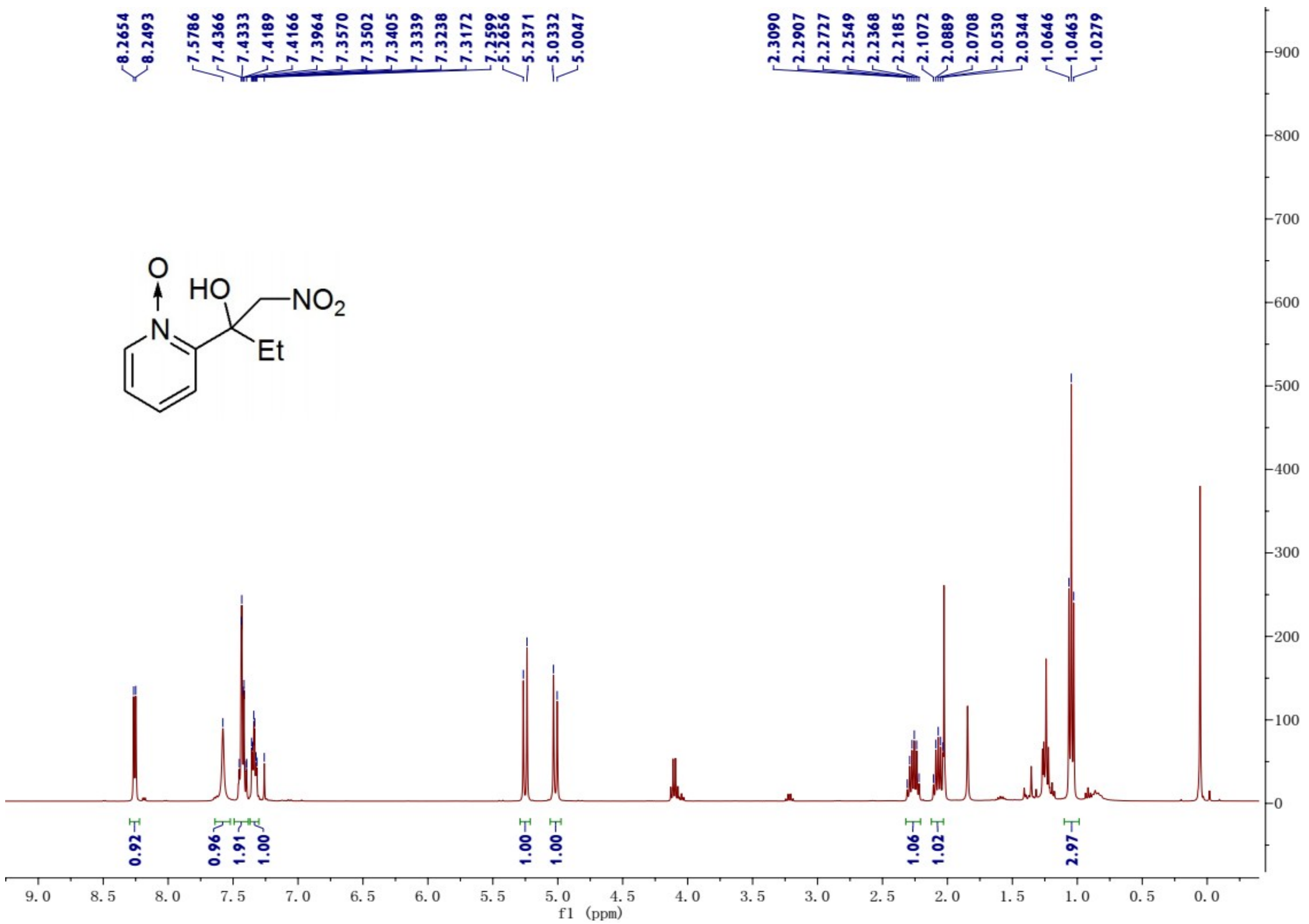
8.2654
8.2493
7.5786
7.4366
7.4333
7.4189
7.4166
7.3964
7.3570
7.3502
7.3405
7.3339
7.3238
7.3172
7.2599
5.2656
5.2371
5.0332
5.0047
2.3090
2.2907
2.2727
2.2549
2.2368
2.2185
2.1072
2.0889
2.0708
2.0530
2.0344
1.0646
1.0463
1.0279
HO
NO_2
N
O
Et
0.92
0.96
1.91
1.00
1.00
1.00
1.06
1.02
2.97
f1 (ppm)

6a

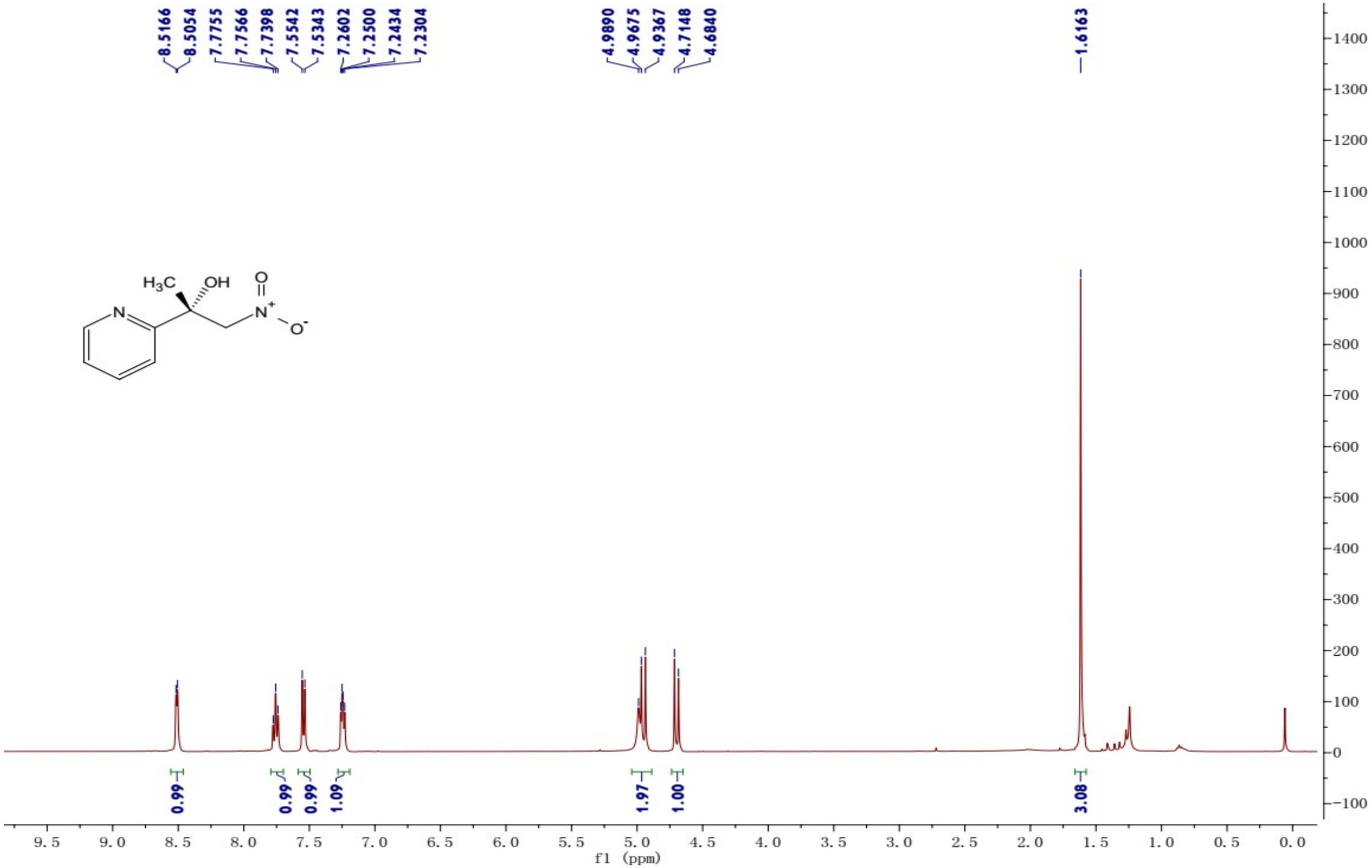
8.5166
8.5054
7.7755
7.7566
7.7398
7.5542
7.5343
7.2602
7.2500
7.2434
7.2304
4.9890
4.9675
4.9367
4.7148
4.6840
1.6163
0.99
0.99
0.99
1.09
1.97
1.00
3.08
f1 (ppm)

6a

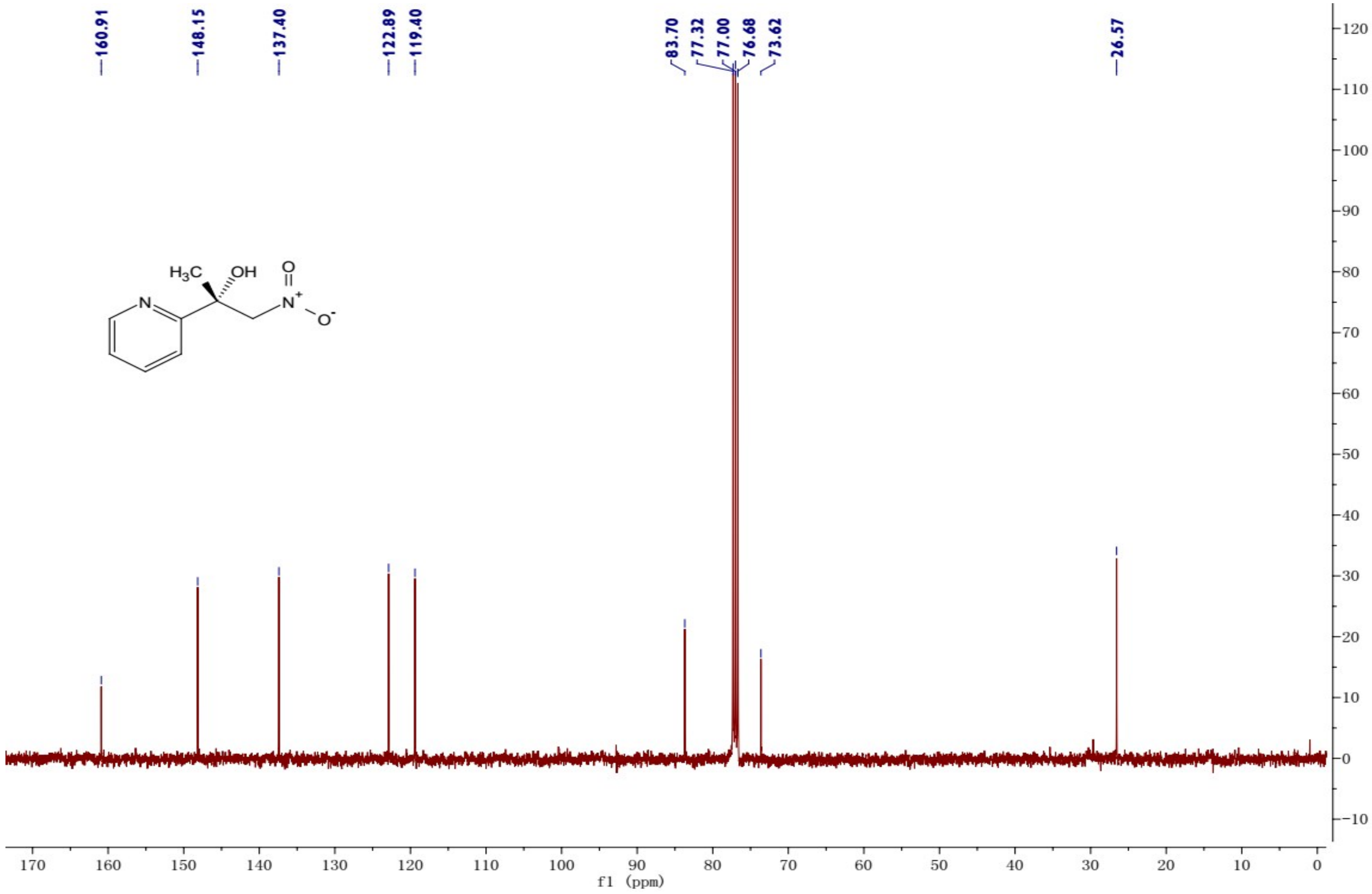

160.91
148.15
137.40
122.89
119.40
83.70
77.32
77.00
76.68
73.62
26.57
f1 (ppm)

6b

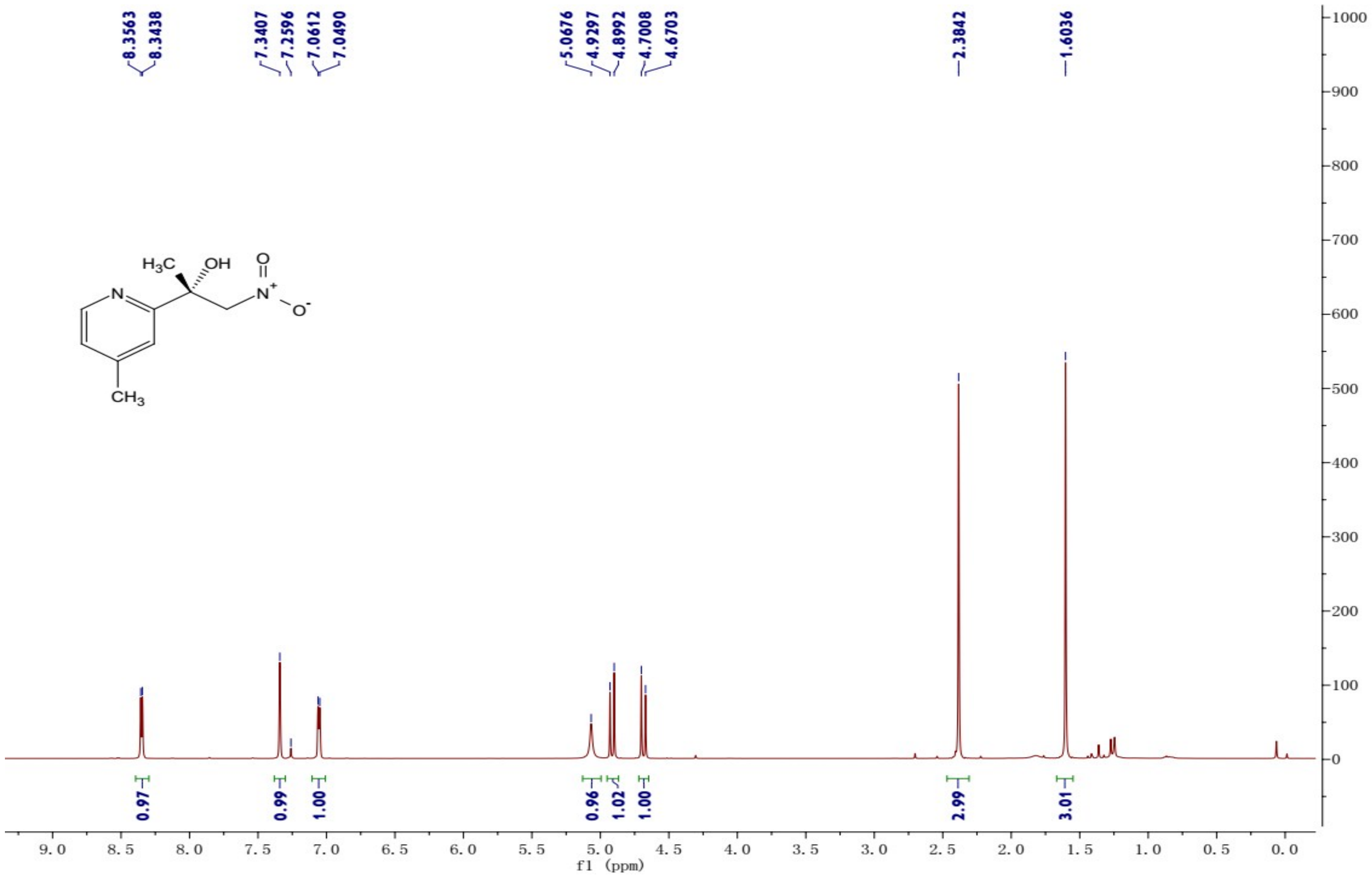
8.3563
8.3438
7.3407
7.2596
7.0612
7.0490
5.0676
4.9297
4.8992
4.7008
4.6703
2.3842
1.6036
H3C
OH
N
N+
O
O-
CH3
0.97
0.99
1.00
0.96
1.02
1.00
2.99
3.01
9.0
8.5
8.0
7.5
7.0
6.5
6.0
5.5
5.0
4.5
4.0
3.5
3.0
2.5
2.0
1.5
1.0
0.5
0.0
f1 (ppm)
1000
900
800
700
600
500
400
300
200
100
0

6b

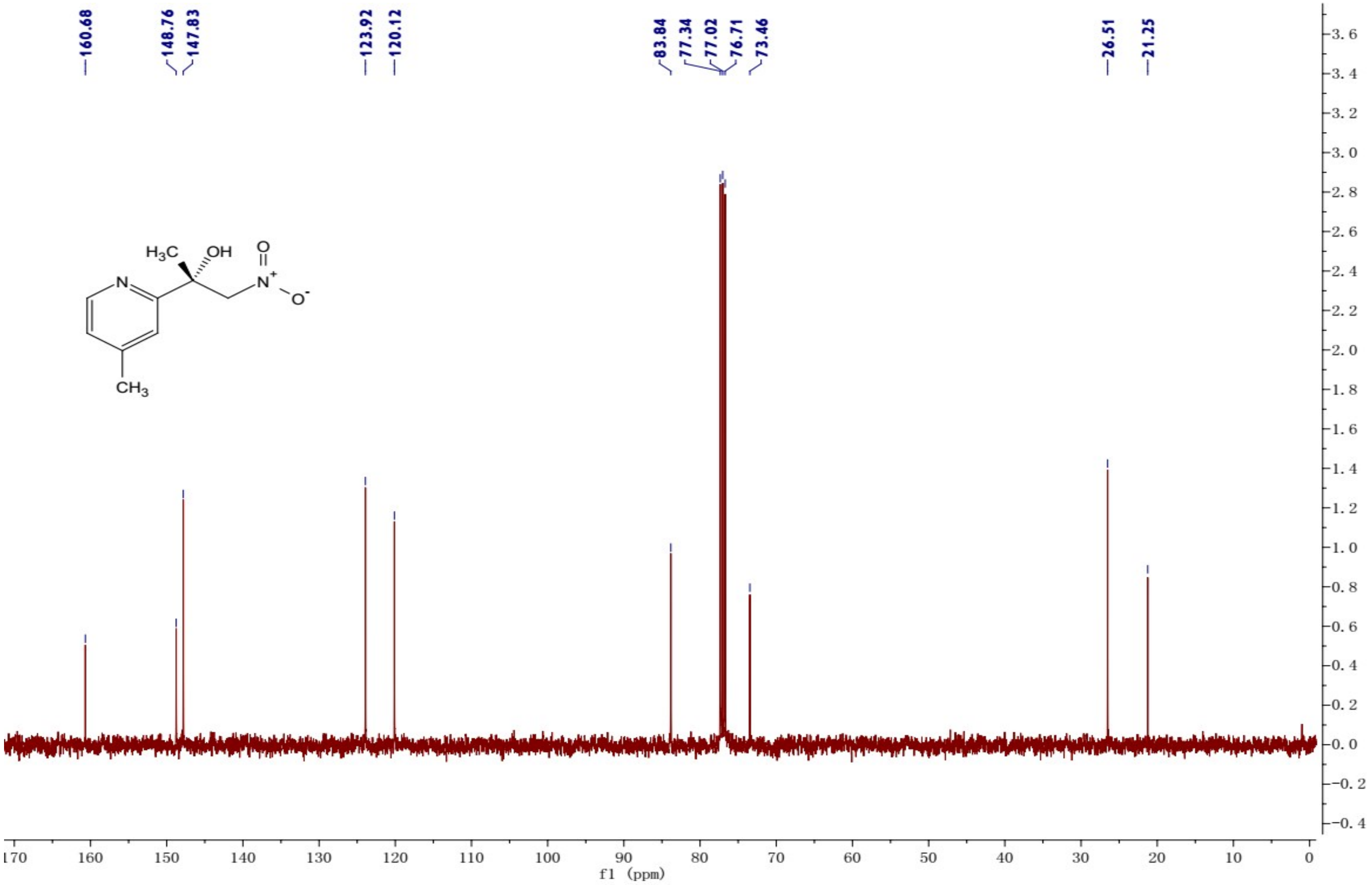
160.68
148.76
147.83
123.92
120.12
83.84
77.34
77.02
76.71
73.46
26.51
21.25
f1 (ppm)

6c

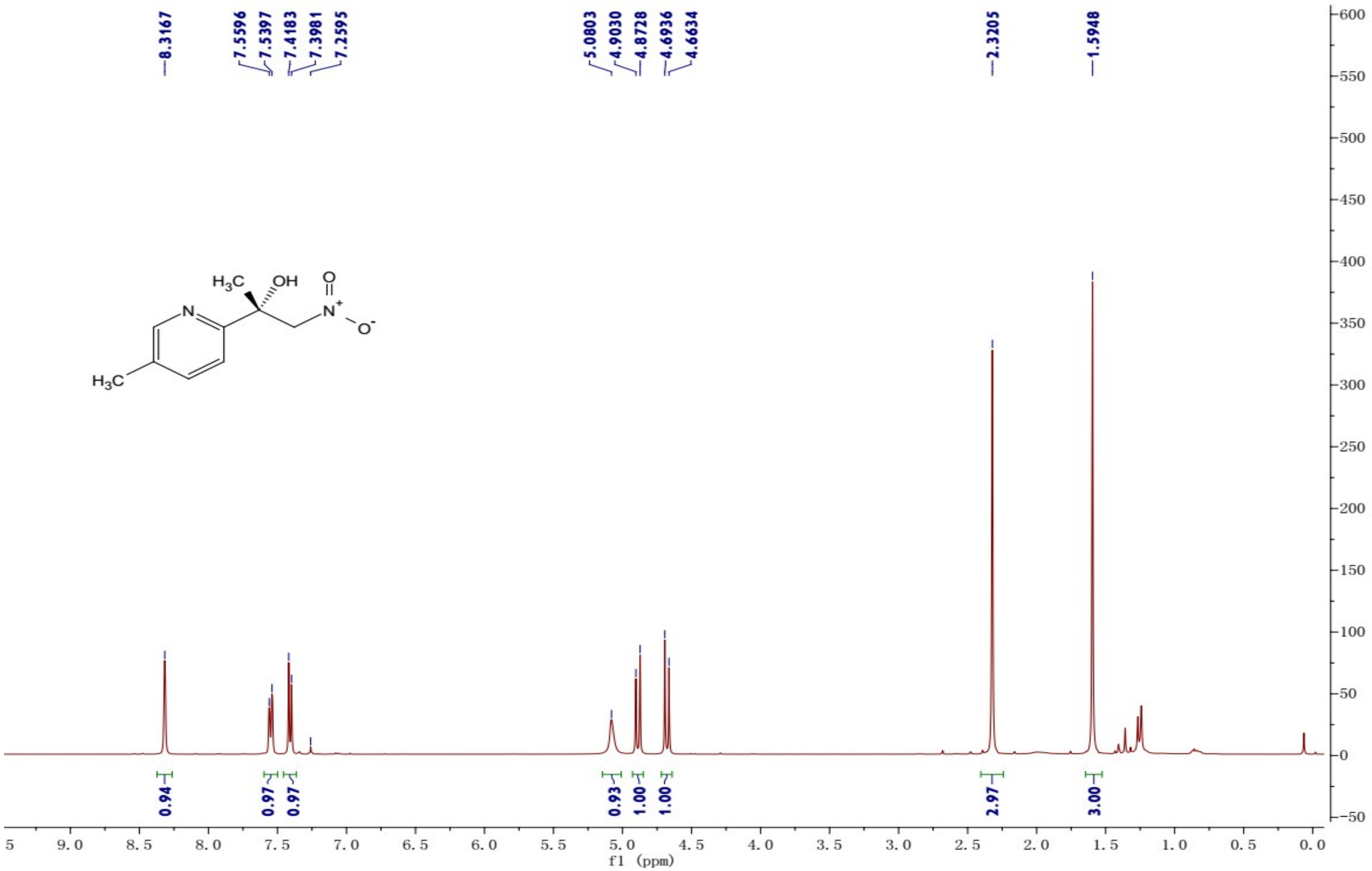

8.3167
7.5596
7.5397
7.4183
7.3981
7.2595
5.0803
4.9030
4.8728
4.6936
4.6634
2.3205
1.5948
H3C
OH
N
N+
O
O-
H3C
0.94
0.97
0.97
0.93
1.00
1.00
2.97
3.00
9.0 8.5 8.0 7.5 7.0 6.5 6.0 5.5 5.0 4.5 4.0 3.5 3.0 2.5 2.0 1.5 1.0 0.5 0.0
f1 (ppm)
600 550 500 450 400 350 300 250 200 150 100 50 0 -50

6c

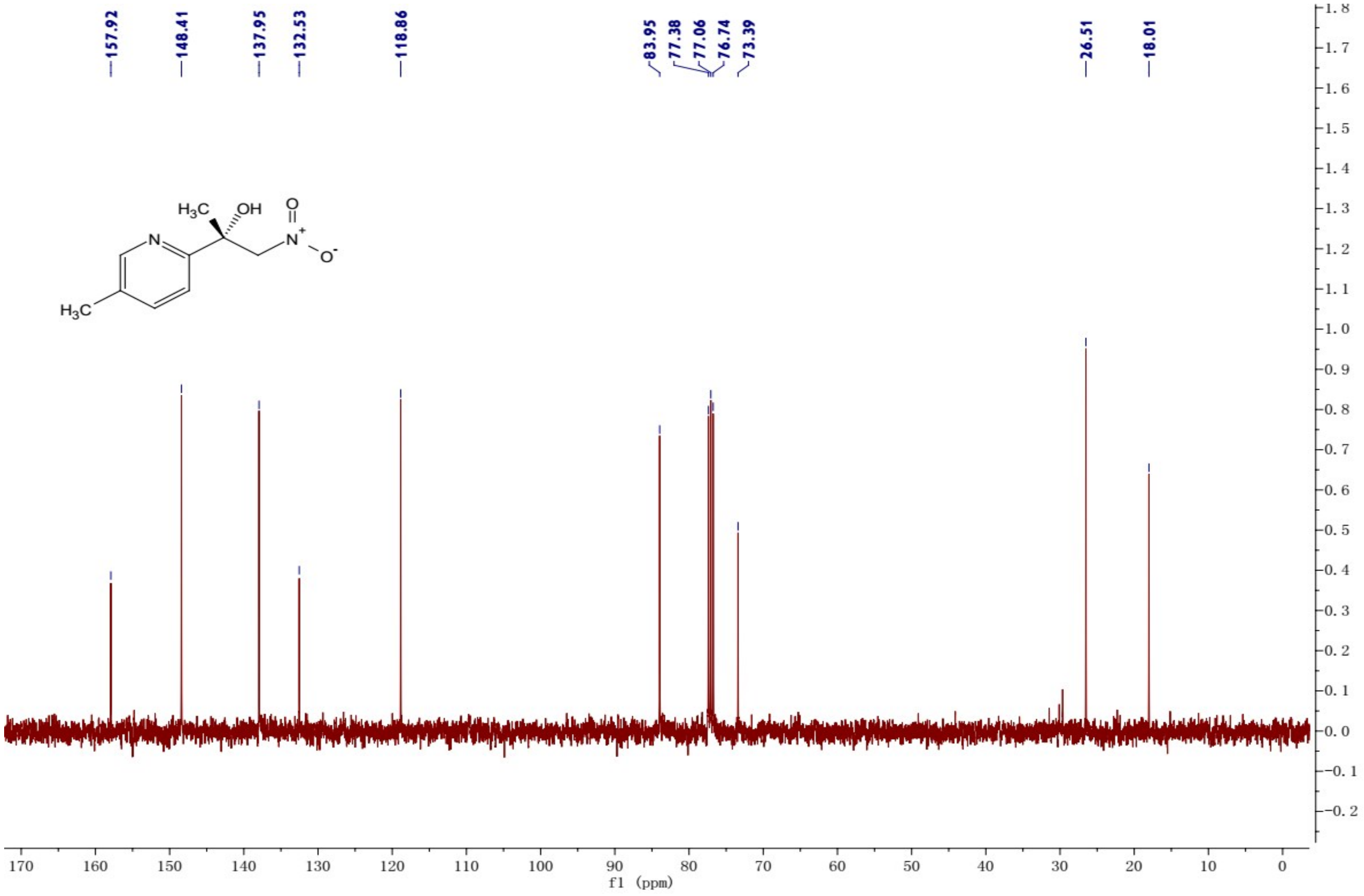
157.92
148.41
137.95
132.53
118.86
83.95
77.38
77.06
76.74
73.39
26.51
18.01
H3C
OH
O
N
N+
O-
H3C
170 160 150 140 130 120 110 100 90 80 70 60 50 40 30 20 10 0
f1 (ppm)
1.8 1.7 1.6 1.5 1.4 1.3 1.2 1.1 1.0 0.9 0.8 0.7 0.6 0.5 0.4 0.3 0.2 0.1 0.0 -0.1 -0.2

6d

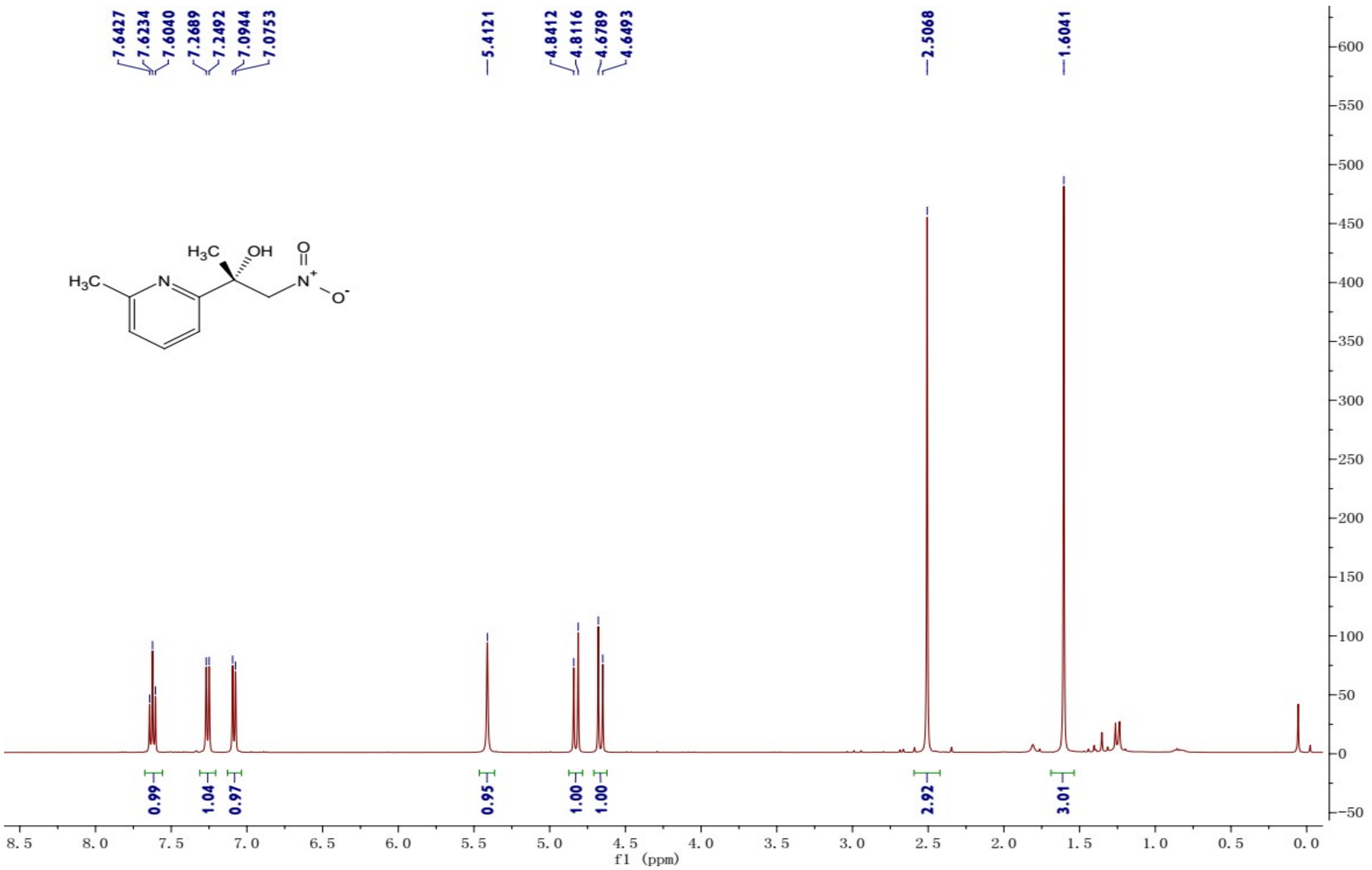

7.6427
7.6234
7.6040
7.2689
7.2492
7.0944
7.0753
5.4121
4.8412
4.8116
4.6789
4.6493
2.5068
1.6041
H_3C
OH
O
N
N^+
O^-
0.99
1.04
0.97
0.95
1.00
1.00
2.92
3.01
8.5 8.0 7.5 7.0 6.5 6.0 5.5 5.0 4.5 4.0 3.5 3.0 2.5 2.0 1.5 1.0 0.5 0.0
f1 (ppm)
600 550 500 450 400 350 300 250 200 150 100 50 0 -50

6d

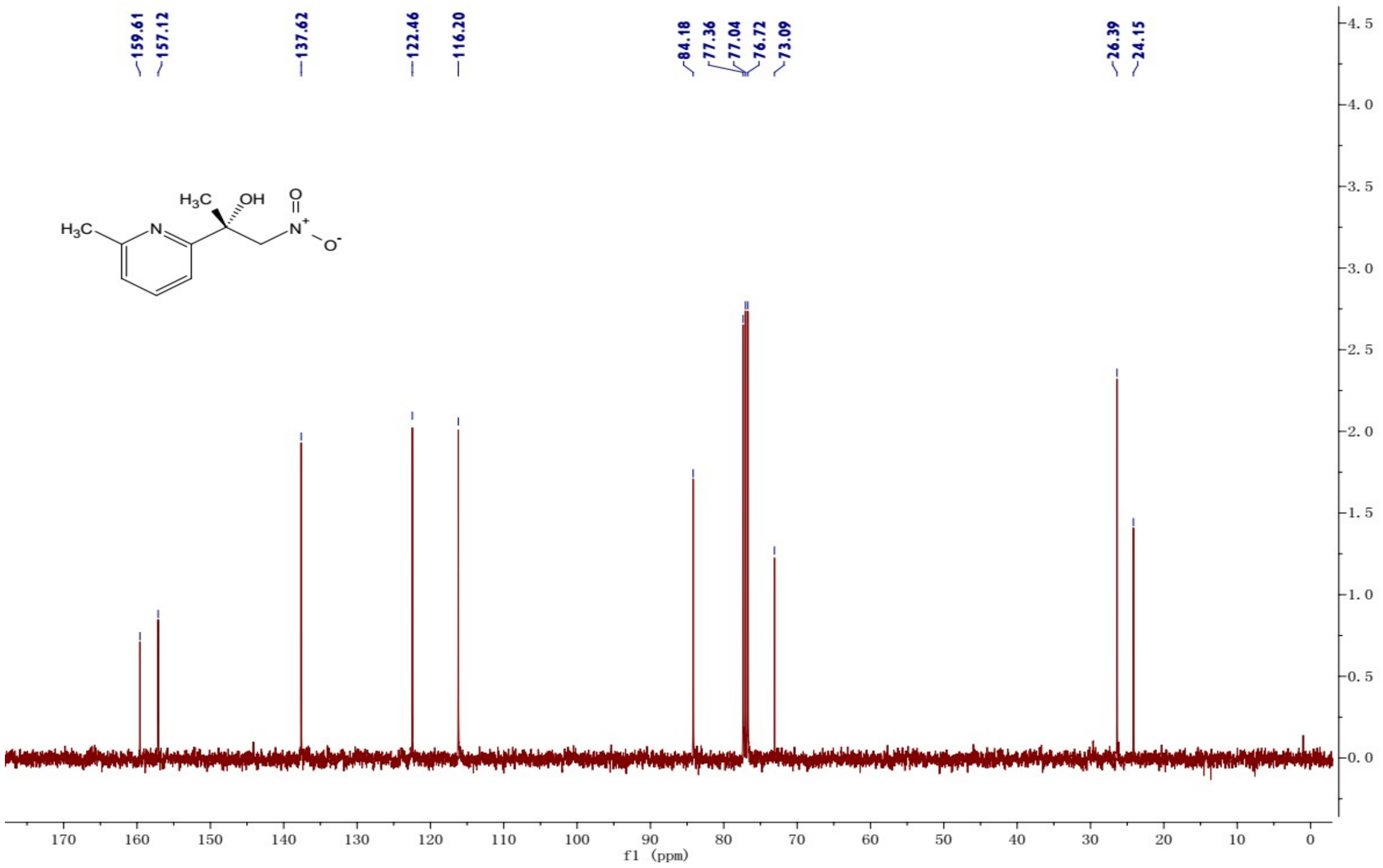

159.61
157.12
137.62
122.46
116.20
84.18
77.36
77.04
76.72
73.09
26.39
24.15
H3C
OH
N
O
N+
O-
f1 (ppm)

6f

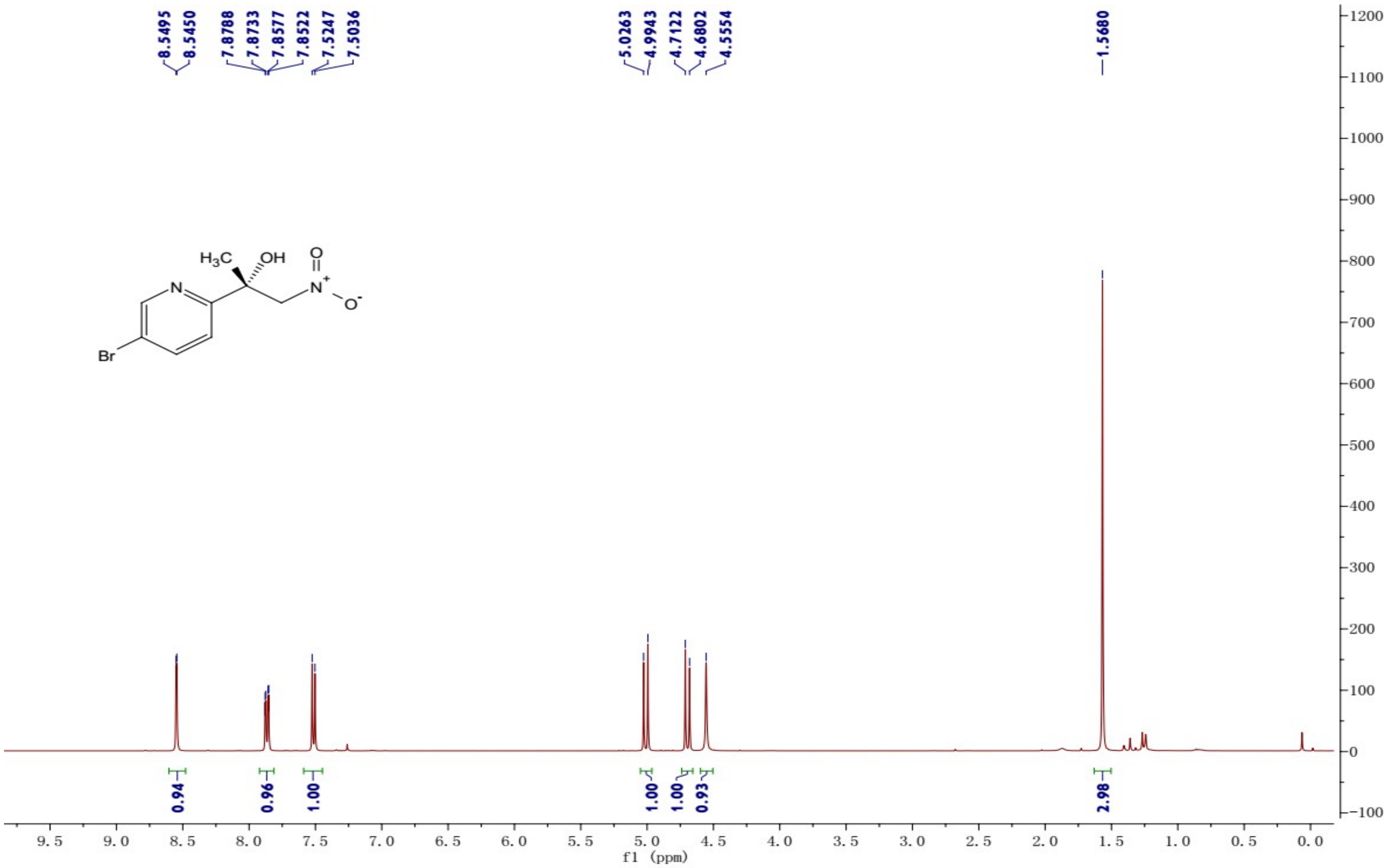

8.5495
8.5450
7.8788
7.8733
7.8577
7.8522
7.5247
7.5036
5.0263
4.9943
4.7122
4.6802
4.5554
1.5680
0.94
0.96
1.00
1.00
1.00
0.93
2.98
f1 (ppm)

6f

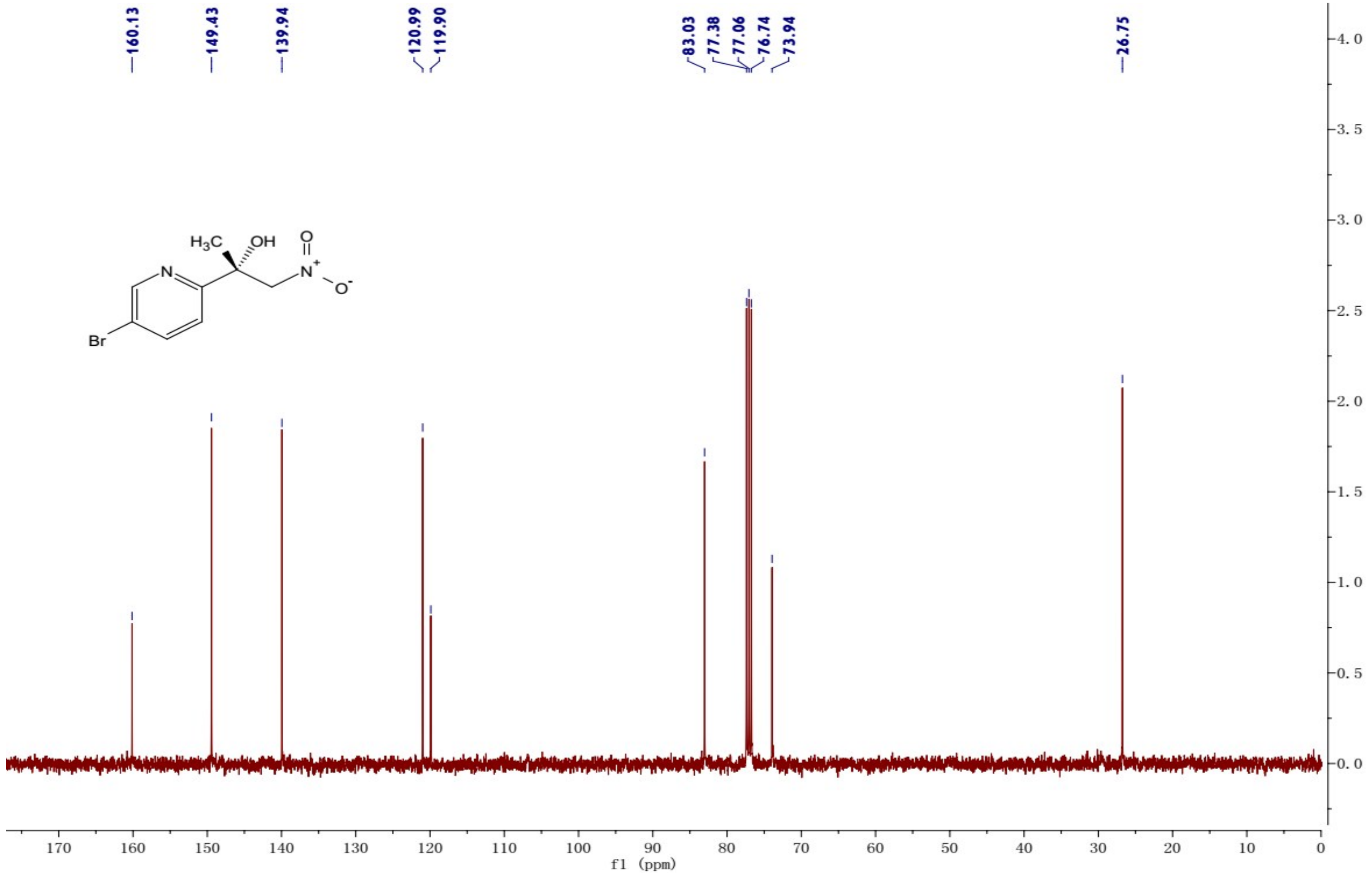
160.13
149.43
139.94
120.99
119.90
83.03
77.38
77.06
76.74
73.94
26.75
H_3C
OH
N
Br
f1 (ppm)
170 160 150 140 130 120 110 100 90 80 70 60 50 40 30 20 10 0
4.0 3.5 3.0 2.5 2.0 1.5 1.0 0.5 0.0

6g

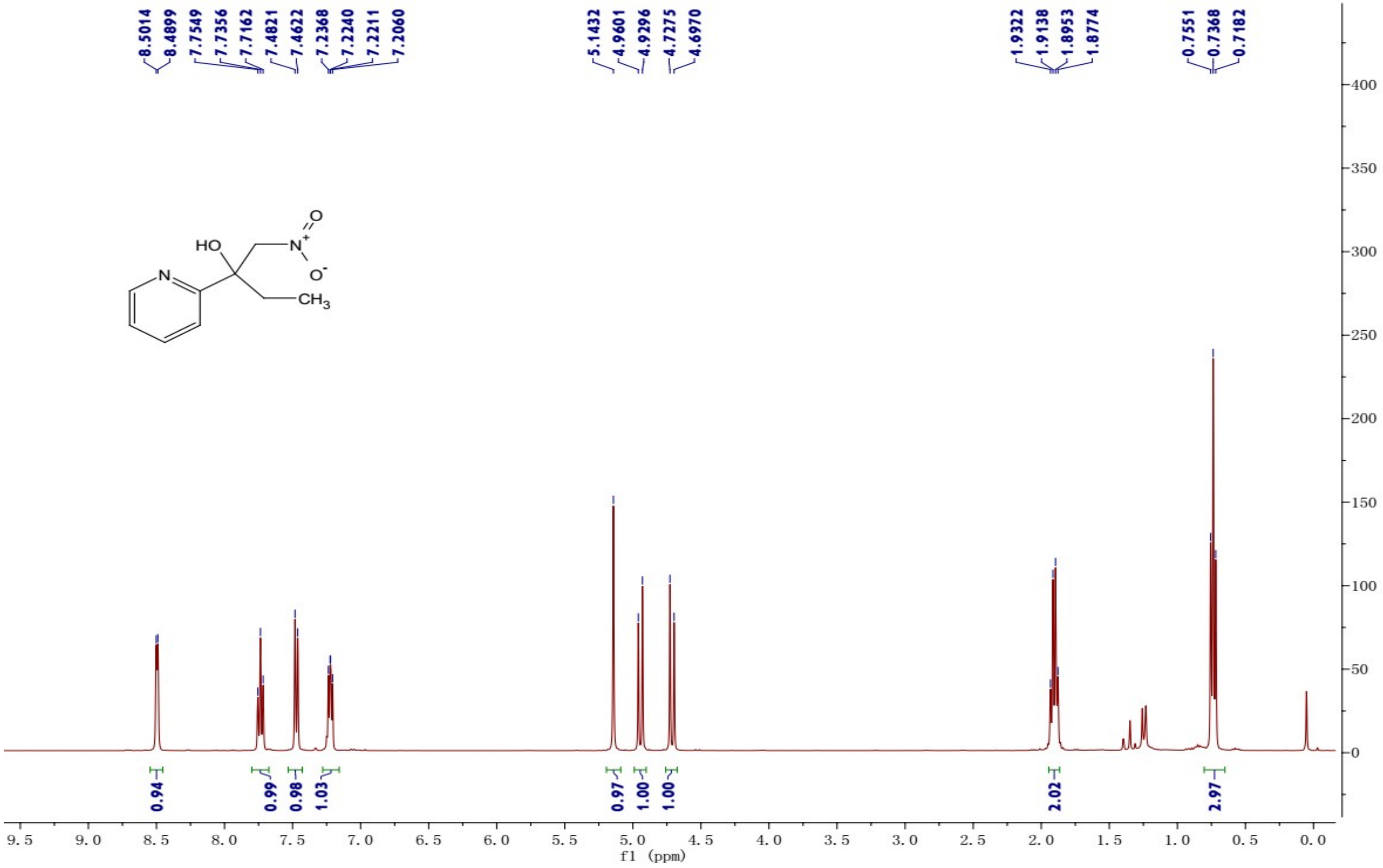
8.5014
8.4899
7.7549
7.7356
7.7162
7.4821
7.4622
7.2368
7.2240
7.2211
7.2060
5.1432
4.9601
4.9296
4.7275
4.6970
1.9322
1.9138
1.8953
1.8774
0.7551
0.7368
0.7182
HO
N
O
O-
CH3
0.94
0.99
0.98
1.03
0.97
1.00
1.00
2.02
2.97
f1 (ppm)

6g

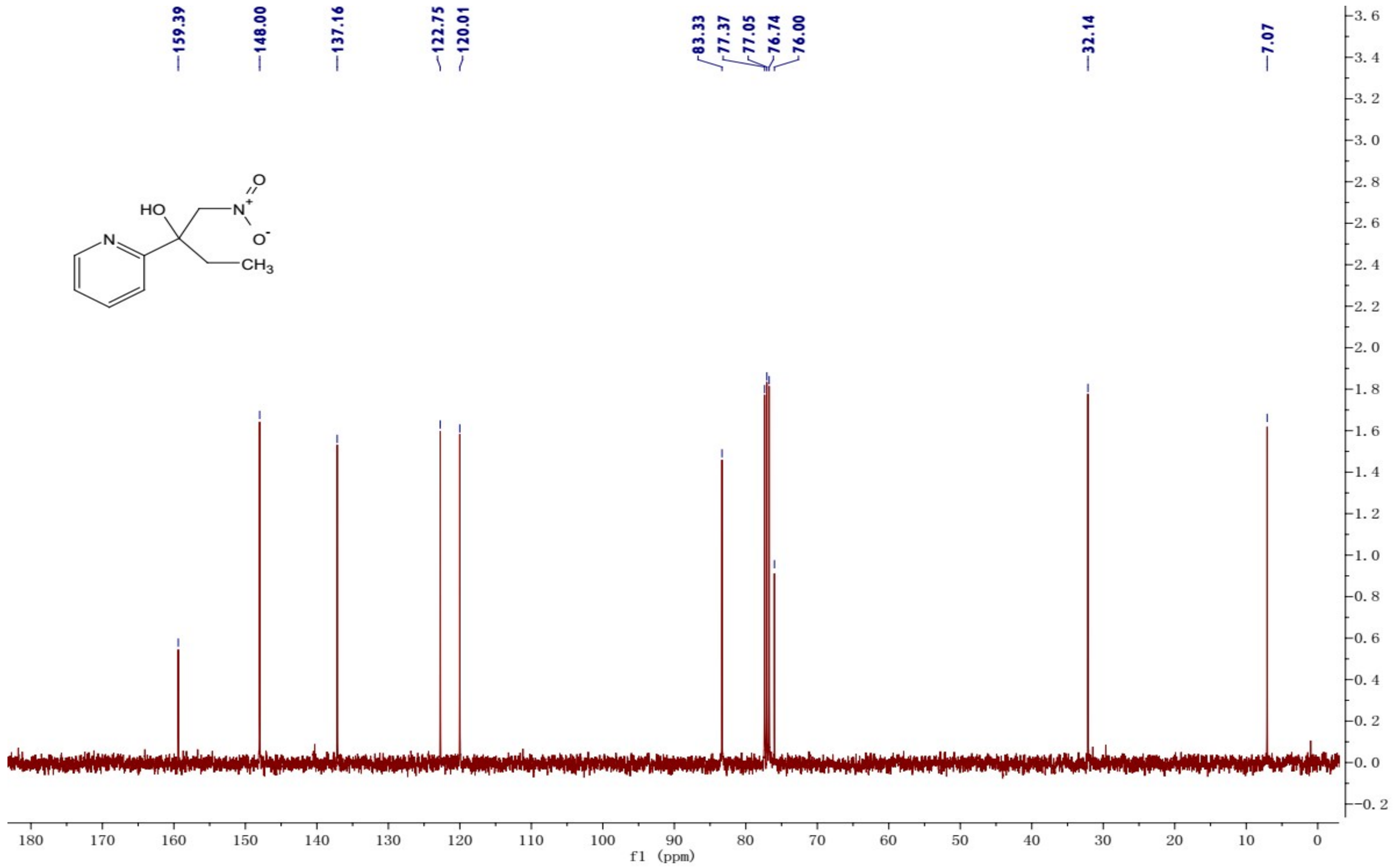

159.39
148.00
137.16
122.75
120.01
83.33
77.37
77.05
76.74
76.00
32.14
7.07
HO
N
CH3
f1 (ppm)

7

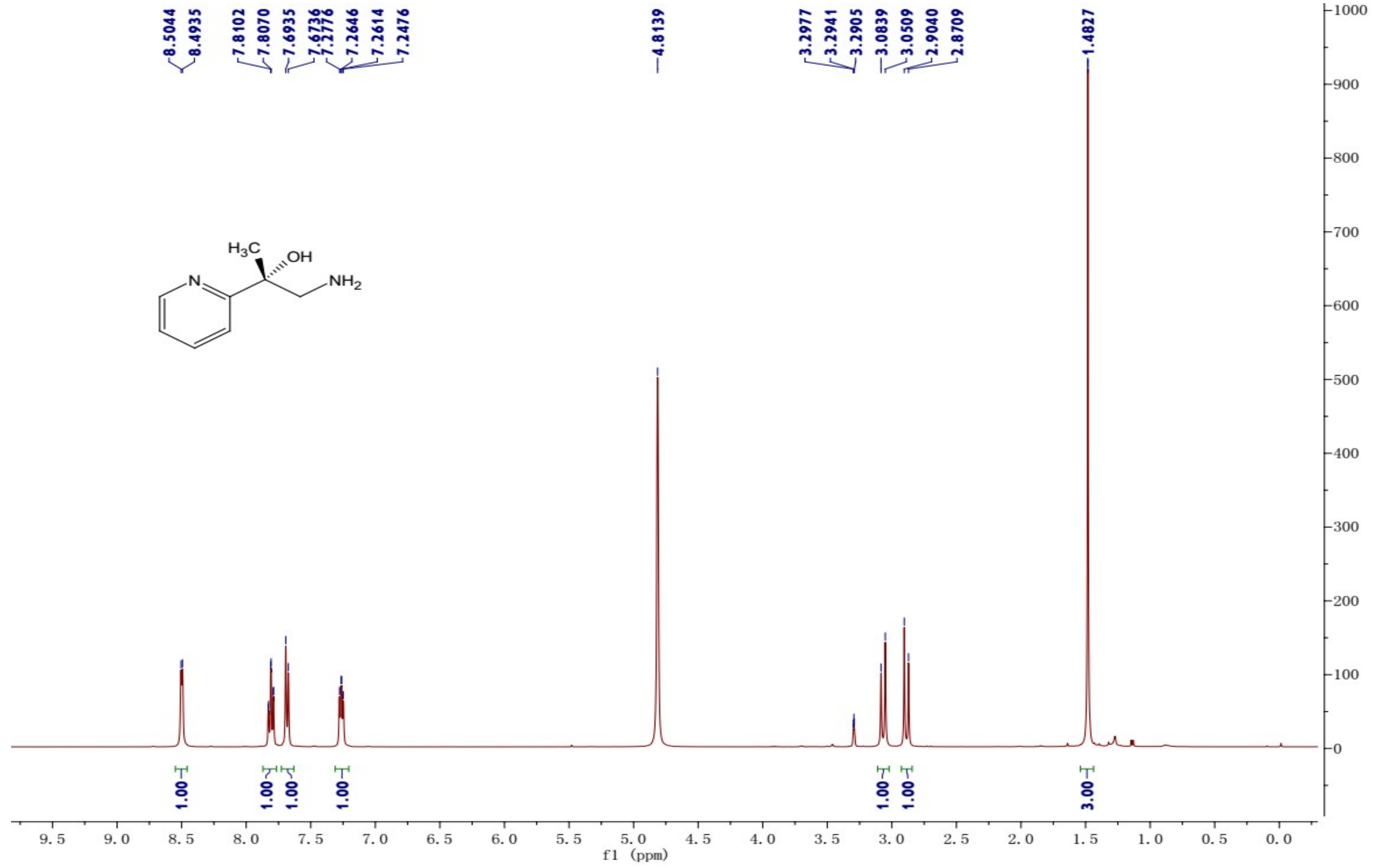

8.5044
8.4935
7.8102
7.8070
7.6935
7.6736
7.2776
7.2646
7.2614
7.2476
4.8139
3.2977
3.2941
3.2905
3.0839
3.0509
2.9040
2.8709
1.4827
H3C
OH
N
NH2
1.00
1.00
1.00
1.00
1.00
1.00
3.00
1000
900
800
700
600
500
400
300
200
100
0
9.5
9.0
8.5
8.0
7.5
7.0
6.5
6.0
5.5
5.0
4.5
4.0
3.5
3.0
2.5
2.0
1.5
1.0
0.5
0.0
f1 (ppm)

7

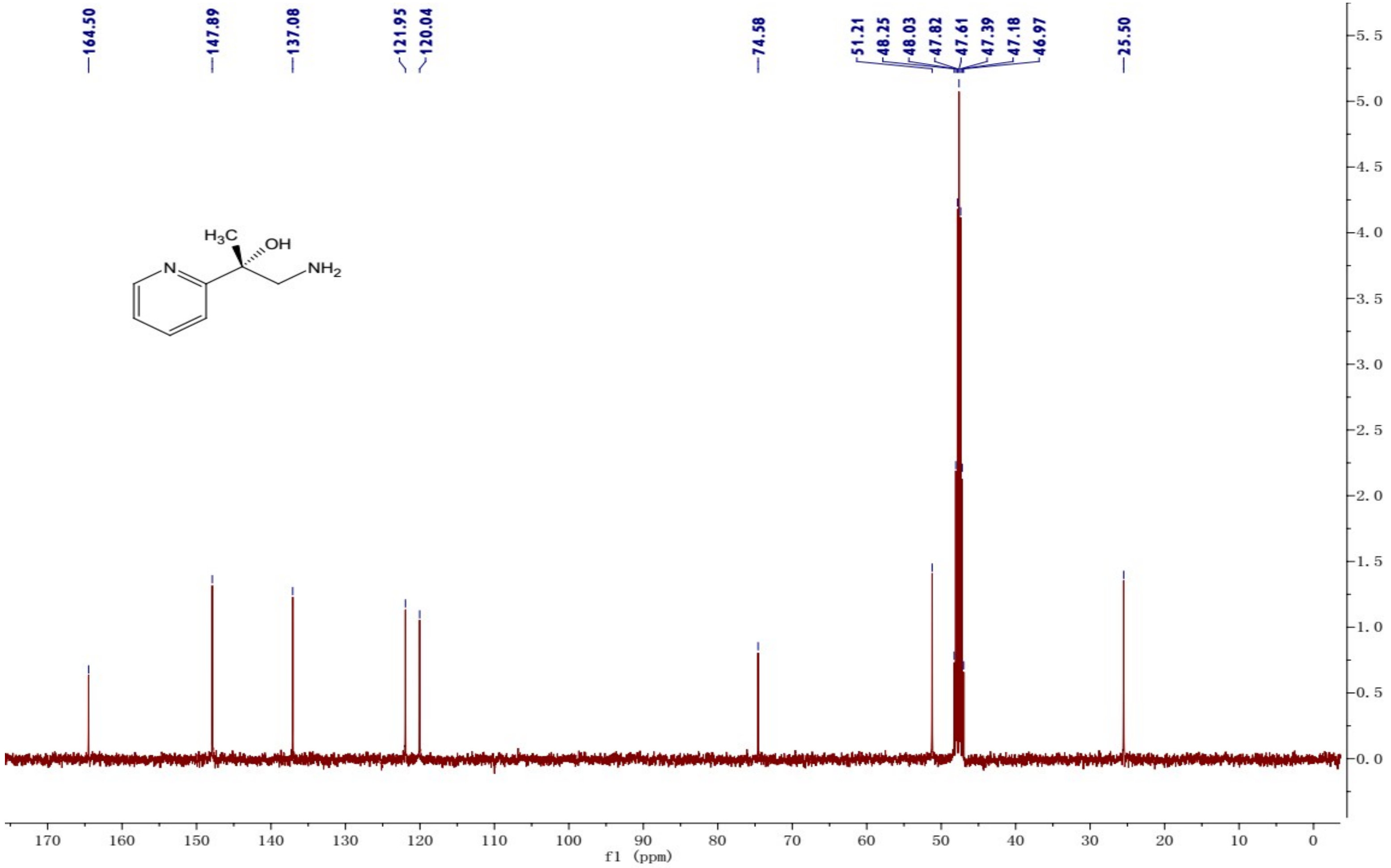

164.50
147.89
137.08
121.95
120.04
74.58
51.21
48.25
48.03
47.82
47.61
47.39
47.18
46.97
25.50
H3C
OH
N
NH2
f1 (ppm)

5. X-ray crystal structure of 2ac

Crystal data for **2ac**: $C_{12}H_{15}NO_5$, $M = 253.25$, $a = 5.66420(10)$ Å, $b = 9.5973(2)$ Å, $c = 22.6622(4)$ Å, $\alpha = 90°$, $\beta = 90°$, $\gamma = 90°$, $V = 1231.94(4)$ Å^3, $T = 100(2)$ K, space group $P212121$, $Z = 4$, μ(CuKα) = 0.903 mm^{-1}, 6426 reflections measured, 2247 independent reflections ($R_{int} = 0.0357$). The final R_1 values were 0.0331 ($I > 2\sigma(I)$). The final $wR(F^2)$ values were 0.1039 ($I > 2\sigma(I)$). The final R_1 values were 0.0336 (all data). The final $wR(F^2)$ values were 0.1046 (all data). The goodness of fit on F^2 was 1.184. Flack parameter = 0.05(5).

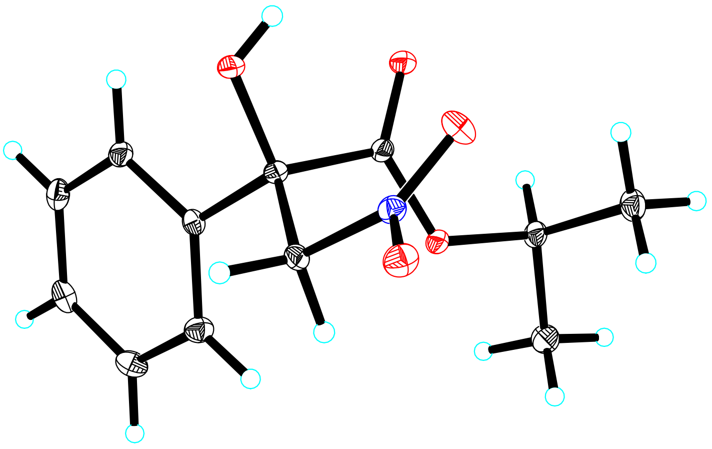

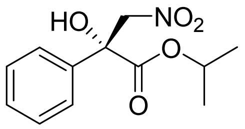

2ac

CCDC No. 1564222

View of a molecule of **2ac** with the atom-labelling scheme

Displacement ellipsoids are drawn at the 30% probability level

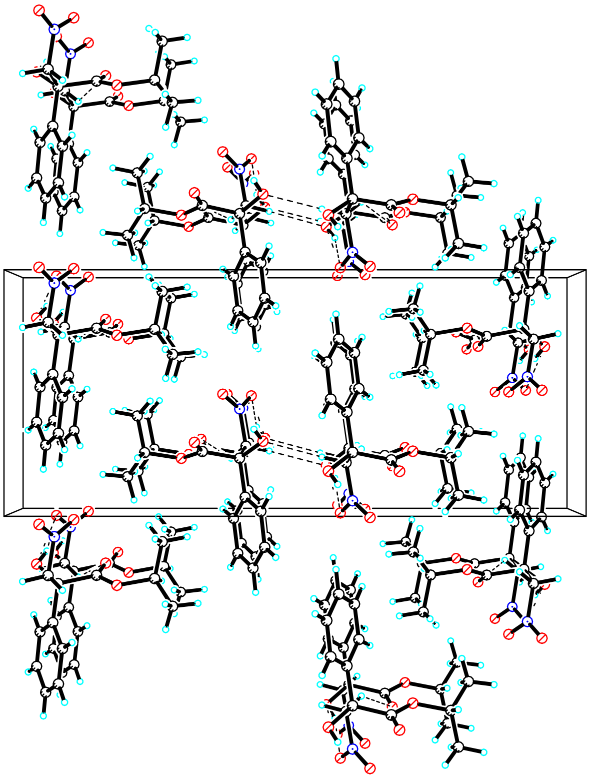

View of the pack drawing of **2ac**

Hydrogen-bonds are shown as dashed lines
